# Exploring the Geometric Space of Metal–Organic Polyhedrons (MOPs) of Metal-Oxo Clusters

**DOI:** 10.1021/acs.inorgchem.1c01987

**Published:** 2021-09-22

**Authors:** Balamurugan Kandasamy, Edward Lee, De-Liang Long, Nicola Bell, Leroy Cronin

**Affiliations:** School of Chemistry, The University of Glasgow, Glasgow G12 8QQ, U.K.

## Abstract

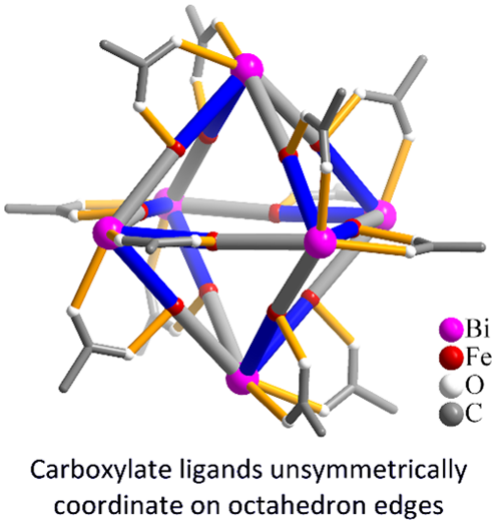

Metal organic polyhedra (MOPs) such as coordination cages and clusters are increasingly utilized across many fields, but their geometrically selective assembly during synthesis is nontrivial. When ligand coordination along these polyhedral edges is arranged in an unsymmetrical mode or the bridging ligand itself is nonsymmetric, a vast combinatorial space of potential isomers exists complicating formation and isolation. Here we describe two generalizable combinatorial methodologies to explore the geometrical space and enumerate the configurational isomers of MOPs with discrimination of the chiral and achiral structures. The methodology has been applied to the case of the octahedron {Bi_6_Fe_13_L_12_} which has unsymmetrical coordination of a carboxylate ligand (L) along its edges. For these polyhedra, the enumeration methodology revealed 186 distinct isomers, including 74 chiral pairs and 38 achiral. To explore the programming of these, we then used a range of ligands to synthesize several configurational isomers. Our analysis demonstrates that ligand halo-substituents influence isomer symmetry and suggests that more symmetric halo-substituted ligands counterintuitively yield lower symmetry isomers. We performed mass spectrometry studies of these {Bi_6_Fe_13_L_12_} clusters to evaluate their stability and aggregation behavior in solution and the gas phase showing that various isomers have different levels of aggregation in solution.

## Abstract 2

We describe combinatorial methodologies to explore the geometrical space and enumerate the configurational isomers of metal organic polyhedra with discrimination of the chiral and achiral structures. The methodology was applied to the octahedral {Bi_6_Fe_13_L_12_} which has an unsymmetrical coordination of a carboxylate ligands (L) along its edges. For these polyhedra, the enumeration methodology revealed 186 distinct isomers, including 74 chiral pairs and 38 achiral. We used a range of ligands to synthesize several configurational isomers.

## Introduction

Coordination-driven self-assembly, which relies on specific metal–ligand interactions, is a powerful method to make several distinct classes of supramolecular coordination complexes (SCCs), metal–organic polyhedra (MOPs), and metal–organic frameworks (MOFs).^1−6^ This design needs the combination of both “donor” organic bridging ligands with suitable “acceptor” metal ions or discrete metal-oxo cluster corners to yield a variety of architectures.^7−9^ By exploiting metal atoms and metal-oxo clusters as nodes and with symmetric bridging ligands as linkers, a range of self-assembled structures, with specific configurations and conformations of SCCs, MOPs, and MOFs have been rationally synthesized.^10^ Using this approach numerous nodes and organic linkers have been investigated in the past, but the use of nonsymmetric bridging ligands (i.e., ambidentate ligands) yielding unsymmetric polyhedral edges during the self-assembly process is very limited.^11^ This is because ambidentate ligands may connect the inorganic nodes in several modes and geometries giving a vast number of self-assembled structures.^6^ As such, the complexity and functionality increase exponentially because of nearly isoenergetic combinations of different orientations of the ligands producing numerous configurational isomers.

**Figure 1:**
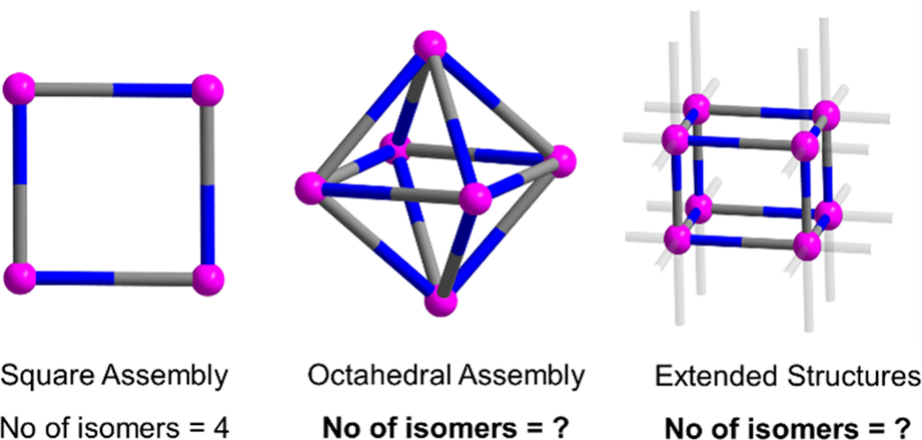
Schematic representation of isomers of polygon M_4_L_4_, polyhedral {M_6_L_12_} cage, and extended structures such as MOFs. The nonsymmetric bridging ligands L (blue and gray) linking metal ions M (pink) offer variable isomers depending on their orientations.

A critical question is for how many isomers one may expect an octahedral cage to form when the polyhedral edges are unsymmetrical; see Figure 1. Mathematically, it is possible to employ combinatorial methods to enumerate all the configurational isomers of specified components.^12−16^ Investigation may provide insights regarding the reactivity, synthetic accessibility, and potential for supramolecular aggregation of each individual isomer.

Ambidentate ligands can easily be demonstrated to produce numerous configurational isomers of MOPs; however, symmetrical ligands, if unsymmetrically arranged on polyhedron edges, can also yield various isomers. A good example of the formation of analogous types of polyhedral structures in solution is molecular iron-oxides.^17−19^ Recently, Nyman and co-workers reported the stabilization of an iron-oxo cluster in water by wrapping it with large Bi^3+^ ions and carboxylate ligands, producing the discrete octahedral-shaped [Bi_6_Fe_13_O_16_(OH)_12_(CF_3_CO_2_)_12_]^+^ (**Keggin-2**) and [Bi_6_Fe_13_O_16_(OH)_12_(CCl_3_CO_2_)_12_]^+^ (**Keggin-3**) clusters.^20,21^ These were the only examples of large, purely Fe_13_-oxo Keggin clusters being stabilized in water. Both clusters have carboxylate ligands appended unsymmetrically across the edge of the octahedron, demonstrating the possibility of producing vast numbers of configurational isomers of homologues (see Figure S1).

Herein, we demonstrate the theoretical enumeration of all possible configurational isomers of metal–organic polyhedron-based structures using the {Bi_6_Fe_13_L_12_} cluster as a model. To gain more insight synthetically, we have chosen different ligand substituents to manipulate the configurational isomers. By varying the type and number of halo-substituents (thus p*K*_a_) of the carboxylic acids, we show that ligand variation plays a key role during the assembly process, which affects the selection of isomers by the system. In this respect, the feasibility of rationally designing a discrete {Bi_6_Fe_13_L_12_}-type nanocluster is explored. Finally, the higher-order supramolecular aggregation of the cationic {Bi_6_Fe_13_L_12_}- type clusters in organic media was also investigated by Ion Mobility-MS, and this shows how different isomers can also be imaged in solution and in the gas phase.

## Results and Discussion

### Synthesis

**Figure 2:**
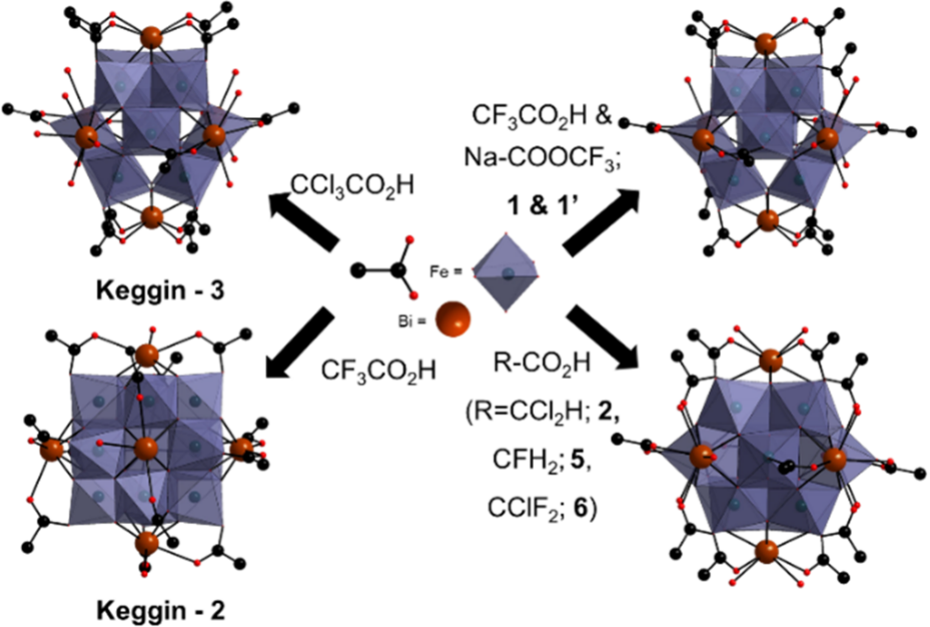
Synthesis of octahedral {Bi_6_Fe_13_L_12_} isomers with unsymmetrical ligation along the Bi···Bi edges yields complexes **1**–**6** or **Keggin 2–3** depending upon the ligand nature and synthetic conditions. Complexes are depicted as polyhedral or ball and stick to clarify ion and ligand positions.

The one-pot reaction between metal (Bi and Fe) nitrates and excess halo-acetic acids in an aqueous acidic medium, followed by deprotonation using metal carbonates, (pH < 3.5) resulted in a family of [Bi_6_Fe_13_O_16_(OH)_12_L_12_]^+^-type clusters (L = CF_3_CO_2_^–^**1** and **1′**; CHCl_2_CO_2_^–^**2**; CHF_2_CO_2_^–^**3**; CH_2_ClCO_2_^–^**4**; CH_2_FCO_2_^–^**5**; CClF_2_CO_2_^–^**6** and **6a**). Solid-state structures and the general synthetic reaction scheme for typical compounds plus **Keggin-2** and **Keggin-3**(20,21) are depicted in Figure 2. All the compounds were crystallographically characterized and were found to display an octahedral-shaped {Bi_6_Fe_13_L_12_}-type cluster. The skeleton consists of 6 Bi^3+^ ions with 12 Fe^3+^ ions centered along the Bi···Bi edges, along with a final body centered Fe^3+^ ion, yielding the general formula [Bi_6_Fe_13_O_16_(OH)_12_L_12_]^+^. The polymetallic core was stabilized by coordination from 12 carboxylate ligands bridging between each edge-centered Fe and only one of its corresponding Bi atoms meaning each edge has an unsymmetrical ligand configuration.

### Structure Determinations

All compounds obtained have been characterized by single crystal X-ray diffraction for structure determination (Tables S2 and S3), displaying an octahedral-shaped {Bi_6_Fe_13_L_12_}-type cluster. Bi and Fe centers all are in their +3 oxidation states confirmed by bond valence sum calculations (Table S4). The octahedral skeleton shows that a tetrahedral FeO_4_ central unit is encapsulated in a discrete {Fe_12_} ball, resulting in an unstable polynuclear α-Keggin [(FeO_4_)Fe_12_O_12_(OH)_12_]^5–^ oxo/hydroxyl core, whose six square faces are further capped by six Bi^3+^ ions {Bi_6_Fe_13_}. The [(FeO_4_)Fe_12_O_12_(OH)_12_]^5–^ α-Keggin ball can also be considered as constructed from four {Fe_3_} triads. Each triad is formed by three edge-shared FeO_6_ octahedra, and four of such edge-shared triads are linked together by corner-sharing polyhedra. Within the triads, the bridging ligands of the edge-sharing octahedra are hydroxyls, and the ligands between the corner-sharing octahedra are oxo ligands. The polynuclear iron core was further stabilized by coordination from 12 carboxylate ligands resulting in a univalent cationic oxo cluster, and thus it crystallizes with univalent anions. Each Bi vertex can be bridged by zero to four carboxylate ligands.

Compounds **1** and **1′** have the same compositions Na_3_[Bi_6_Fe_13_O_16_(OH)_12_(CF_3_COO)_12_](CF_3_COO)_4_, crystallizing in the monoclinic system space group *C*2/*c* and triclinic system space group *P*1, respectively, with slightly different numbers of solvated water molecules in each. Beyond the 12 coordinated ligands, four additional carboxylates were found coordinating to Na ions which in turn link to clusters by weak water bridging interactions. Three Na ions per cluster were found to balance the charge of these excess carboxylates. Compound **2** with empirical formula Na_2_[Bi_6_Fe_13_O_16_(OH)_12_(CHCl_2_COO)_12_] (CHCl_2_COO)_3_·31H_2_O crystallizes in a trigonal system with space group *R-*3. One sixth of the cluster was observed in the asymmetric unit. Na ion sites with partial occupancy were identified by judging their coordination spheres. Solvate dichloroacetate was also found and modeled in the solvent area. Compounds **3** and **4** both crystallize in the tetragonal system space group *I-*42*d*. One quarter of the cluster was found in the asymmetric unit with three ligands observed. One ligand is clear without disorder but the other two have positional disorders; thus, the isomer type of the whole cluster is unclear demonstrating the subtle energetics at play in isomer formation. Compound **5** has a very similar unit cell with those of compounds **3** and **4** but lower Laue group symmetry; therefore, an orthorhombic system prevails (Table S2). The structure was solved in the *P*2_1_2_1_2_1_ space group with crystallography *b* and *c* axes with only minor differences caused by sodium cation and nitrate anion packing along one direction. Both compounds **6** and **6a** crystallize in cubic system space group *Im-*3. One sixth of the cluster with some disordered Cs metal sites and solvent water molecules were found in the asymmetric unit.

To evaluate the crystal packing and intermolecular interactions between the clusters, we used the volume per cluster (VPC) of single crystal structures for comparison (see Table S2). Significantly, different VPCs 4230, 3573, and 2823 Å^3^ in an array were observed for **Keggin-3** and compounds **2** and **4**, which are of tri-, di-, and monochloro-substituted acetate ligands, respectively. This shows that the number and type of substituents at the α-position of acetate ligands have remarkable effects on the crystal packing model (lattice type) as well as the VPCs because of the larger Cl atomic size. Comparatively, the fluoro-substituted clusters (**Keggin-2**, compounds **1**, **1**′, **3**, and **5**) possess smaller VPCs than corresponding chloro-substituted ones. This is due to a much smaller fluorine atomic size and generally stronger intermolecular F···F or F···H interactions between clusters yielding denser packing. It should be noted that the various numbers of solvate cations and anions also contribute to the differences of VPCs, especially in the cases of variously fluoro-substituted ligands in which the F atomic size effect is not very significant compared to H substitution. **1** and **1′** each have four solvate trifluoroacetate ligands, and their VPCs are higher than those of **3** and **5**. Compound **3** has two solvate difluoroacetate ligands while **5**, the only one that crystallizes with nitrate as counteranions has the smallest VPC.

### Enumeration Methods

**Figure 3:**
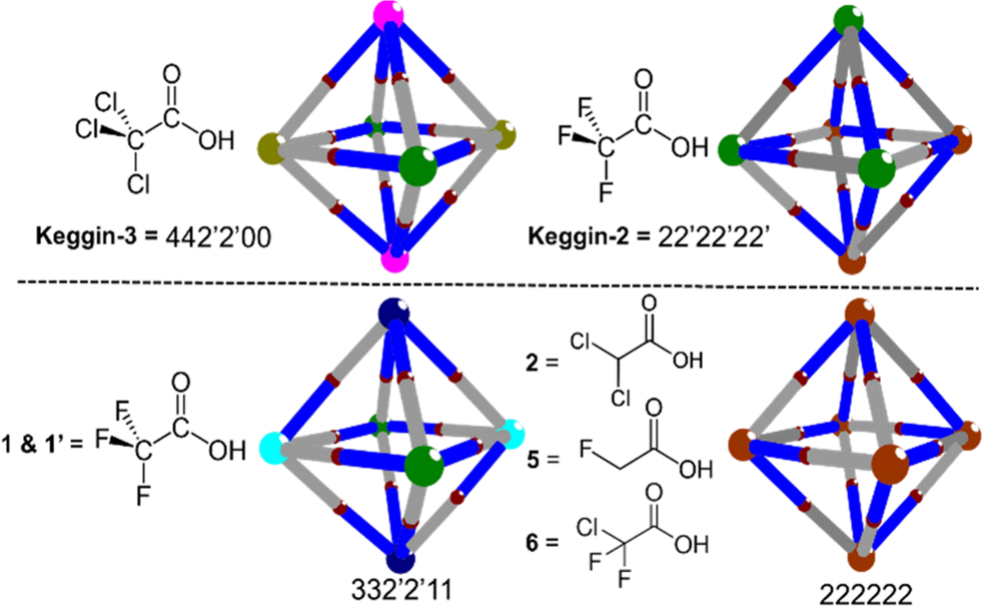
3D-representation of the supramolecular octahedron formed in {Bi_6_Fe_13_L_12_} structures. Ligands L bridge the blue-colored Bi···Fe half edges. Colors: BiL_4_, pink, 4; BiL_3_, navy, 3; BiL_2_(cis), brown, 2; BiL_2_(trans), green, 2′; BiL_1_, cyan, 1; BiL_0_, yellowish green, 0; Fe, deep red.

3D-projections of {Bi_6_Fe_13_L_12_} structures were constructed to visualize the ligand connectivity with the discrete bivertices, in order to identify the isomers and examine the differences in ligand positions; see Figure 3. Since the ligands are distributed unsymmetrically on the 12 edges of the octahedron, we denote blue bonds as carboxylate bridged edge halves and gray as nonbridged halves. The six vertices of the octahedron can have up to six different coordination modes depicted by different colors. These projections reveal that the remarkable structural feature identified from investigation of the {Bi_6_Fe_13_L_12_} cluster is the variability of ligand bridging positions on the 12 edges of the octahedron.

**Figure 4:**
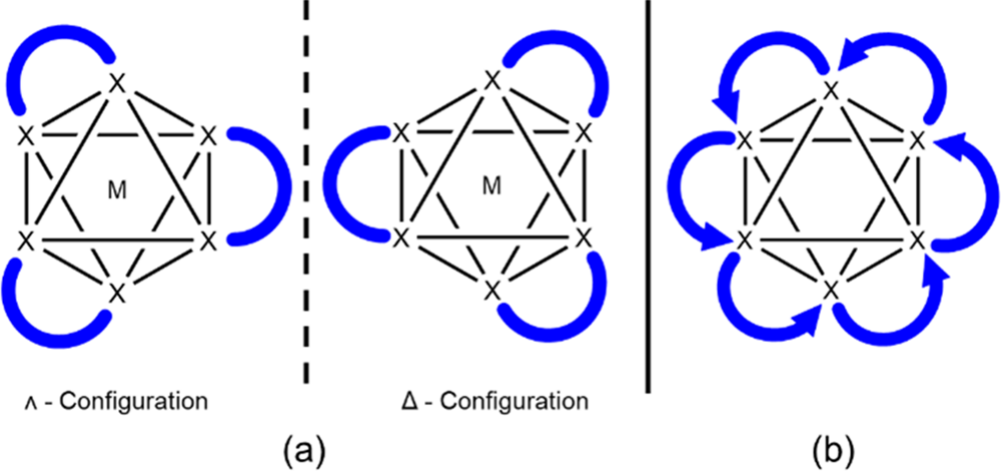
Comparison of (a) stereoisomers of Werner’s complexes of [ML_3_]^n±^ (L = X–X, i.e. bidentate ligand). (b) Example of isomers of an octahedral framework with unsymmetrical ligations indicated by directional arrows on each edge: only 6 of 12 arrows are shown for clarity.

**Figure 5:**
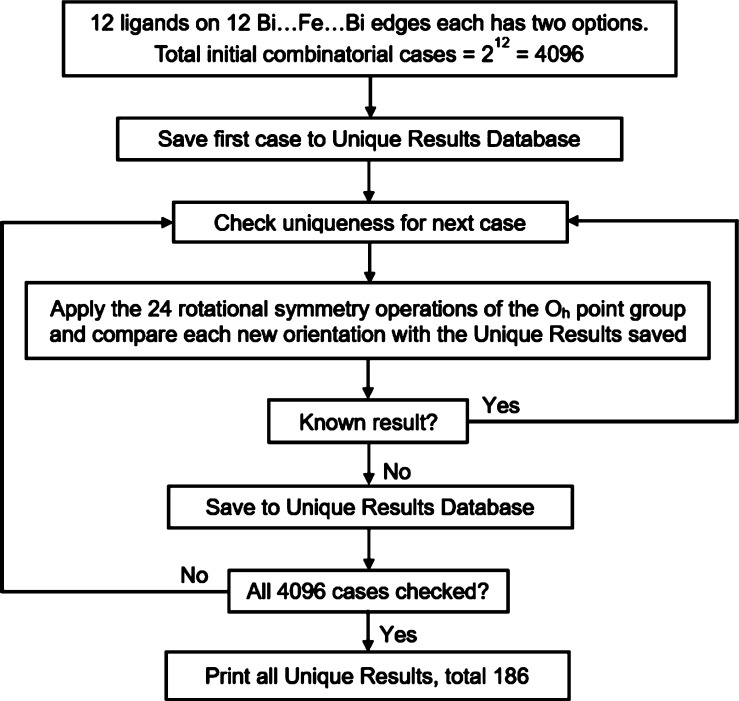
Flow diagram of Enumeration Method A.

**Figure 6:**
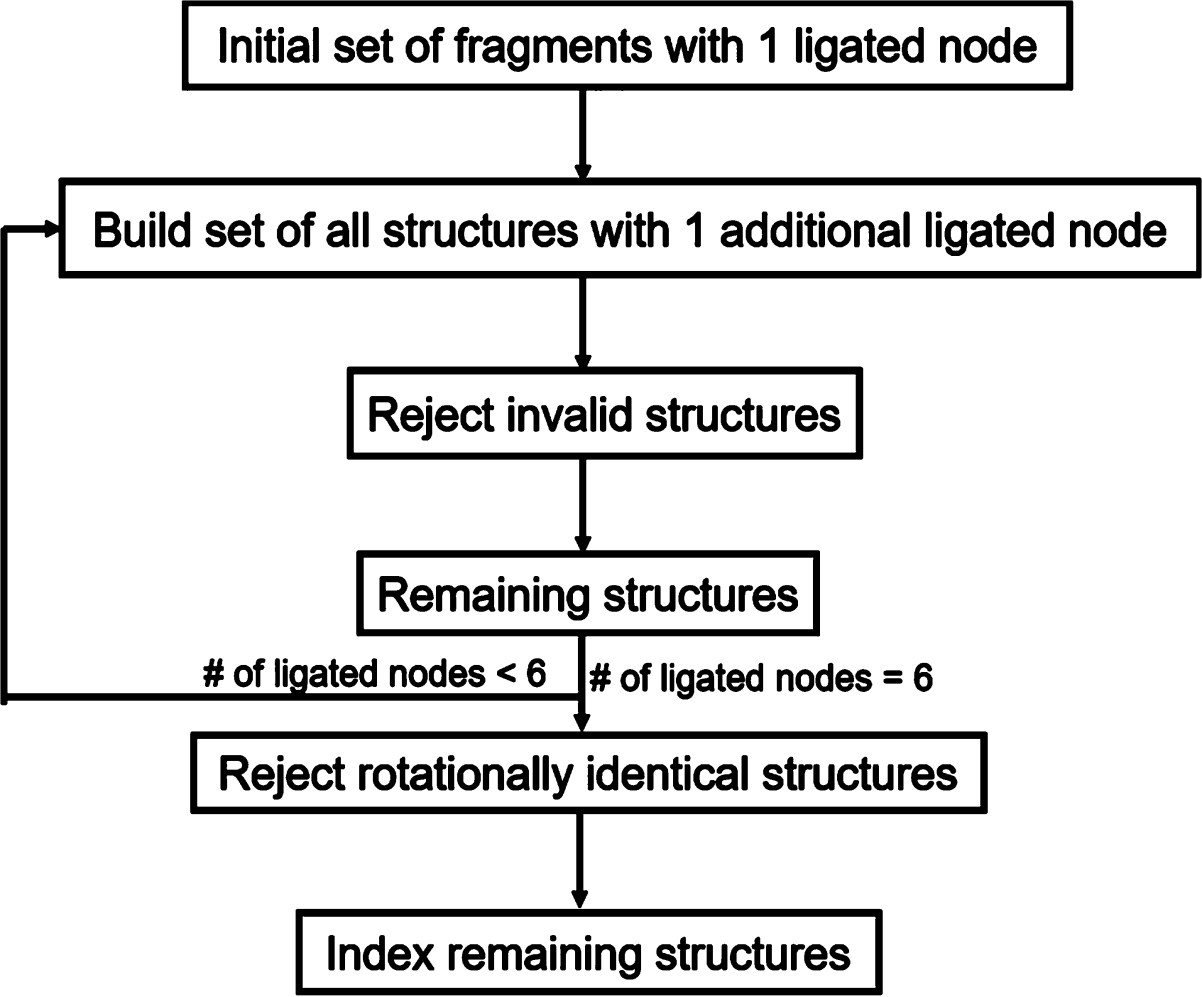
Flow diagram of Enumeration Method B.

The observation of these “supramolecular octahedra” thus provided the opportunity to compare isomerization with Werner’s classical monometallic octahedral complexes (Figure 4). While Werner’s octahedral complexes produce only two stereoisomers (Figure 4a), our supramolecular octahedron has numerous accessible isomers. This isomerism arises as the ligation along the polyhedral edge is unsymmetrical, indicated by a directional arrow (Figure 4b). It can be envisaged from the numerous combinations of arrow orientations that there are a vast number of different configurational isomers possible. To interrogate these isomers, we developed two enumerative methodologies to determine the number of potential isomers of any supramolecular octahedron bearing nonsymmetric ligands (or symmetric ligands with unsymmetrical coordination) along the edges. The architecture, decisions for the input files, and procedures for both methods are described in Figures 5 and 6 (also Figure S2a–S2d).

For the {Bi_6_Fe_13_L_12_} octahedral system, Method A is based on the premise that each carboxylate ligand has two potential sites to be found on either of the two Bi–Fe halves of each edge, and there are 12 edges; hence, 4096 (2^12^) enumeration isomers are possible. However, owing to the high symmetry of the *O*_*h*_ point group, the number of unique configurational isomers is much lower. By applying the 24 rotational symmetry operations of the *O*_*h*_ point group and validating each orientation for equivalence with each unique isomer, a total of 186 unique configurational isomers are predicted (Figures 5 and S2a). Further, the isomers can be subcategorized into 74 pairs of enantiomers identified by reflection symmetry, and the remaining 38 structures are achiral (Figure S2b).

Alternatively, Method B (Figures 6 and S2c) describes the stepwise construction of an octahedron based on the presence of six M-ions at each of the vertices. The polygon is built one vertex at a time, by allowing only edges containing a single ligand (denoted “valid”, Figure S2d). Each vertex can have a maximum of 16 different ligand configurations, but only those which yield valid edges between M_n_ and M_n+1_ allow for the construction algorithm to proceed. When a valid edge is found, the model continues to build the octahedron by adding a further M_n+2_ ions. When the structure is complete, it is compared with the others already enumerated and is thus classified as unique or nonunique.

These two enumerative methodologies demonstrate two different strategies in finding the configuration isomers. Both are applicable and compute efficiently once programmed. Each method unambiguously provided 186 configurational isomers for the octahedral model system (see Figure S3a).

To identify and name each of the 186 isomers, we developed a simple code system for the isomers where each of the six vertices of the polyhedron is labeled according to the number of ligands (or equivalent ligand donor sites) appended (Figure 3). To ensure common assignment methods, we set the following rules: (1) Nodes have labels *abcdef*, with *ab*, *cd*, and *ef* representing three pairs of trans vertices of the octahedron; (2) these pairs are arranged in descending order where *a* is the largest coordination number; (3) in the case of ML_2_ vertices, 2 (*cis*) precedes 2′ (*trans*). It should be mentioned that isomers may share the same code, e.g., enantiomers.

Using our assignment method **Keggin-3** can be denoted as 442′2′00 (symmetry point group *D*_2*h*_, Figures 3 and S5a) or 42′3300 (*C*_2*v*_, Figure S1) for the two disordered parts. In contrast, **Keggin-2** (L = CF_3_CO_2_^–^) has only two different types of Bi vertices where each is bonded with two acetate ligands in either *cis* mode (denoted as 2, brown) or *trans* mode (denoted as 2′, green) yielding notation 22′22′22′ (*C*_3_, Figure S5b). Trifluoroacetate ligated **1** and **1′** can be denoted 332′2′11 structures (*C*_2*h*_, Figures 3 and S5c) while the lower symmetry and sterically less strained dihalogen substituted acetates (CHCl_2_CO_2_^–^) and monohalogen substituted acetate (CH_2_FCO_2_^–^) derivatives **2** and **5** form the highly symmetrical 222222 isomer (*S*_6_, Figures 3 and S5d). Similarly, the unsymmetrical trihalogen substituted acetate (CClF_2_CO_2_^–^) product **6** also formed the 222222 isomers. Extensive disorders at the ligand positions of compounds **3** (CHF_2_CO_2_^–^) and **4** (CH_2_ClCO_2_^–^) meant their isomers could not be definitively assigned; however, three isomer types of 333300 (*C*_4*h*_), 22222′2′ (*S*_4_), and 441111 (*C*_4*h*_) (Figure S1) based on reasonable resolutions of the disordered parts seem possible. Considering the point groups of each of these isomers, it seems that a preliminary trend among these structures can be tentatively suggested: more symmetrically substituted ligands appear to yield less symmetric isomers (such as *C*_2*v*_, *C*_3_, and *C*_2*h*_) while less symmetric ligands **2–6a** yield those with higher-order symmetry (i.e., *S*_6_) with a similar trend in crystallographic lattices for these clusters. Note, however, that the ligand is not the only factor we believe contributes to isomer selectivity with reaction and crystallization conditions also contributing to the variety we and others have observed.

Realizing our enumeration methods to be more widely applicable than these clusters alone, we anticipated the potential of this methodology to generate insights in other systems such as MOP coordination cages and extended MOF structures. For example, an {M_6_L_12_} octahedral coordination cage, when the bridging ligand L is nonsymmetric by virtue of the two “ends” being nonidentical (i.e., ambidentate), has the same number of configurational isomers as above. Therefore, theoretically, this enumeration can be applied to several other coordination cages: polygons; platonic solids including tetrahedra, octahedra, and cubes; Archimedean solids such as truncated tetrahedra or cuboctahedra; and prism topologies such as trigonal prisms, square prisms, etc. Simple polygon assemblies including triangular and square-based supramolecular systems produce two and four isomers, respectively.^22−24^ We have also applied our enumeration methods to tetrahedral M_4_L_6_ and cubic M_8_L_12_ topologies. The former affords 8 chiral isomers (Figure S3b), whereas the latter shows a total of 186 isomers (Figure S3c), the same number as for the octahedral system. This is because both cubic and octahedral systems are symmetrically equivalent objects.

### Stability and Reactivity

Electrospray ionization mass spectrometry (ESI) on polyoxometalates has been proven as a versatile technique to understand the complexity of metal oxide’s behavior in the gas phase.^25^ Pairing of ion mobility (IM) with mass spectrometry (MS) not only allows the separation of isobaric ions (same *m*/*z*)^26^ but also provides structural information, both of which assist in our understanding of molecular aggregation and supramolecular interactions (with counter cations, protons, and solvents) of larger multicharged metal oxides.^27,28^ In addition, IM-MS offers the potential to determine the size of clusters and aggregations through collison cross-section (CCS) measurements. Over the past decade, it has been possible to explore the CCS of a variety of complex and gigantic metal oxide clusters.^29−31^ This is remarkable because the information gained from CCS measurements can be used to elucidate the self-assembly and aggregation behavior of the clusters, providing insights into their functional behavior and formation.

**Figure 7:**
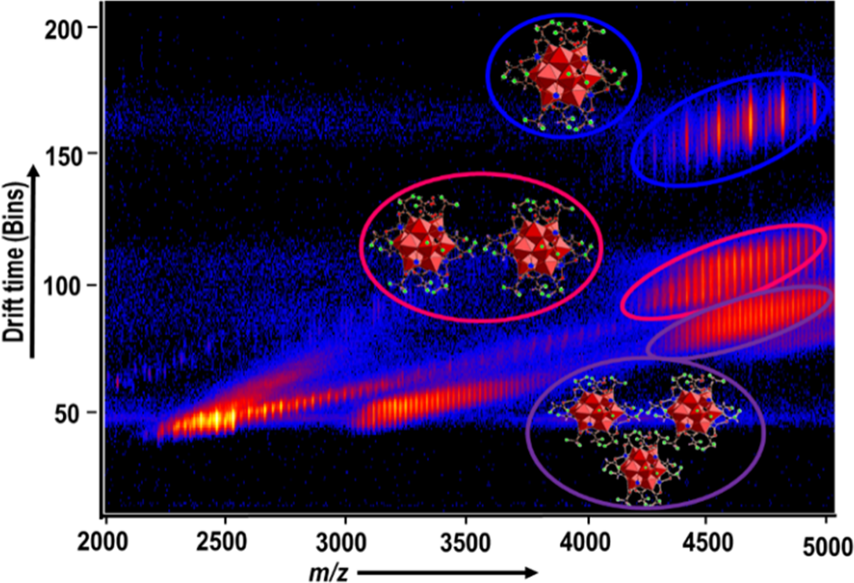
IM-MS (+ve ion mode) spectrum of compound **6a** in CH_3_CN. Peaks circled in different colors can be assigned as follows: monomeric clusters, blue; dimers of {Bi_6_Fe_13_}, magenta; trimers of {Bi_6_Fe_13_}, violet.

Compounds **1**–**3**, **5**, **6**, and **6a** showed the series of individual peaks within the region between *m*/*z* 3000 to 5000 of the IM-MS spectrum, which corresponds to {Bi_6_Fe_13_} clusters with the different number of charges, ligands, and coordinating solvents, implying molecular oligomerization. An example of the resulting IM-MS (+ve mode) of **6a** can be seen in Figure 7: a series of peaks covering a range of fragments (of charge +1 to +3) corresponds to the core Bi_6_Fe_13_O_16_(OH)_12_L_12_ structure, with the interaction between the individual monomers and alkali metal-stabilized carboxylate ligands, resulting in the formation of a unique class of clusters with the formula [{Bi_6_Fe_13_O_16_(OH)_12_(L)_12_}_*x*_(CsL)_*y*_(HL)_*z*_]^n+^, where L = CClF_2_CO_2_^–^, n = 1–3). The IM-MS spectra and complete peak assignments of compounds **1**–**6** and **6a** are shown in the Supporting Information (Table S5a–S5g).

**Figure 8:**
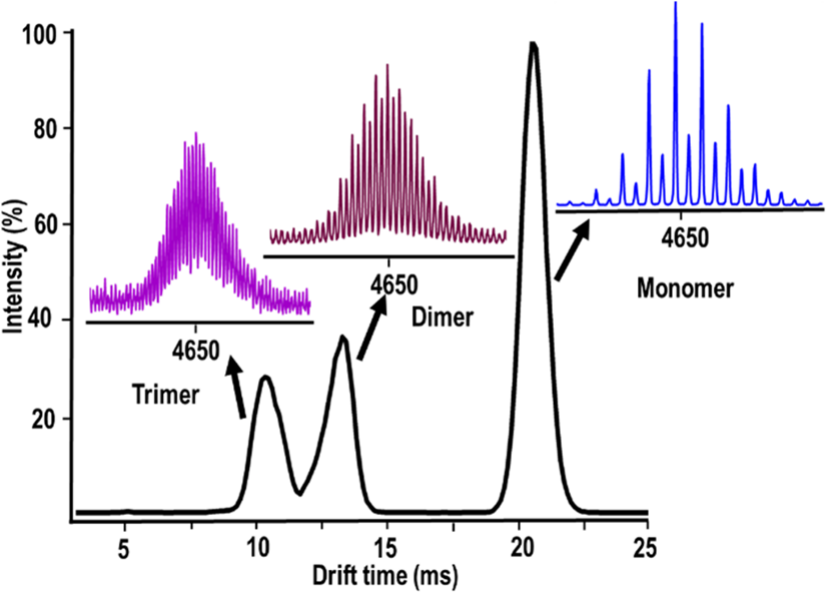
Drift time vs intensity of compound **6a** in IM-MS. Color codes as follows: monomeric {Bi_6_Fe_13_}, blue; dimers of {Bi_6_Fe_13_}, cyan; trimers of {Bi_6_Fe_13_}, pink.

Using NaI as CCS calibrant, we measured the CCS of Bi–Fe oxo clusters. Note, we chose compound **6a**, compared to the rest of the compounds, because the ligand associated within this complex has very low p*K*_a_ (Table S1) and thus its equilibrium lies far toward the free acetate, as well as the fact that **6a** has well-defined crystal structure. The drift time of different [{Bi_6_Fe_13_O_16_(OH)_12_(L)_12_}_*x*_(CsL)_*y*_(HL)_*z*_]^n+^, where L = CClF_2_CO_2_^−^, n, x, and z = 1–3, y = 2–6, based fragments with similar *m*/*z* (∼4649.0), was observed to confirm that each of these three major spots are single species (Figure 8), and thus, the CCS of these aggregates was calculated (Table 1). As predicted by its assignment, the CCS value for the dimer was double that of the monomer with the trimer CCS around three times that of the isolated cluster.

**Table 1:** Drift Time and CCS Values of Selected Species of Compound **6a**

*m/z*_*Obs*_	z	Drift time (t_D_, ms)	*m/z*_*cal*_	Peak Composition^a^	^TW^CCS_N2_ (Ω, A^2^)
4648.59	1	20.7	4648.13	[{Cluster}(CsL)_2_(HL)]^1+^	388.44
4649.95	2	13.4	4648.13	[{Cluster}_2_(CsL)_4_(HL)_2_]^2+^	775.71
4649.65	3	10.4	4648.70	[{Cluster}_3_(CsL)_6_(HL)_3_]^3+^	1162.98

a {Cluster} = {Bi_6_Fe_13_O_16_(OH)_12_(L)_12_}; L = CClF_2_CO_2_^–^.

For all the compounds examined in MeCN solvent, we observed monomeric, dimeric, and trimeric arrangements of clusters. While each complex/isomer demonstrated a preference for one type of aggregation over another (as defined by relative ion intensity, Figure S6i), no clear trend of the effect of ligand or isomer type on aggregation was observed.

## Conclusions

In conclusion, we have developed two distinct enumerative combinatorial methods which identify the configurational isomers of supramolecular metal–organic polyhedra (MOPs) bearing unsymmetrical edge coordination, exemplified by our analysis of octahedron-shaped Bi_6_Fe_13_L_12_ metal-oxides. This method can be utilized to enumerate the potential isomers of other supramolecular cages bearing unsymmetrical ligation of extended structures such as MOFs. Through our synthetic methodology, utilizing partially substituted halo-acetate ligands, several distinct configurational isomers of {Bi_6_Fe_13_L_12_} complexes were successfully synthesized. Counterintuitively more symmetric ligand substituents seemed to provide less symmetric isomers. These results therefore provide insights into the directed synthesis of low-symmetry supramolecular coordination complexes.

## Experimental Section

For materials, instrumentation, and general experimental procedures, see the SI.

### Synthetic Procedure

*Na*_*3*_*[Bi*_*6*_*Fe*_*13*_*O*_*16*_*(OH)*_*12*_*(CF*_*3*_*COO)*_*12*_*](CF*_*3*_*COO)*_*4*_*·xH*_*2*_*O (x = 36*, **1***; x = 40*, ***1′****)***Method A**: Bi(NO_3_)_3_·5H_2_O (250 mg, 0.515 mmol) was mixed with Fe(NO_3_)_3_·9H_2_O (832.8 mg, 2.06 mmol) in 25 mL of water with stirring, forming a dark orange solution with a white precipitate (solution A). Then, trifluoroacetic acid (3 mL) was added directly to solution A. It became a clear colorless solution. Then, the pH of the reaction mixture was increased to 2.6 using 1 M NaOH solution, and it was filtered further to remove any insoluble impurities. Then the clear dark red solution was left to crystallize (18 °C) for 3 weeks. Dark red block shaped crystals were formed and collected by gravitational filtration. Yield: 33 mg, 4.2% (based on Fe). Elemental analysis for Na_3_[Bi_6_Fe_13_O_16_(OH)_12_(CF_3_COO)_12_](CF_3_COO)_4_·36H_2_O. **1**. C_32_H_84_Bi_6_F_48_Fe_13_Na_3_O_96_ M_w_ = 4965.69, calc. (%): C 7.74, H 1.71, Na 1.39. Found (%): C 7.84, H 1.39, Na 1.40. ICP results: Bi:Fe = 6:13.1. Note: The same polymorph of compound **1** was obtained when NaOH was employed as a deprotonating agent instead of trifluoroacetate and trifluoroacetic acid buffer. **Method B:** Bi(NO_3_)_3_·5H_2_O (0.050 g, 0.103 mmol) was added to 25 mL of water with stirring, forming a white insoluble precipitate. Concentrated (70%) nitric acid was added dropwise until all the bismuth nitrate dissolved, forming a clear colorless solution. To this, Fe(NO_3_)_3_·9H_2_O (0.166 g, 0.412 mmol) was added, forming a clear solution. Then Pb(NO_3_)_2_ (0.034 mg, 0.103 mmol) was added. Further, trifluoroacetic acid (0.41 mL, 5.36 mmol) was added to it. Then, the pH of the reaction mixture was increased to 2.6 using 2 M Na_2_CO_3_ solution and heated for 60 min at 55–60 °C. After cooling, the clear dark orange solution was left to crystallize (18 °C) for 3 weeks. Dark red block shaped crystals were formed and collected by gravitational filtration. Yield: 29 mg, 18.48% (based on Fe). Na_3_[Bi_6_Fe_13_O_16_(OH)_12_(CF_3_COO)_12_](CF_3_COO)_4_·40H_2_O; **1′**. C_32_H_92_Bi_6_F_48_Fe_13_Na_3_O_100_ M_w_ = 5037.75, calc. (%): C 7.63, H 1.84, Na 1.37. Found (%): C 7.59, H 1.05, Na 1.27. ICP results: Bi:Fe = 6:11.7.

#### Na_2_[Bi_6_Fe_13_O_16_(OH)_12_(CHCl_2_COO)_12_](CHCl_2_COO)_3_·31H_2_O (**2**)

Bi(NO_3_)_3_·5H_2_O (0.100 g, 0.206 mmol) was added to 25 mL of water with stirring forming a white insoluble precipitate. Concentrated nitric acid (70%) was added dropwise until all the bismuth nitrate dissolved, forming a clear colorless solution. To this, Fe(NO_3_)_3_·9H_2_O (0.166 g, 0.412 mmol) was added, forming a clear solution. Then sodium dichloroacetate (0.808 g, 5.36 mmol) was added, forming a clear pale-yellow solution. The pH of the reaction mixture was increased to 2.6 using 2 M Na_2_CO_3_ solution and heated for 90 min at 55–60 °C. After cooling, the orange filtrate was separated from the sandy precipitate by centrifugation and left to crystallize (18 °C) for 2 days. Dark red block shaped crystals were formed and collected by gravitational filtration. Yield: 34.7 mg. 22.2% (based on Fe). Elemental analysis for C_30_H_89_Bi_6_Cl_30_Fe_13_Na_2_O_89_ M_w_ = 4963.41, calc. (%): C 7.26, H 1.81, Na 0.93. Found (%): C 7.46, H 1.22, Na 0.85. ICP results: Bi:Fe = 6:12.6.

#### Na[Bi_6_Fe_13_O_16_(OH)_12_(CHF_2_COO)_12_](CHF_2_COO)_2_·12H_2_O (**3**)

100 μL of 1 M Fe(NO_3_)_3_·9H_2_O (0.1 mmol) solution and 1000 μL of 0.1 M of Bi(NO_3_)_3_·5H_2_O (0.1 mmol) were mixed to give a colorless solution. 164 μL (2.6 mmol) of difluoroacetic acid was added. A slightly orange solution was formed. Then, the pH of the solution was increased to 2.6 using 0.2 molar Na_2_CO_3_. The clear reddish orange solution which formed was left for crystallization (18 °C). Reddish orange crystals were formed after 3–4 days. Crystals were collected carefully from the mother liquor and used for microanalysis. Yield: 15.3 mg, 50.2% (based on Fe). Elemental analysis for C_28_H_50_Bi_6_F_28_Fe_13_NaO_68_, M_w_ = 4009.46, calc. (%): C 8.39, H 1.26, Na 0.57; found (%): C 9.24, H 1.25, Na 0.11; ICP results: Bi:Fe = 6:12.6.

#### Na_2_[Bi_6_Fe_13_O_16_(OH)_12_(CH_2_ClCOO)_12_](CH_2_ClCOO)_3_·15H_2_O (**4**)

Bi(NO_3_)_3_·5H_2_O (0.100 g, 0.206 mmol) was added to 25 mL of water with stirring, forming a white insoluble precipitate. Concentrated nitric acid (70%) was added dropwise until all the bismuth nitrate dissolved, forming a clear colorless solution. To this, Fe(NO_3_)_3_·9H_2_O (0.166 g, 0.412 mmol) was added, forming a clear solution. Subsequently, chloroacetic acid (0.506 g, 5.356 mmol) was added, forming a clear pale-yellow solution. The pH of the reaction mixture was increased to 2.52 using 2 M Na_2_CO_3_ solution. After cooling, the orange filtrate was separated from the sandy precipitate by centrifugation and left to crystallize (18 °C) for 2 days. Dark red block shaped crystals were formed and collected by gravitational filtration; then the filtrate was kept for further crystallization. Red crystals were formed after 2 weeks. Yield: 32.1 mg, 24.4% (based on Fe). Elemental analysis for C_30_H_72_Bi_6_Cl_15_Fe_13_Na_2_O_73_. M_w_ = 4158.5, calc. (%): C 8.66, H 1.74, Na 1.10. Found (%): C 10.03, H 1.11, Na 0.48. ICP results: Bi:Fe = 6:14.2.

#### Na[Bi_6_Fe_13_O_16_(OH)_12_(CH_2_FCOO)_12_](NO_3_)_2_·23H_2_O (**5**)

25 μL of 1 M Fe(NO_3_)_3_·9H_2_O (0.025 mmol) solution and 250 μL of 0.1 M of Bi(NO_3_)_3_·5H_2_O (0.025 mmol) were mixed together to give a colorless solution. 50 μL of fluoroacetic acid (34.8 mmol) was added, with stirring, and a slightly orange solution formed. Then, the pH of the solution was increased to 3.0 using 0.2 molar Na_2_CO_3_. A clear reddish orange solution formed and was left to crystallize (18 °C). Reddish orange crystals were formed after 3–4 days. Crystals were collected carefully from the mother liquor and used for microanalysis. Yield: 4.5 mg, 59.8% (based on Fe). Elemental analysis for C_24_H_82_Bi_6_F_12_Fe_13_N_2_NaO_81_ M_w_ = 3925.71, calc. (%): C 7.34, H 2.11, N 0.71, Na 0.58; found (%): C 7.36, H 1.74, N 0.71, Na 0.13. ICP results: Bi:Fe = 6:12.9.

#### Na_4_[Bi_6_Fe_13_O_16_(OH)_12_(CClF_2_COO)_12_](CClF_2_COO)_5_·3CH_3_CN·15H_2_O (**6**)

0.5 mL of 1 M Fe(NO_3_)_3_·9H_2_O (0.5 mmol) solution and 5 mL of 0.1 M of Bi(NO_3_)_3_·5H_2_O (0.5 mmol) were mixed. 1.5 mL (18 mmol) of chlorodifluoroacetic acid was added. The pH of the solution was increased to 1.5 using 2 M Na_2_CO_3_ solution. At this stage, a white cloudy solution was formed. Then, the pH of the solution was increased to 2.6 using 2 M Na_2_CO_3_ solution and allowed to stir at room temperature for 30 min. A cloudy orange solution remained and was centrifuged further to give a clear orange solution. Then, it was left to crystallize (18 °C). Red cubic shaped crystals were formed after 2 weeks. Crystals were collected carefully from the mother liquor and recrystallized from CH_3_CN and further used for microanalysis. Yield: 109 mg, 55% (based on Fe). Elemental analysis for C_40_H_51_Bi_6_Cl_17_F_34_Fe_13_N_3_Na_4_O_77_ M_w_ = 5126.28, calc. (%): C 9.37, H 1.00, N 0.82; found (%): C 7.84, H 1.05, N 0.30. ICP results: Bi:Fe = 6:12.2. Note: Low nitrogen due to loss of some of CH_3_CN while drying the sample in air.

#### Cs_4_[Bi_6_Fe_13_O_16_(OH)_12_(CClF_2_COO)_12_](CClF_2_COO)_5_·3CH_3_CN·10H_2_O (**6a**)

0.5 mL of 1 M Fe(NO_3_)_3_·9H_2_O (0.5 mmol) solution and 5 mL of 0.1 M of Bi(NO_3_)_3_·5H_2_O (0.5 mmol) were mixed. 1.5 mL (18 mmol) of chlorodifluoroacetic acid was added. The pH of the solution was increased to 1.5 using 1 M Cs_2_CO_3_ solution. At this stage, a white cloudy solution was formed. Then, the pH of the solution was increased to 2.6 using 1 M Cs_2_CO_3_ solution and allowed to stir at room temperature for 30 min. A clear orange solution was formed and was left to crystallize (18 °C). Red cubic shaped crystals were formed after a week. Crystals were collected carefully from the mother liquor and recrystallized from CH_3_CN and further used for microanalysis. Yield: 105 mg, 49.8% (based on Fe). Elemental analysis for C_40_H_41_Bi_6_Cl_17_Cs_4_F_34_Fe_13_N_3_O_72_ M_w_ = 5475.88, calc. (%): C 8.77, H 0.75, N 0.77; found (%): C 8.42, H 0.78, N 0.61. ICP results: Bi:Fe = 6:12.2.

## Supporting Information Available

Characterization of compounds, including X-ray diffraction data, IR and mass spectra, and TGA (PDF)

## Accession Codes

CCDC 2074494–2074501 contain the supplementary crystallographic data for this paper. These data can be obtained free of charge via www.ccdc.cam.ac.uk/data_request/cif, or by emailing data_request@ccdc.cam.ac.uk, or by contacting The Cambridge Crystallographic Data Centre, 12 Union Road, Cambridge CB2 1EZ, UK; fax: +44 1223 336033.
