# Supplementary material for: Exploring the Geometric Space of Metal–Organic Polyhedrons (MOPs) of Metal-Oxo Clusters
Source: Inorg Chem. 2021 Sep 22;60(19):14772–8. doi: 10.1021/acs.inorgchem.1c01987 (PMC8493551; doi:10.1021/acs.inorgchem.1c01987)
Supplement: Supplementary file 1 — ic1c01987_si_001.pdf [file ic1c01987_si_001.pdf]

Exploring the Geometric Space of Metal-Organic Polyhedrons (MOPs) of Metal-Oxo Clusters

Balamurugan Kandasamy, Edward Lee, De-Liang Long,* Nicola Bell and Leroy Cronin*
School of Chemistry, The University of Glasgow, Glasgow G12 8QQ (UK)
*email: deliang.long@glasgow.ac.uk; lee.cronin@glasgow.ac.uk

Table of Contents

1. Materials and Instrumentations
2. Synthesis
3. Enumeration Methods of Configurational Isomers
4. Crystallographic Data
5. Bond Valence Sum (BVS) Studies
6. IM-MS Measurements
7. Collison Cross Section (CCS) Studies of 6a
8. Infrared Spectroscopy (IR)
9. Thermogravimetric Analysis (TGA)
10. References

1. Materials and Instrumentations

All reactions and manipulations were carried out in open air atmosphere. Iron (III) nitrate nonahydrate and dichloroacetic acid were purchased from Fluka chemicals Ltd. Difluoroacetic acid, chlorodifluoro acetic acid and were purchased from Fluorochem Ltd. Bismuth (III) nitrate penta hydrate, fluoroacetic acid, chloroacetic acid, sodium carbonate and cesium carbonate were purchased from Sigma Aldrich chemicals. All reagents were purchased and used as received.

Elemental Analyses: Element analyses for Bi, Fe, Na and Cs were performed on a Leeman inductivity-coupled plasma (ICP) spectrometer while carbon, nitrogen and hydrogen content were determined by the microanalysis services within the School of Chemistry, University of Glasgow using an EA 1110 CHNS, CE-440 Elemental Analyzer.

Single Crystal X-Ray Diffraction: A suitable single crystal was selected and mounted onto a rubber loop using Fomblin oil. Single-crystal datasets and unit cells for compound **1-6**, **1'** and **6a** were collected at 150(2) K on a Bruker Apex II Quasar diffractometer equipped with a graphite monochromator (λ (MoKα) = 0.71073 Å) of a microfocus X-ray source (50 kV, 1.0 mA) or a Rigaku XtaLAB Synergy R diffractometer equipped with a graphite monochromator ($\lambda_{Mo\text{-}K\alpha}$ = 0.71073 Å) of a micro-focus sealed X-ray source (50 kV, 24.0 mA). Data collection and reduction were performed using the Apex3 or CrysAlisPro software package and structure solution and refinement were carried out with SHELXS-97 or later versions and SHELXL-2018/3[1] via WinGX.[2] Most of the non-hydrogen atoms (including those disordered) were anisotropically refined. Corrections for incident and diffracted beam absorption effects were applied using analytical numeric absorption correction[3] on multifaceted crystal models.

IM-MS Measurements: Samples were prepared by dissolving pure crystals of **1-6** and **6a** in HPLC grade polar organic solvents such as acetonitrile (CH_3CN) and methanol (MeOH), at approximately 10 mg in 0.5 ml CH_3CN for **1** and **2,** 20 mg in 1ml of CH_3CN for **3**, 5 mg in 0.5 ml MeOH for **4** and **5,** 40 mg in 1ml CH_3CN for **6** and **6a**; these solutions were filtered, if it is cloudy, and analyzed with no further purification. Spectra were acquired on a Waters Synapt G2 HDMS instrument in Sensitivity mode (except where otherwise stated), with samples infused into the standard ESI source at 5 µL/min using a Harvard syringe pump. The following parameters were used for acquisition of all spectra (unless otherwise stated): ESI capillary voltage, 2.7 kV; sample cone voltage, 35 V; extraction cone voltage, 4.0 V; source temperature, 80 °C; desolvation temperature, 180 °C; cone gas (N_2) flow, 15 L/h; desolvation gas (N_2) flow, 750 L/h; source gas flow, 0 mL/min; trap gas flow, 2 mL/min; helium cell gas flow, 180 mL/min; IMS gas flow, 90

mL/min; IMS DC entrance, 25.0; helium cell DC, 35.0; helium exit, −5; IMS bias, 3.0; IMS DC exit, 0; IMS wave velocity, 1000 m/s; IMS wave height, 40 V. Data were acquired using MassLynx v4.1 and initially processed using DriftScope v2.2. IMS-MS spectra are displayed with a linear intensity scale using the color-coding shown in the accompanying key; no filtering is applied to limit signals (e.g., no filtering of signals <5% in 2D map; that is, few other signals are visible in the raw data with no manipulation). To determine drift times (tD) of species of interest in the IMS cell arrival time distribution (ATD), data were extracted from Driftscope/Masslynx, and fit to Gaussian curves using Fityk v0.9.8 to determine a representative retention times peak center.

pH Measurements: Measurements were taken on a Hanna Instruments HI 9025C microcomputer pH meter, with a BCH combination pH electrode (309-1065) and HI 7669/2W temperature probe.

Infrared Spectroscopy: All samples were collected in transmission mode using an ATR fitted JASCO FT-IR-410 spectrometer. Wavenumbers are given in cm^{-1}. Intensities are denoted as w = weak, m = medium, s = strong, br = broad, sh = sharp.

Thermogravimetric Analysis: Analysis for characterization was performed on a TA Instruments Q 500 Thermogravimetric Analyzer under air flow with a heating rate of 10°C min-1 up to 1000°C. Analysis for Solution Studies were performed on TA Discovery TGA 550 where ~5 mg solid samples were loaded onto a platinum pan, the weight-temperature changes were recorded under N_2 protection.

2. Synthesis

Addition notes for synthesis: The formation of compounds **1-6** in an aqueous solution is extremely pH-sensitive, with the solution turning instantly into insoluble sandy precipitate at higher pH (> 3.5), which occurs due to olation formation of extended Fe-oxides and oxyhydroxides. The reaction mixture is deep red in color, which is directly proportional to concentration, but also white crystals regularly appeared, which was structurally characterized as $[Bi_{12}O_{10}(OH)_6(NO_3)_6]^{4+}$.[4] Also, the reaction concentration plays a vital role with higher concentration reaction mixtures (1 mM) found to yield sandy precipitate alongside a few low-quality crystals, while lower concentrations (0.05 - 0.1 mM) led to good XRD quality single crystals. It is important to mention that, for L = $CHCl_2CO_2^-$ and $CH_2ClCO_2^-$, we frequently isolated the high-quality crystals of $[Fe_3O(L)_6(H_2O)_3]^+$. While these cationic iron-oxo trimers were not obtained for L = $CF_3CO_2^-$, $CHF_2CO_2^-$, $CH_2FCO_2^-$ and $CClF_2CO_2^-$, likely due to the presence of strong F…H-O hydrogen bonding

interactions with the aqueous medium (**Table S1**). Furthermore, the same iron-oxo trimers was obtained when acetic acid (CH_3CO_2H) was employed. The effect of different alkali metal cations additionally provided insight regarding the importance of the carboxylate ligands contribution towards $[Bi_6Fe_{13}O_{16}(OH)_{12}L_{12}]^+$ cluster formation. It has been previously proposed that Bi^{3+} is required to stabilize the Fe-oxo clusters, first forming the $BiFe_3O_2(CCl_3CO_2)_8$ fragment followed by transformation to an α-Keggin type Bi-Fe-oxo clusters.[5] Note it has been reported that $BiFe_3O_2(CCl_3CO_2)_8$ was isolated only in the presence of excess sodium ions whereas in our work with potassium or cesium salts only $Fe_4O_2(CCl_3CO_2)_8$ was isolated. Thus we can conclude that the formation of $[Bi_6Fe_{13}L_{12}]$ clusters are both ligand and cation dependent. Some compounds with higher pKa ligands have considerably lower Na contents found. This can be explained by partial protonation of the solvated carboxylate ligands whereby proton partially replaces Na to balance the charger of the compound.

Table S1. pKa of the carboxylic acids† and $\{Bi_6Fe_{13}L_{12}\}$ product isomer types

Compound	Carboxylic acids	pKa [6]	Crystal shape	Product cluster isomer types	Cluster symmetry point group	Reference
**Keggin-3**	CCl_3CO_2H	0.52	-	442’2’00/42’3300	D_{2h}/C_{2v}	7
**Keggin-2**	CF_3CO_2H	0.50	-	22’22’22’	C_3	8
**1**	CF_3CO_2H	0.50	Red cube	332’2’11	C_{2h}	This work
**1’**	CF_3CO_2H	0.50	Red hexagon	332’2’11	C_{2h}	This work
**2**	$CHCl_2CO_2H$	1.26	Red cube	222222	S_6	This work
**3**	CHF_2CO_2H	1.33	Red prism	(333300/22222’2’/ 441111)§	C_{4h}/S_4	This work
**4**	CH_2ClCO_2H	2.87	Red cube	(333300/22222’2’/ 441111)§	C_{4h}/S_4	This work
**5**	CH_2FCO_2H	2.59	Red hexagon	222222	S_6	This work
**6**	$CClF_2CO_2H$	0.46	Red hexagon	222222	S_6	This work
**6a**	$CClF_2CO_2H$	0.46	Red hexagon	222222	S_6	This work

† Acetic acid pKa = 4.76 for information; § Disorder cannot be resolved but could be one of these three possibilities.

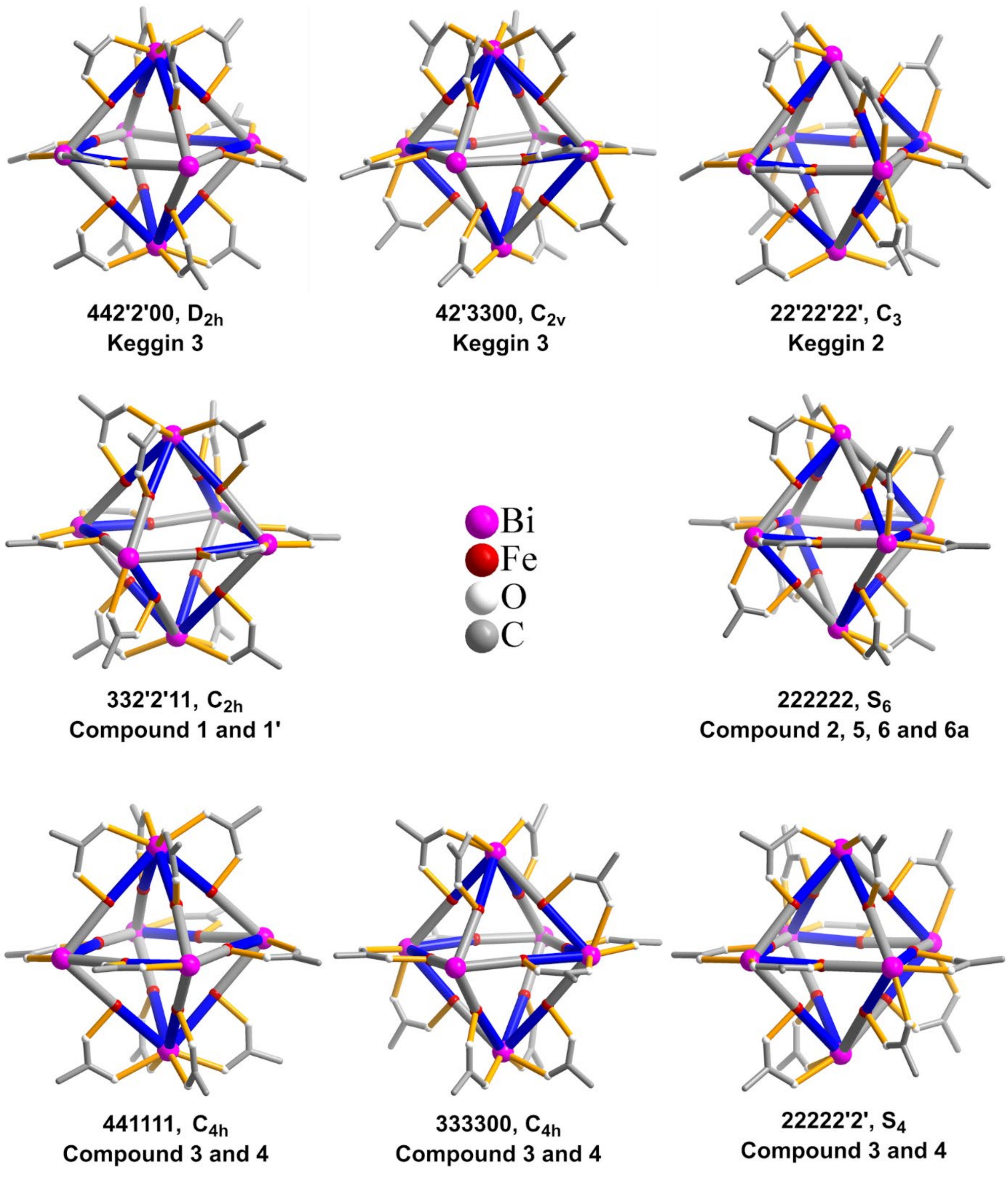

Figure S1. Isomers and their symmetry point groups of cluster $\{Bi_6Fe_{13}L_{12}\}$ listed in Table S1. Images were drawn from crystal structure data. Centre iron ion, halogen atoms, oxo, hydroxy and water ligands on cluster are omitted for clarity.

3. Enumeration Methods of Configurational Isomers

The $Bi_6Fe_{13}L_{12}$ cluster retains a typical octahedron structure, in which six Bi^{3+} atoms are located on the '6' vertices and twelve Fe^{3+} ions are positioned at the centers of the '12' Bi…Bi edges of the Bi_6 octahedron. Such unique topological arrangements of the Fe^{3+} atoms generated '24' Bi…Fe half edges equally and, accordingly, it creates super-symmetric arrangements with the coordinating acetate ligands. It can be easily visualized that each Bi…Fe half-edge can have one acetate bridging ligand but due to the coordination number restrictions on Fe in this cluster, each Fe can only have one coordinated acetate ligand attached. Therefore, the maximum ligand number for a cluster is equal to the number of twelve Fe^{3+} centers or the number of twelve Bi…Fe…Bi edges. Considering the advantage of coordinating acetate ligands on available Bi…Fe…Bi edges, it is certainly possible to enumerate the configurational isomers of $Bi_6Fe_{13}L_{12}$ oxo clusters. We herein developed two independent analytical methods to compute the number of configurational isomers. In both methods (discussed below), two important rules have been considered and these rules are then used to treat octahedral molecules. First, no edge can have two coordinated acetate ligands due to the steric effect. Second, all the possible permutational isomers have undergone all 48 O_h symmetry operations (E, $8C_3$, $6C'_2$, $6C_4$, $3C_2$, i, $6S_4$, $8S_6$, $3\sigma_h$, $6\sigma_d$). All code relating to both enumeration methods can be found at: https://github.com/croningp/polyhedral-isomerism

3.1 Enumeration Method A

Each coordinating acetate ligand bridging between Bi…Fe on a Bi…Fe…Bi edge has two possibilities and therefore 12 ligands primarily produce initial combinatorial cases $2^{12} = 4096$, and such numbers certainly cannot be valid as distinct configurational isomers because of the super-symmetric nature of octahedral skeletons, and thus unique configurational isomers are always expected to be much less than 4096. To work out the precise number of unique configurational isomers, all the 24 rotational symmetry operations of the octahedron (O_h) point group were performed on all the 4096 combinatorial cases. All the initial and resulted topographical orientations compared to eliminate the identical configurations that are overlapped. The final number of configurational isomers was found to be 186, **Figure S2a**. When the above process checking equivalent results is performed by using all 48 symmetry operations of the O_h point group, the final number of unique structures is 112. That means reflection operations have identified 186-112 = 74 pairs of racemates. So, the enantiomers in 186 unique results are 74×2 = 148. Total achiral structures are only 38 (**Figure 3a**). This can be alternatively explained by the simple flow diagram in **Figure S2b**.

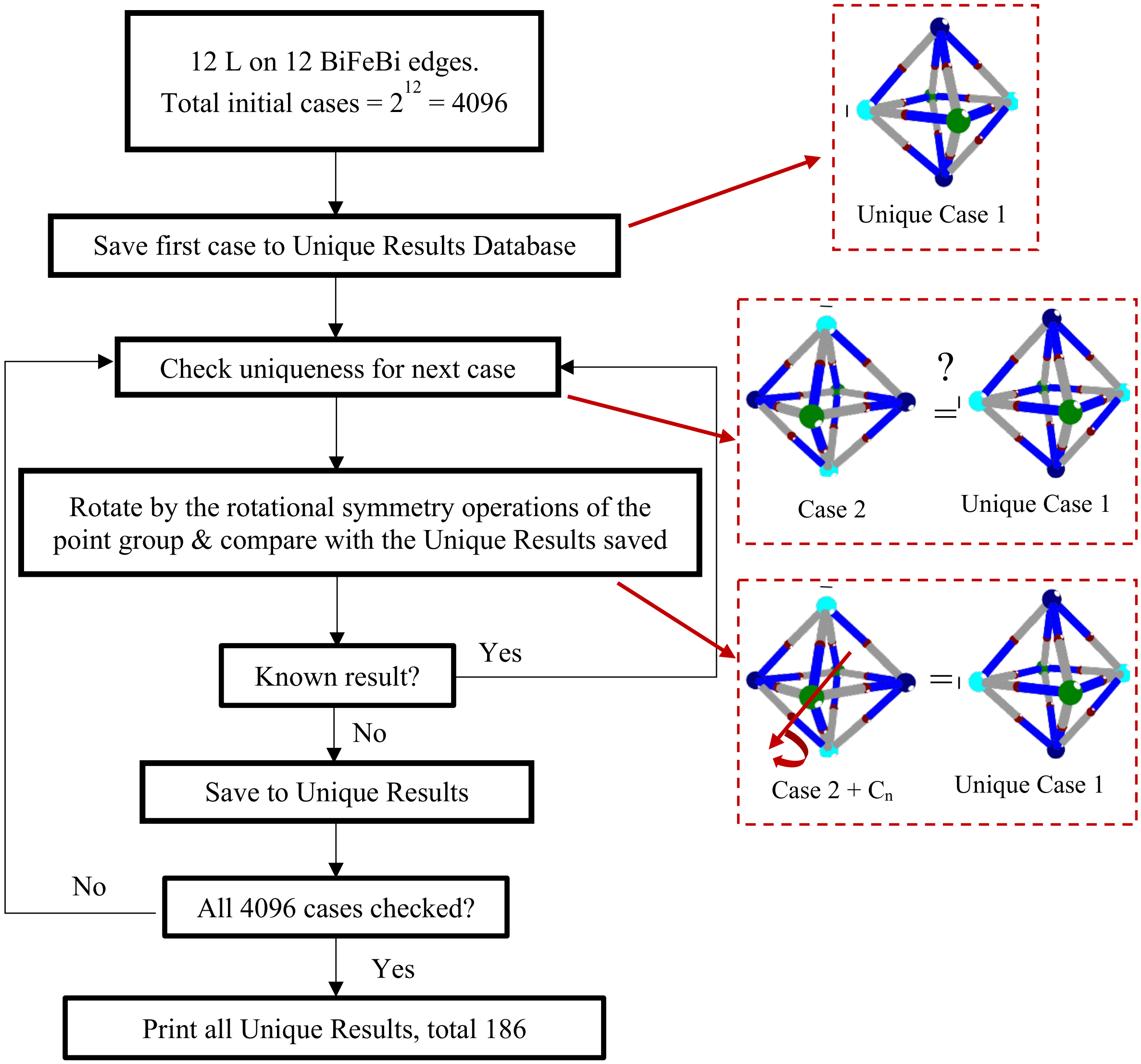

Figure S2a. Flow diagram to identify unique results in Method **A**. An example is depicted on right hand side.

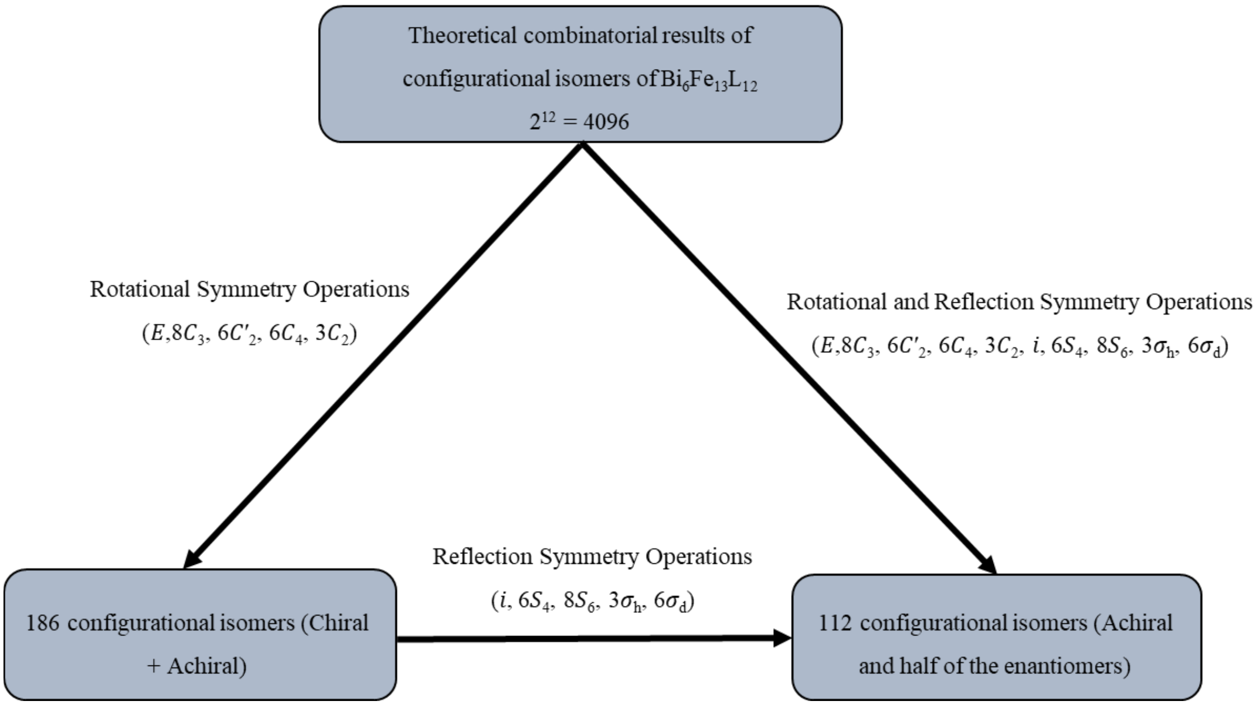

Figure S2b. Flow diagram to identify chiral results in Method **A**.

3.2. Enumeration Method B

Primarily, all the six Bi^{3+} ions were numbered as n_1-n_6, and arranged as an octahedron topology, which undoubtedly produces twelve edges. Twleve Fe^{3+} ions are located at center of each edge and equally generates 24 (12×2) edges. Considering the coordination mode of carboxylate ligand to bismuth metal centers in a such way with 0, 1, 2, 3, 4 combinations, (4 is maximum), a total 16 possible bonding configurations (m_1-m_{16}) can be identified for each Bi^{3+} centers. The 16 bonding configurations can be classified as follows: one '0' acetate coordinated Bi (m_1), four '1' acetate coordinated Bi (m_2-m_5), six '2' acetate coordinated Bi (m_6-m_{11}), four '3' acetate coordinated Bi (m_{12}-m_{15}) and one '4' acetate coordinated Bi (m_{16}). Iteration through bismuth atoms (n_1-n_6) and carboxylate ligand (m_1-m_{16}) to spot the nature of configurational isomers. As a general rule of thumb, the acetate ligand locates at the edges of the octahedron, as it happens *via* addition of second bismuth atom (n_2). The iteration begins with n_1 atom with m_1 configuration, which has no acetate ligand between atoms, and hence this configuration is invalid. Increment 'n' (let n_2) and 'm' (let m_{n+1}) to attain the next possible bond between the atoms. To validate the combination, verify whether the bond is pointing towards atom n_1. If yes, then it is valid combination and save the structure if it is unique, else the combination is invalid (**Figure S2c**). Similarly, perform the aforesaid steps on 'n_3-n_6' with 'm_3-m_{16}' configurations. In total, 186 possible configurational isomers could be identified by applying the above algorithms. Among them, 74 pairs of enantiomers and remaining 38

structures are achiral in nature (**Figure 3a**). Flow diagram with real structure examples is depicted in **Figure S2d**.

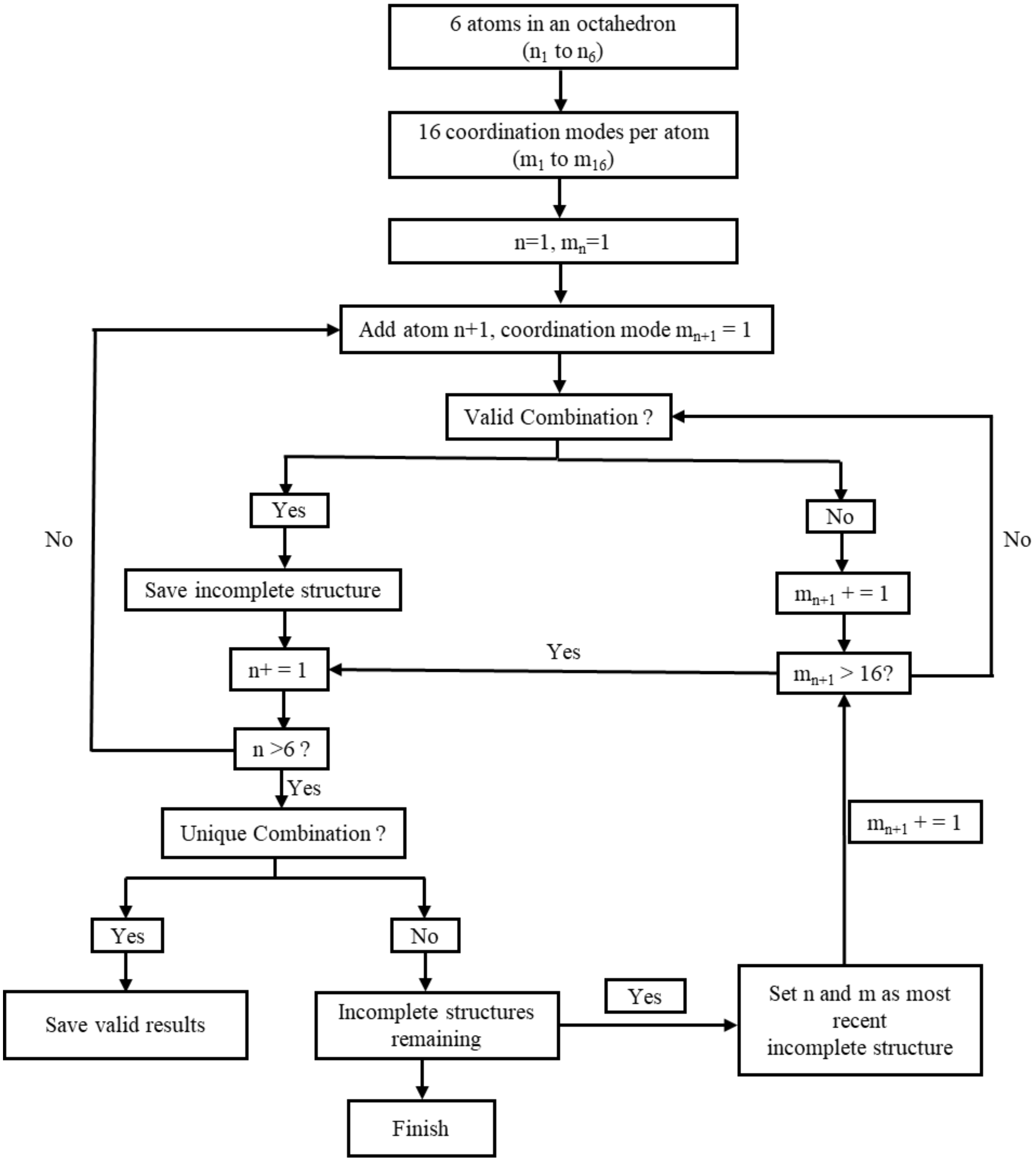

Figure S2c. Flow diagram of Enumeration Method B

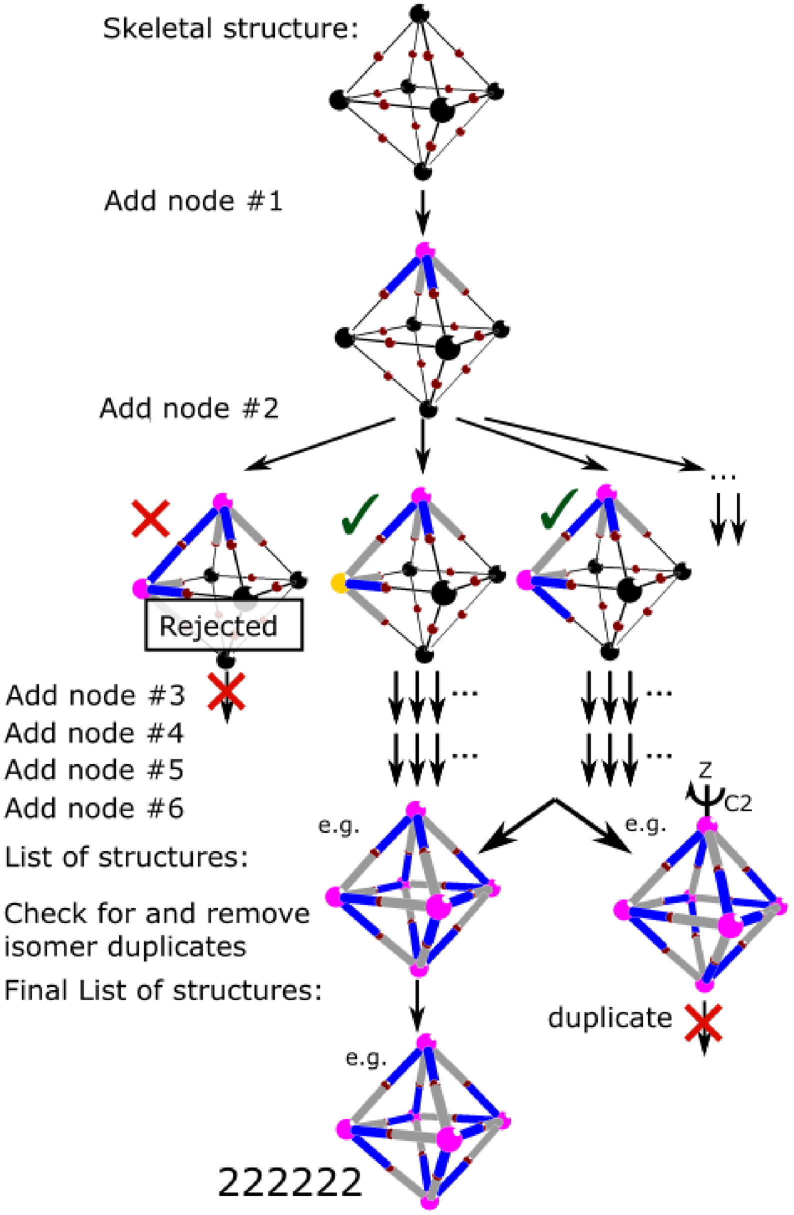

Figure S2d. Schematic representation of how enumeration method B finds unique isomers. Invalid combinations occur when an edge has more or less than one ligand (blue stick).

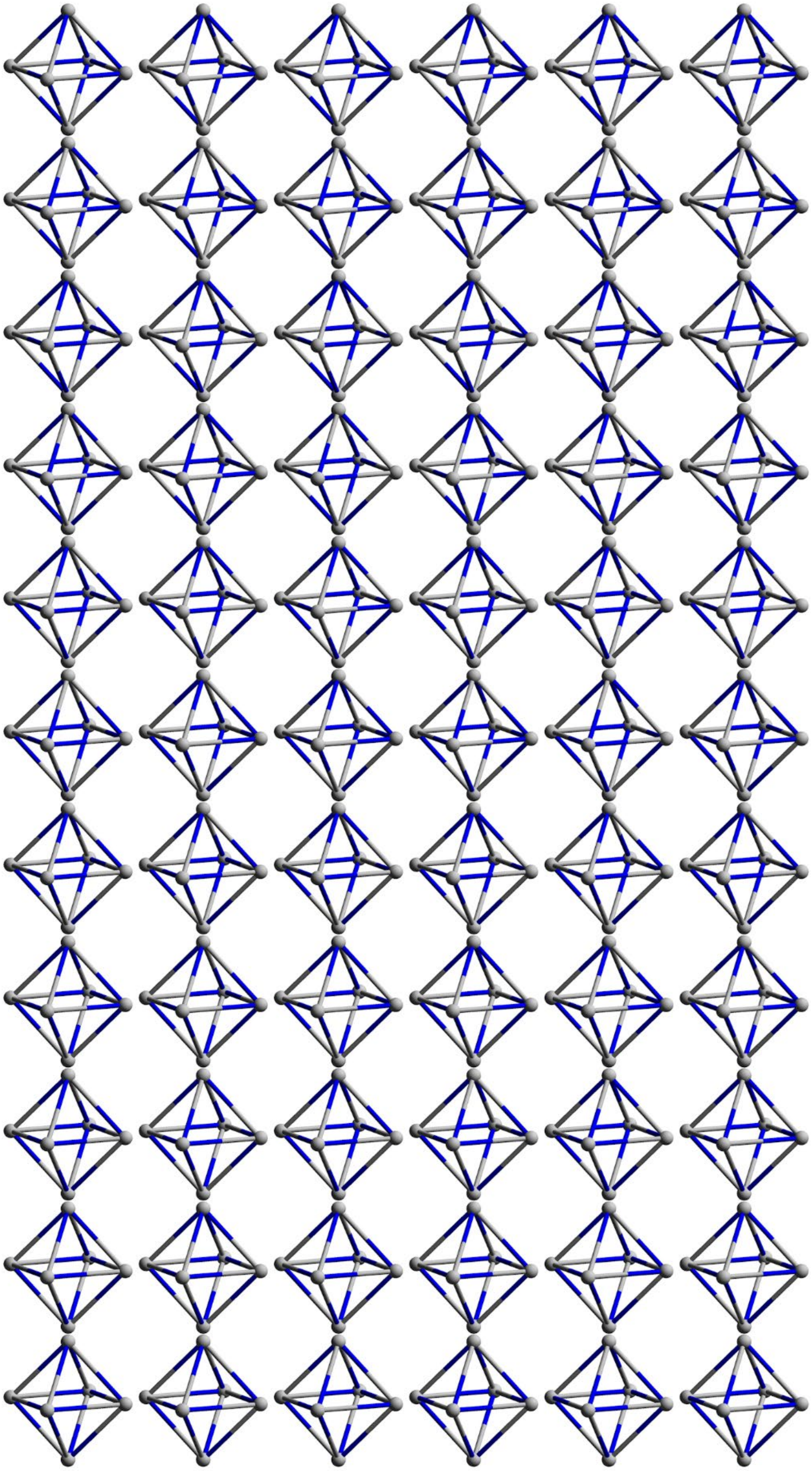

Figure S3a – page 1/3: 3D projection of the 186 isomeric structures of the $\{M_6L_{12}\}$ octahedral cage. The ambidentate bridging ligands L (blue and gray on octahedral edge) link metal ions M (octahedral corner).

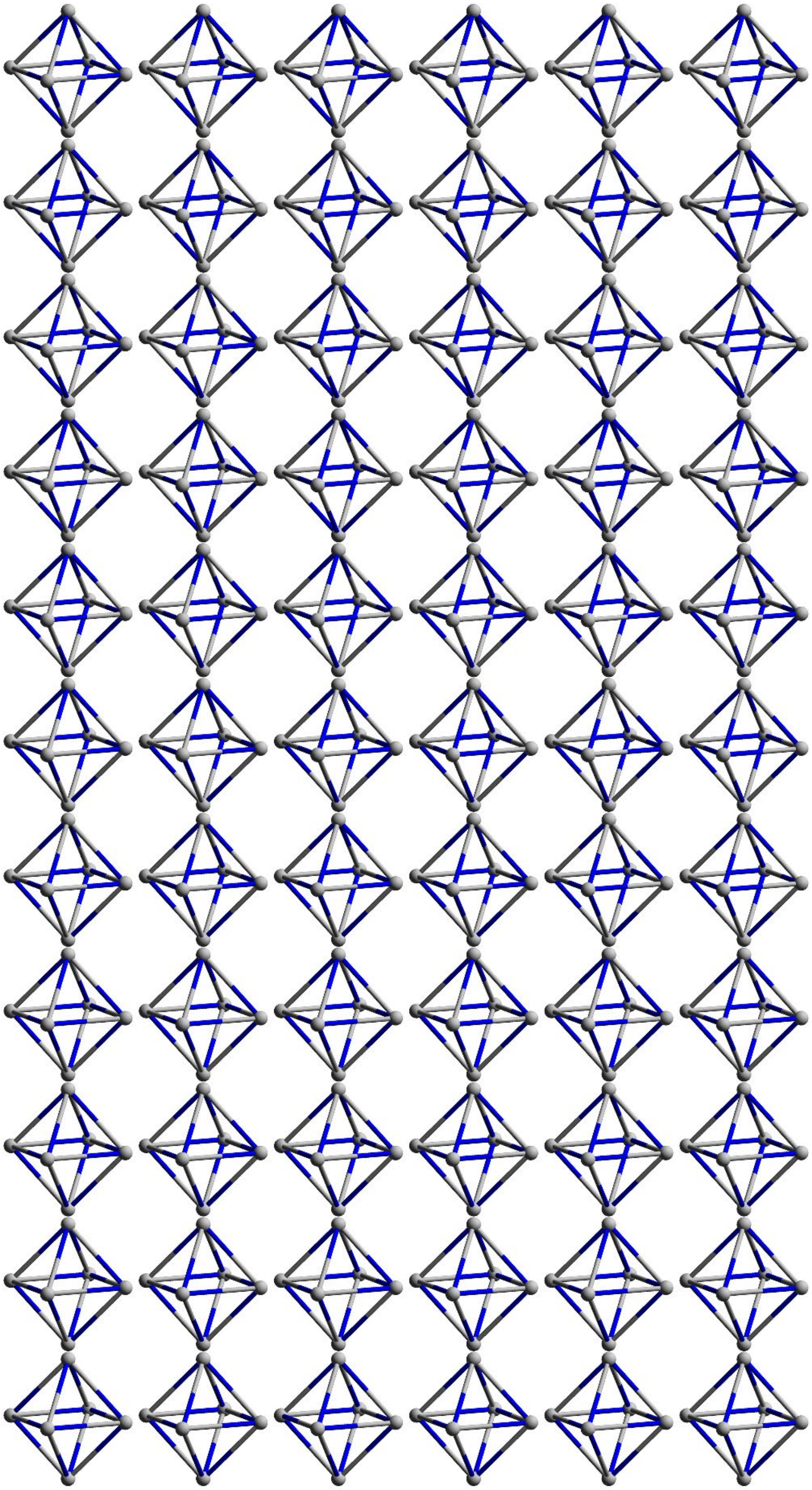

Figure S3a – page 2/3: 3D projection of the 186 isomeric structures of the $\{M_6L_{12}\}$ octahedral cage. The ambidentate bridging ligands L (blue and gray on octahedral edge) link metal ions M (octahedral corner).

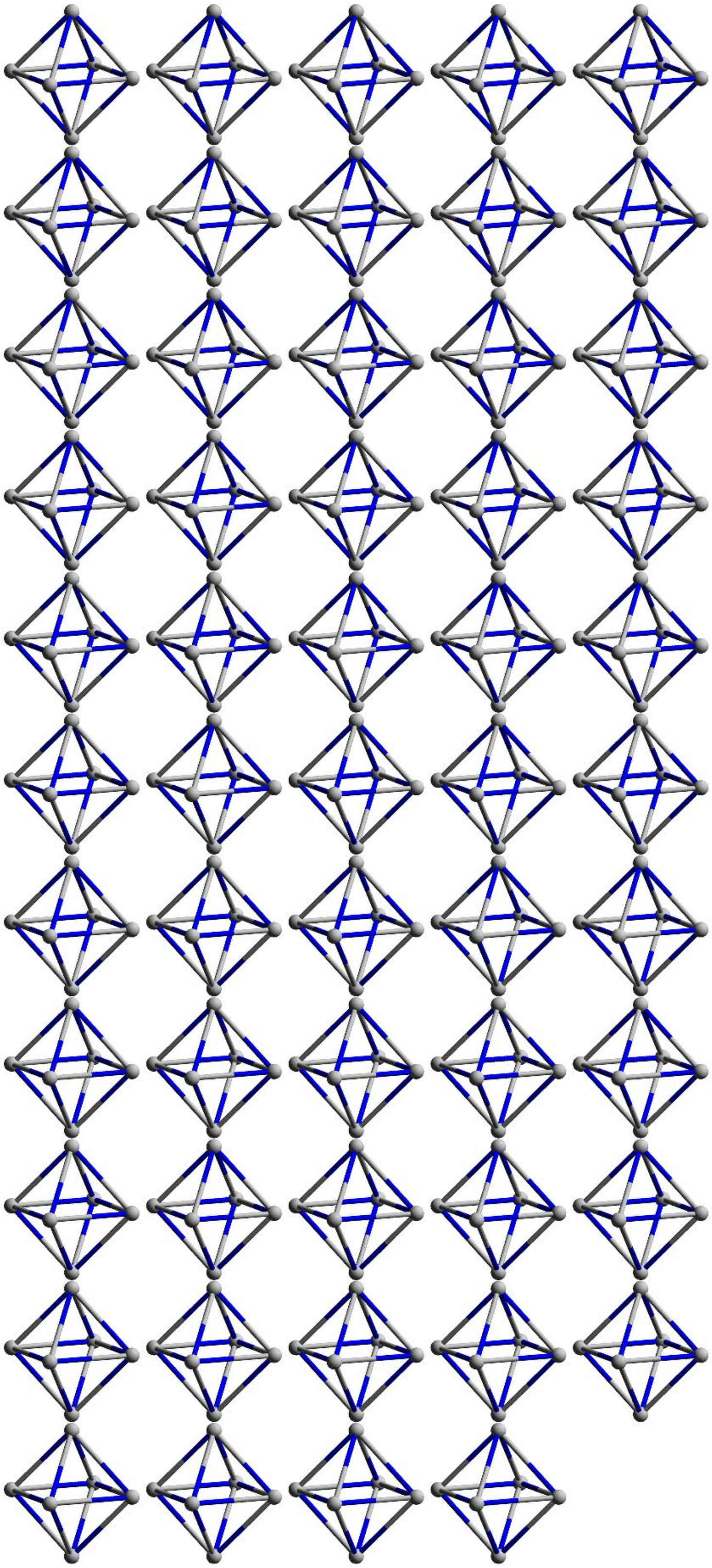

Figure S3a – page 3/3: 3D projection of the 186 isomeric structures of the $\{M_6L_{12}\}$ octahedral cage. The ambidentate bridging ligands L (blue and gray on octahedral edge) link metal ions M (octahedral corner).

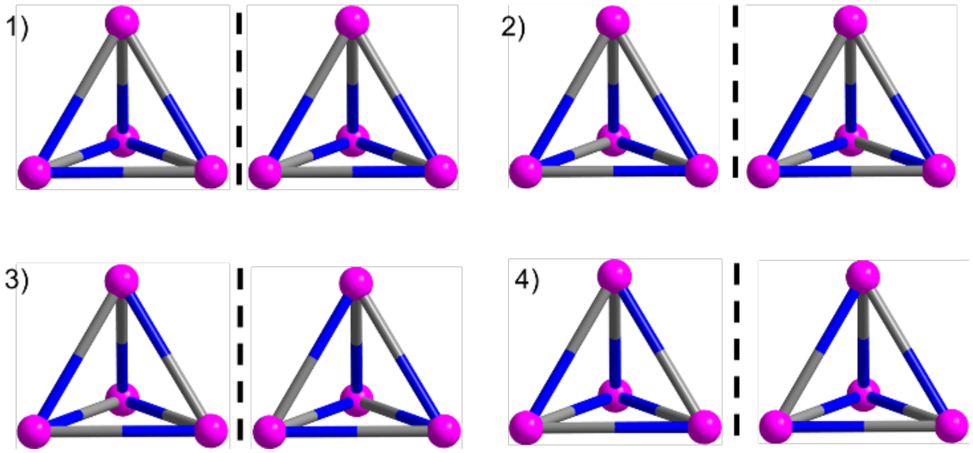

Figure S3b: 3D projection of $\{M_4L_6\}$ tetrahedral cage isomeric structures. The ambidentate bridging ligands L (blue and gray) link metal ions M (pink).

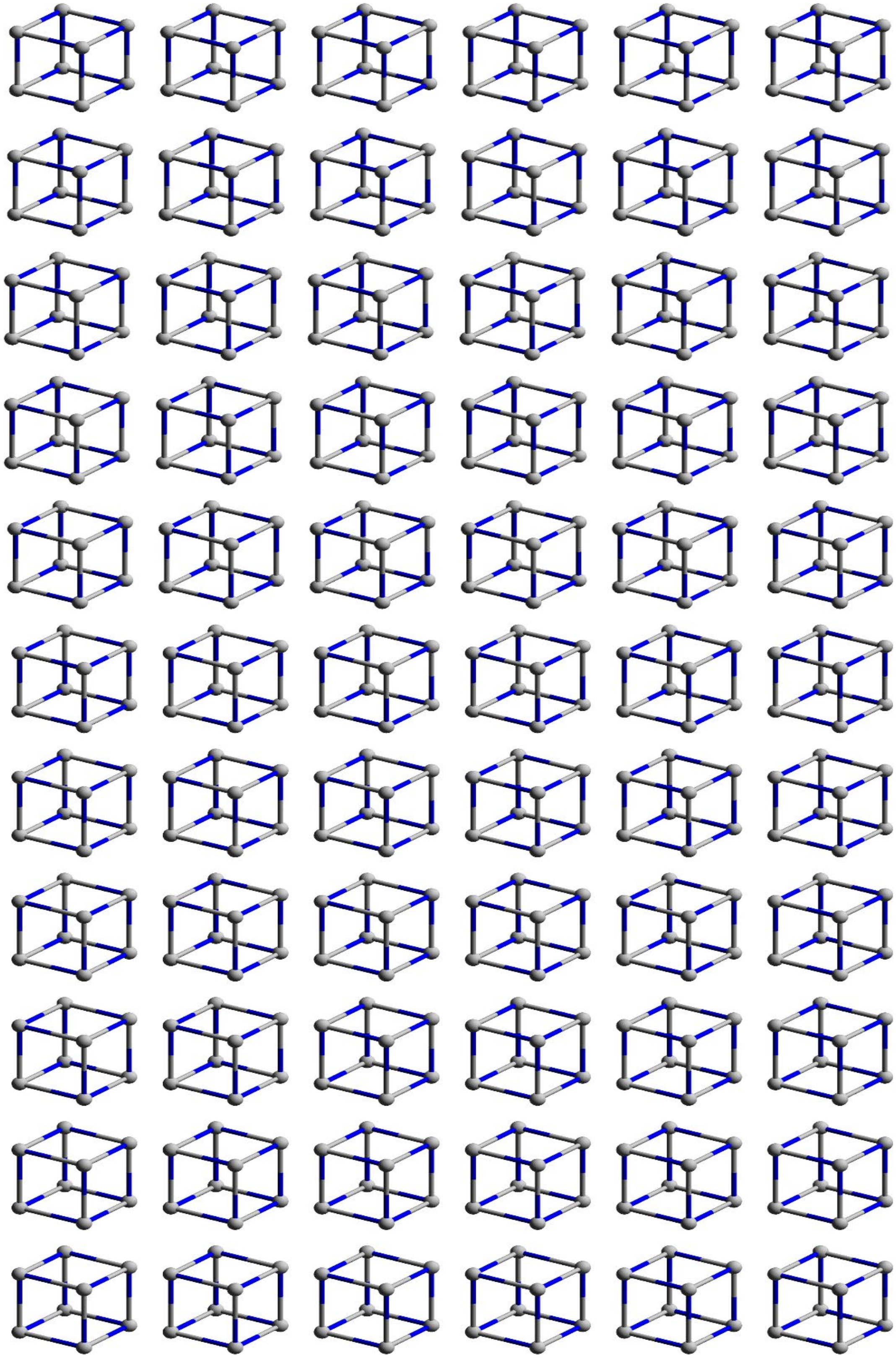

Figure S3c – page 1/3: 3D projection of the 186 isomeric structures of the $\{M_8L_{12}\}$ cubic cage. The ambidentate bridging ligands L (blue and gray on cube edge) link metal ions M (cube corner).

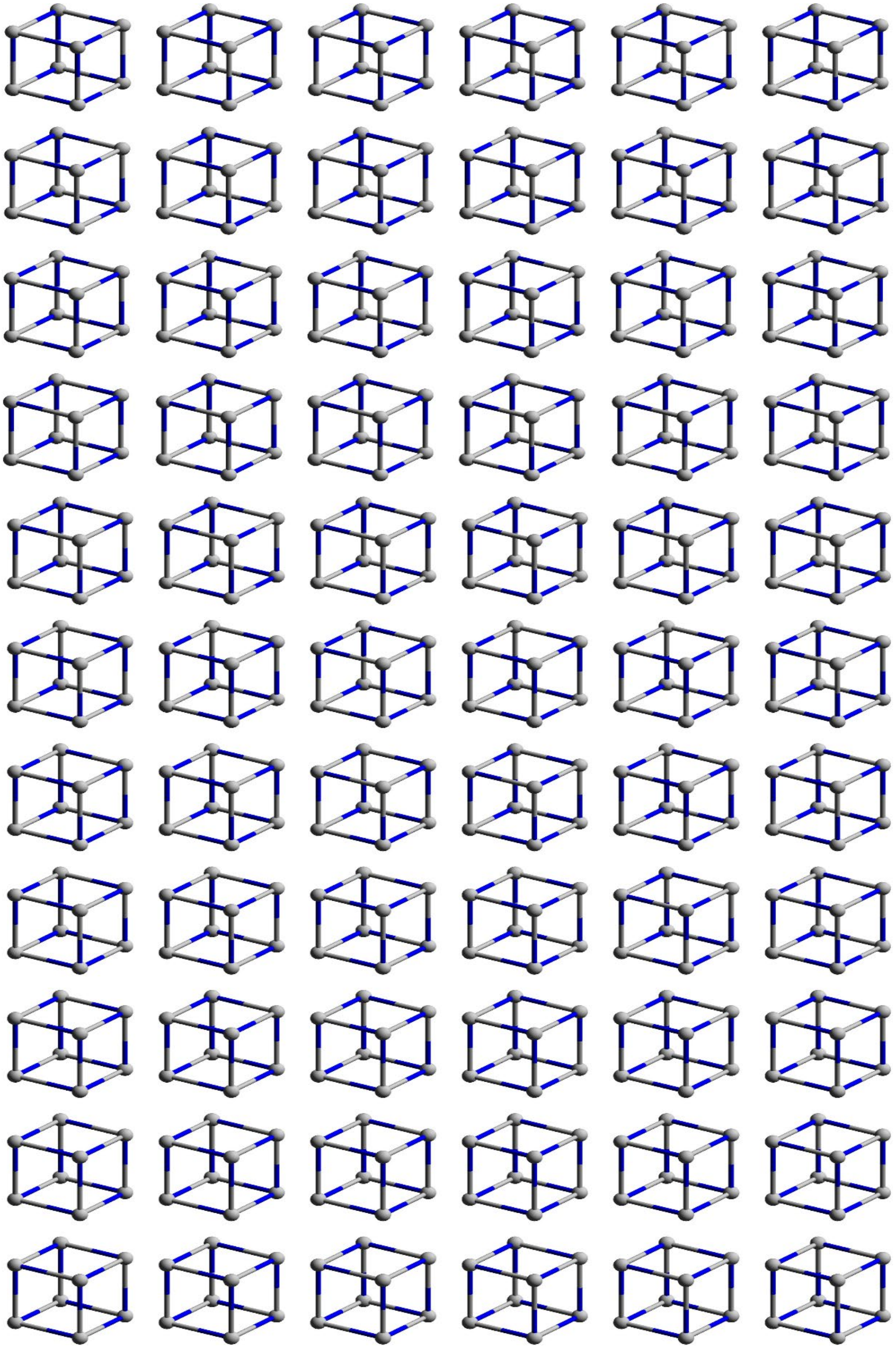

Figure S3c – page 2/3: 3D projection of the 186 isomeric structures of the $\{M_8L_{12}\}$ cubic cage. The ambidentate bridging ligands L (blue and gray on cube edge) link metal ions M (cube corner).

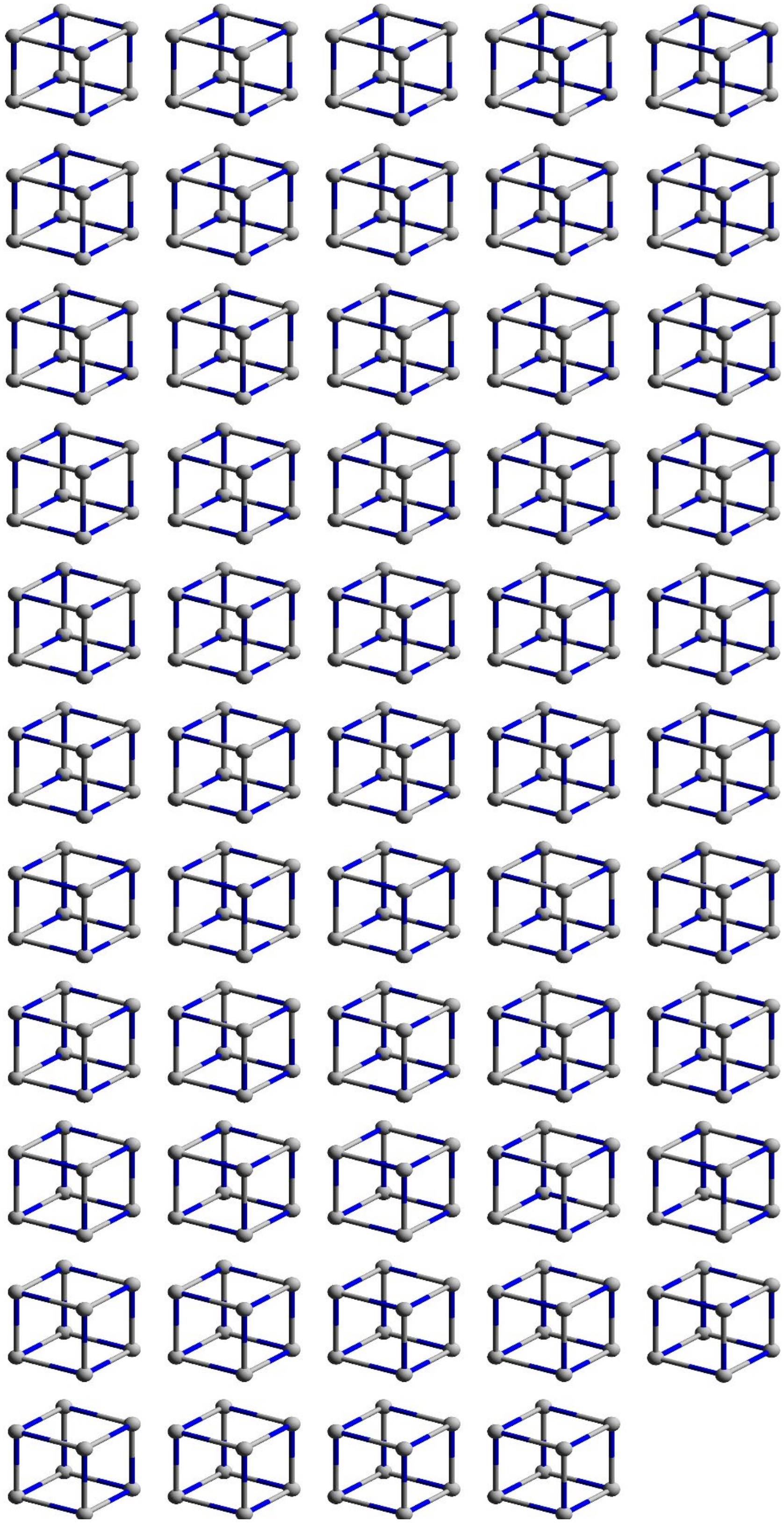

Figure S3c – page 2/3: 3D projection of the 186 isomeric structures of the $\{M_8L_{12}\}$ cubic cage. The ambidentate bridging ligands L (blue and gray on cube edge) link metal ions M (cube corner).

4. Crystallographic Data

All compounds obtained have been characterized by single crystal X-ray diffraction for structure determination, displaying octahedral-shaped $\{Bi_6Fe_{13}L_{12}\}$-type cluster. The octahedral skeleton shows that a tetrahedral FeO_4 central unit is encapsulated in discrete $\{Fe_{12}\}$ ball, resulting an unstable polynuclear α-Keggin $[(FeO_4)Fe_{12}O_{12}(OH)_{12}]^{5-}$ oxo/hydroxyl core, whose six square faces are further capped by six Bi^{3+} ions $\{Bi_6Fe_{13}\}$. The $[(FeO_4)Fe_{12}O_{12}(OH)_{12}]^{5-}$ α-Keggin ball can also be considered as constructed by four $\{Fe_3\}$ triads. Each triad is formed by three edge-shared FeO_6 octahedra and four of such edge-shared triads are linked together by corner sharing. Within the triads, the bridging ligands of the edge-sharing octahedra are hydroxyls, and the ligands between the corner-sharing octahedra are oxo ligands. The polynuclear iron core was further stabilized by coordination from twelve carboxylate ligands resulting in a univalent cationic oxo cluster, and thus it crystallizes with univalent anions. Each Bi vertex can be bridged by zero to four carboxylate ligands.

Close review of the crystal structures determined for compounds **1-6**, **1'** and **6a** reveals that there is no disorder on metal positions in the main polynuclear α-Keggin iron oxo core and bismuth ions. The Bi^{3+} cation coordination number is generally 8 and all Bi^{3+} centers in all compounds possess at least two 'exo' water molecule ligands ($Bi-OH_2$) with specific bond lengths of 2.8 Å. Each acetate ligand bridges a pair of Fe^{3+} ion and Bi^{3+} ion. Bond Valance Sum (BVS) calculations confirmed that both bismuth and iron atoms are all in +3 oxidation states, respectively, (see **Table S4**). Our research has shown that the highly symmetric clusters tend to crystallize in high symmetric crystal systems, such as cubic and tetragonal, in which ligand positions are unresolvable in crystal structure determinations. Therefore, we sought to grow crystals in lower symmetry system to fully resolve the ligand positions. Though our attempts with slowing crystallization using very low concentration was successful, the approach of adding small electrolytes, e.g., PF_6^-, I^-, ClO_{4-} and BF_4^-, did not yield the desired crystals.

Our research has shown that low pKa acetate ligands highly favors the formation of $Bi_6Fe_{13}L_{12}$ type octahedral cluster complexes, which also tend to crystallize in high symmetric crystal systems. For instance, the tetragonal system with a ≈ 26 Å and c ≈ 16 Å for compound **3** and **4**, in which ligand positions are found to be disordered over the two half Bi-Fe edges and therefore isomer types are unresolvable in crystal structure determinations. Our attempted efforts of slowing crystallization by using very low concentration in order to minimize the crystallographic symmetry, such as orthorhombic, monoclinic or triclinic were successful. For example, compound **5** was originally obtained in bulk scale with crystals having tetragonal system a ≈ 25.6 Å and c ≈ 14.6 Å and space group *I*-42d, similar to compound **3** and **4**. By very low concentration crystallization, we obtained high quality crystals with significantly low "G6 proj dist" value

for the orthorhombic lattice, see **Figure S4**, although highest possible system still automatically points to tetragonal. The structure was solved in orthorhombic system $P2_12_12_1$ space group selected manually and the structure refinement revealed that the asymmetric unit doesn't contain any positional disorders for the monofluoroacetate ligands. Therefore, configurational isomer of compound **5** has been undoubtedly identified.

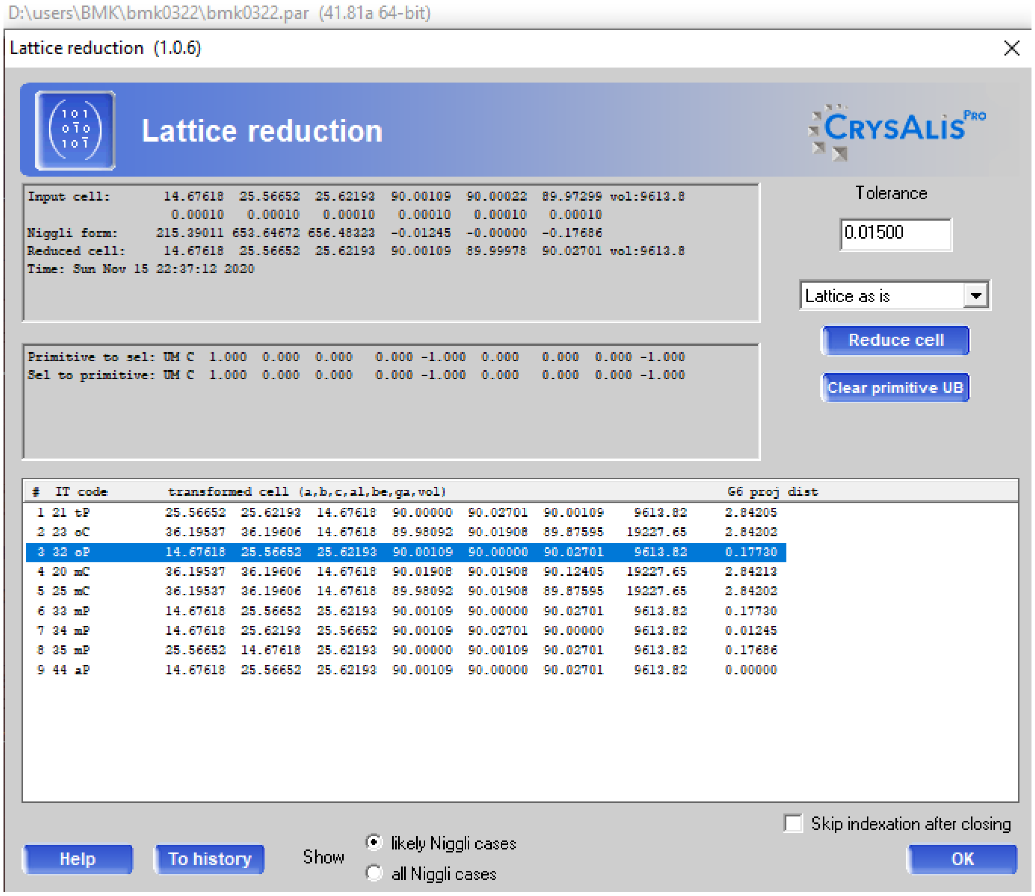

Figure S4. Brigg lattice determination in CrysAlis^{Pro} for compound **5**

Table S2. Brief summary of crystal data for comparison of lattices, unit cells and volumes per cluster (VPC)†

Compound	Carboxylates	Crystal Lattice	Space Group	Z	Unit cell parameters							
					a (Å)	b (Å)	c (Å)	α (°)	β (°)	γ (°)	Volume ($Å^3$)	Volume per cluster ($Å^3$)
**Keggin-3**	$CCl_3CO_2^-$	Monoclinic	*C*2/*c*	4	29.40	29.85	20.09	90	108.4	90	16720	4230
**Keggin-2**	$CF_3CO_2^-$	Cubic	*Pa*-3	8	27.66	27.66	27.66	90	90	90	21162	2645
**1**	$CF_3CO_2^-$	Monoclinic	*C*2/c	4	16.06	41.23	19.20	90	107.8	90	12113	3028
**1’**	$CF_3CO_2^-$	Triclinic	*P*-1	2	16.15	19.32	22.24	83.6	68.7	72.3	6169	3085
**2**	$CHCl_2CO_2^-$	Trigonal	*R*-3	3	21.48	21.48	26.82	90	90	120	10720	3573
**3**	$CHF_2CO_2^-$	Tetragonal	I-4_2d	4	25.59	25.59	16.01	90	90	90	10490	2622
**4**	$CH_2ClCO_2^-$	Tetragonal	I-4_2d	4	26.17	26.17	16.47	90	90	90	11292	2823
**5**	$CH_2FCO_2^-$	Orthorhombic	$P2_12_12_1$	4	14.67	25.55	25.60	90	90	90	9600	2400
**6**	$CClF_2CO_2^-$	Cubic	*Im*-3	8	31.62	31.62	31.62	90	90	90	31601	3950
**6a**	$CClF_2CO_2^-$	Cubic	*Im*-3	8	31.65	31.65	31.65	90	90	90	31712	3964

† Volume per cluster (VPC) is defined by the volume that each cluster with associated cation and anion and solvent molecules averagely occupies in solid state single crystal structue. It is calculated from the unit cell volume divided by number of the clusters (Z) in the unit cell.

Table S3. Crystal data and structure refinement details.

Compound code	**1**	**1'**	**2**
Empirical formula	$C_{32}H_{84}Bi_6F_{48}Fe_{13}Na_3O_{96}$	$C_{32}H_{92}Bi_6F_{48}Fe_{13}Na_3O_{100}$	$C_{30}H_{89}Bi_6Cl_{30}Fe_{13}Na_2O_{89}$
Formula weight	4965.89	5037.95	4963.42
Temperature (K)	100(2)	150(2)	150(2)
Crystal system	Monoclinic	Triclinic	Trigonal
Space group	*C*2/*c*	*P*-1	*R*-3
a (Å)	16.0675(2)	16.156(8)	21.481(3)
b (Å)	41.2393(6)	19.329(9)	21.481(3)
c (Å)	19.2007(4)	22.242(11)	26.826(5)
α (°)	90	83.676(8)	90
β (°)	107.802(2)	68.742(12)	90
γ (°)	90	72.379(9)	120
Volume (Å^3)	12113.4(4)	6169(5)	10720(3)
Z	4	2	3
Density calc'ed (Mg/m^3)	2.723	2.712	2.307
Absorption coefficient (mm^{-1})	10.386	10.201	9.292
F(000)	9380	4770	7047
θ range for data collection	2.268 to 26.000°	2.211 to 26.000°	1.332 to 25.987°
Index ranges	$-19 <= h <= 19$, $-45 <= k <= 50$, $-23 <= l <= 23$	$-19 <= h <= 19$, $-23 <= k <= 23$, $-26 <= l <= 27$	$-26 <= h <= 26$, $-26 <= k <= 18$, $-33 <= l <= 33$
Reflections collected	65159	103942	40530
Independent reflections	11893 [*R*(int) = 0.0378]	24241 [*R*(int) = 0.0475]	4677 [*R*(int) = 0.0609]
Completeness (θ = 25.242)	99.8%	99.9%	99.8%
Data/restraints/parameters	11893/258/987	24241/300/1775	4677/324/433
Goodness-of-fit on F^2	1.038	1.019	1.181
Final *R* indices [$I > 2\sigma(I)$]	*R*1 = 0.0276, w*R*2 = 0.0592	*R*1 = 0.0315, w*R*2 = 0.0760	*R*1 = 0.0472, w*R*2 = 0.1043
R indices (all data)	*R*1 = 0.0354, w*R*2 = 0.0615	*R*1 = 0.0410, w*R*2 = 0.0816	*R*1 = 0.0509, w*R*2 = 0.1063
Max/min $\Delta\rho$ (e Å^{-3})	1.315 and -1.013	2.32 and -1.09	1.95 and -1.17

Table S3. Crystal data and structure refinement details (continued).

Compound code	**3**	**4**	**5**
Empirical formula	$C_{28}H_{50}Bi_6F_{28}Fe_{13}NaO_{68}$	$C_{30}H_{72}Bi_6Cl_{15}Fe_{13}Na_2O_{73}$	$C_{24}H_{82}Bi_6F_{12}Fe_{13}N_2NaO_{81}$
Formula weight	4009.60	4158.53	3925.83
Temperature (K)	150(2)	150(2)	150(2)
Crystal system	Tetragonal	Tetragonal	Orthorhombic
Space group	I-42d	I-42d	$P2_12_12_1$
a (Å)	25.599(3)	26.1767(2)	14.6733(1)
b (Å)	25.599(3)	26.1767(2)	25.5522(3)
c (Å)	16.008(4)	16.4794(4)	25.6066(2)
Volume (Å^3)	10490(4)	11292.0(3)	9600.81(15)
Z	4	4	4
Density calc'ed (Mg/m^3)	2.539	2.446	2.716
Absorption coefficient (mm^{-1})	11.908	11.383	12.995
F(000)	7444	7796	7372
θ range for data collection	1.500 to 25.989°	2.134 to 25.998°	2.257 to 25.996°
Index ranges	$-31 <= h <= 31$, $-31 <= k <= 31$, $-19 <= l <= 19$	$-32 <= h <= 32$, $-32 <= k <= 31$, $-20 <= l <= 20$	$-18 <= h <= 18$, $-31 <= k <= 30$, $-31 <= l <= 31$
Reflections collected	72583	53453	88310
Independent reflections	5161 [R(int) = 0.0607]	5539 [R(int) = 0.0264]	18710 [R(int) = 0.0348]
Completeness (θ = 25.242)	100.0%	99.8%	99.4%
Data/restraints/parameters	516 /46/281	5539/76/329	18710/91/1235
Goodness-of-fit on F^2	1.137	1.060	1.029
Final R indices [$I > 2\sigma(I)$]	$R1$ = 0.0499, w$R2$ = 0.1307	$R1$ = 0.0334, w$R2$ = 0.0958	$R1$ = 0.0258, w$R2$ = 0.0546
R indices (all data)	$R1$ = 0.0730, w$R2$ = 0.1623	$R1$ = 0.0388, w$R2$ = 0.1018	$R1$ = 0.0312, w$R2$ = 0.0563
Flack parameter	0.054(6)	-0.022(4)	-0.0231(19)
Max/min Δρ (e Å^{-3})	2.58 and -1.60	0.87 and -1.39	1.160 and -1.325

Table S3. Crystal data and structure refinement details (continued).

Compound code	**6**	**6a**
Empirical formula	$C_{40}H_{51}Bi_6Cl_{17}F_{34}Fe_{13}N_3Na_4O_{77}$	$C_{40}H_{41}Bi_6Cl_{17}Cs_4F_{34}Fe_{13}N_3O_{72}$
Formula weight	5126.37	5475.98
Temperature (K)	150(2)	150(2)
Crystal system	Cubic	Cubic
Space group	*Im*-3	*Im*-3
a (Å)	31.6156(2)	31.6528(2)
b (Å)	31.6156(2)	31.6528(2)
c (Å)	31.6156(2)	31.6528(2)
Volume (Å^3)	31601.3(6)	31712.9(6)
Z	8	8
Density calc'ed (Mg/m^3)	2.155	2.294
Absorption coefficient (mm^{-1})	8.227	9.088
F(000)	19224	20232
θ range for data collection	2.410 to 25.972°	2.408 to 25.994°
Index ranges	$-38 <= h <= 38$,<br>$-38 <= k <= 38$,<br>$-38 <= l <= 38$	$-39 <= h <= 39$,<br>$-39 <= k <= 39$,<br>$-38 <= l <= 39$
Reflections collected	164660	151562
Independent reflections	5472 [*R*(int) = 0.0888]	5491 [*R*(int) = 0.0589]
Completeness (θ = 25.242)	99.9%	99.8 %
Data/restraints/parameters	5472 / 115 / 306	5491 / 117 / 319
Goodness-of-fit on F^2	1.059	1.031
Final *R* indices [$I > 2\sigma(I)$]	*R*1 = 0.0447, w*R*2 = 0.1114	*R*1 = 0.0490, w*R*2 = 0.1424
R indices (all data)	*R*1 = 0.0542, w*R*2 = 0.1165	*R*1 = 0.0559, w*R*2 = 0.1490
Max/min Δρ (e Å^{-3})	1.58 and -1.24	1.93 and -1.06

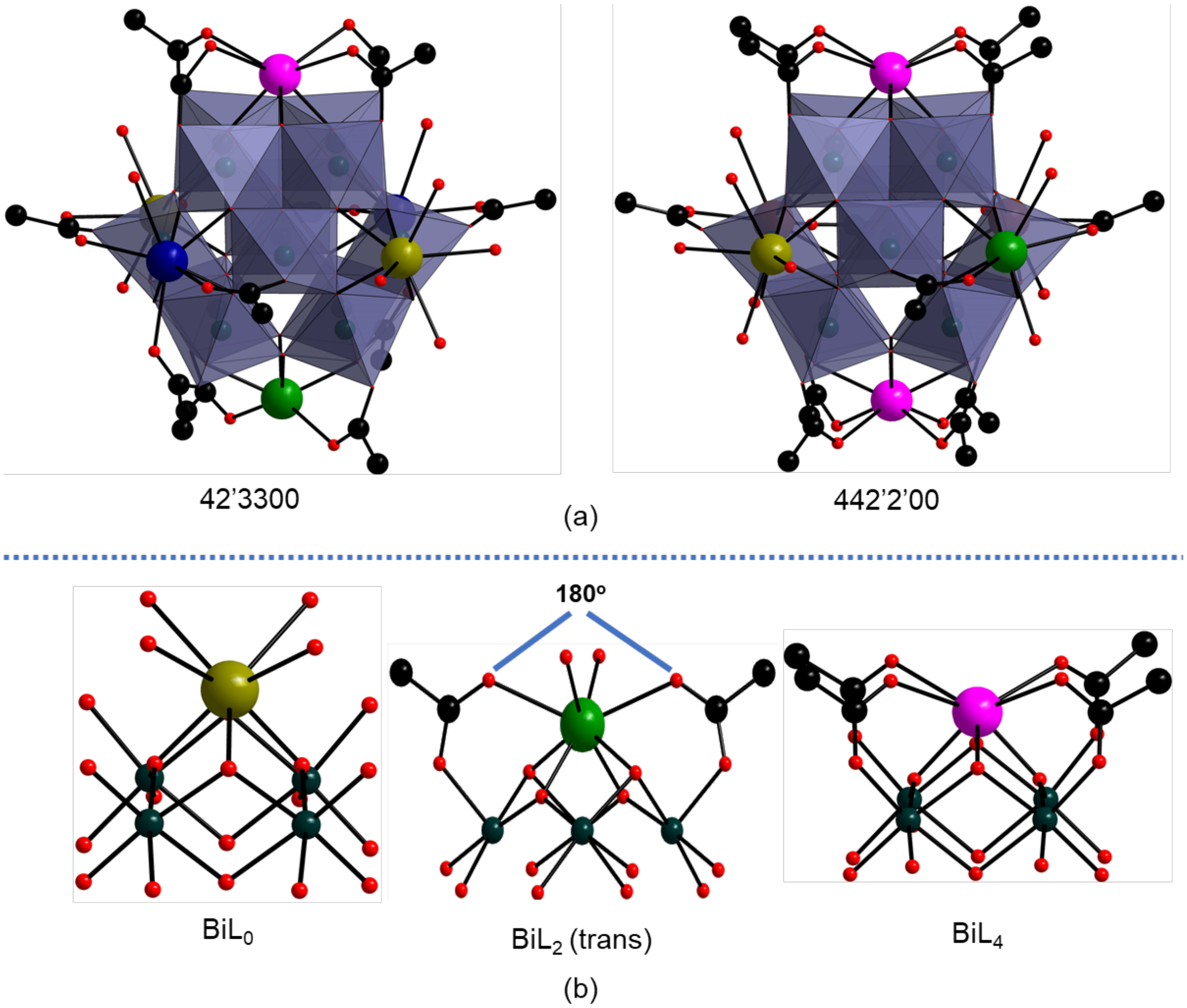

Figure S5a. (a) Solid state structure of **Keggin-3** (42’3300 or 442’2’00 isomers pending on the two disorder positions of the disordered ligand); (b) different Bi coordination modes in **Keggin-3**. For the “2’” Bi vertice, two acetate ligands are on trans positions (180º) and their ligand planes are coplanar.

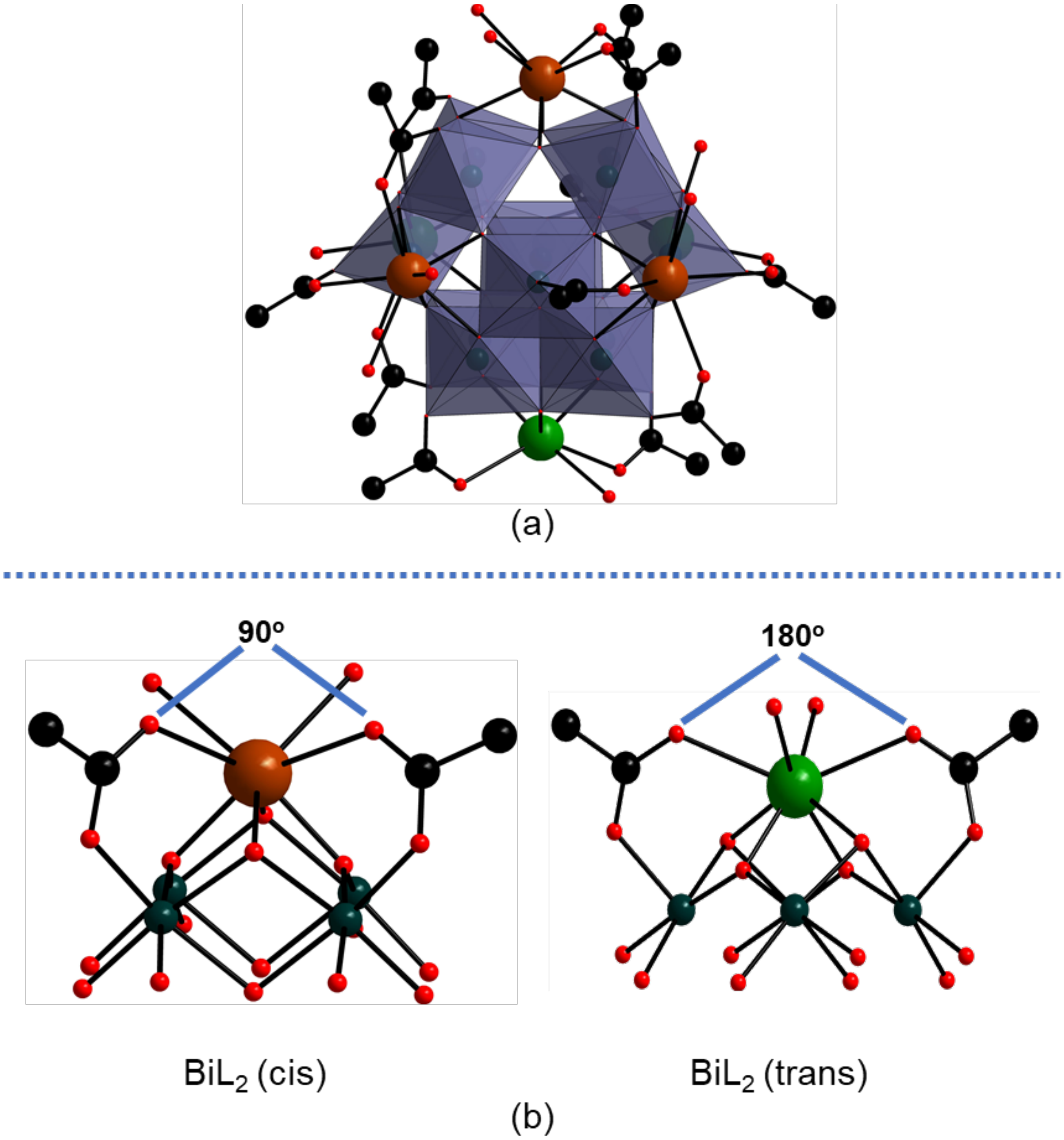

Figure S5b. (a) Solid state structure of **Keggin-2** (22’22’22’) and (b) different coordination modes of Bi in **Keggin-2**. For the “2” Bi vertice, two acetate ligands are on cis positions (90°) and their ligand planes are vertical to each others.

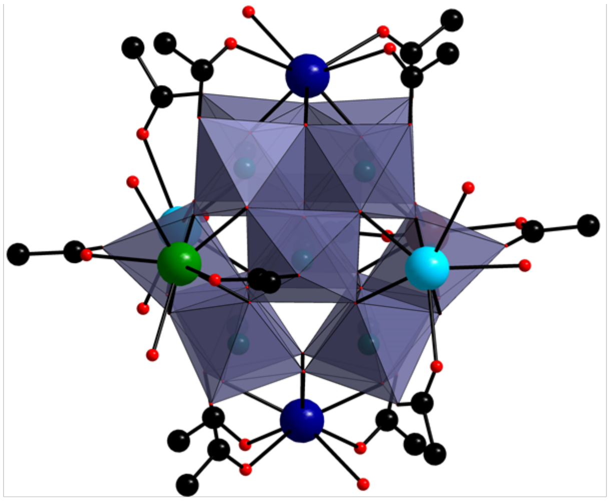

(a)

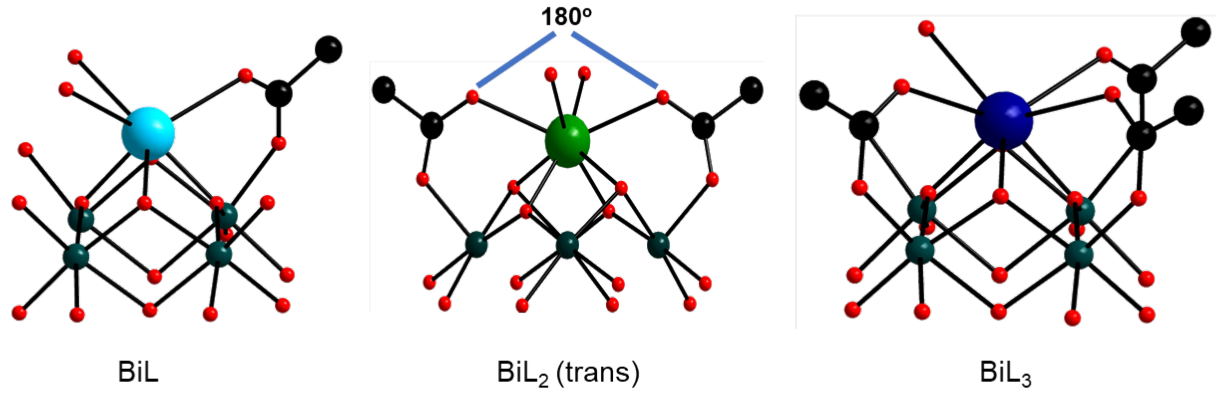

(b)

Figure S5c. (a) Solid state structure of compound **1** (332’2’11 isomer) and (b) different Bi coordination modes of compound **1** (332’2’11 isomer)

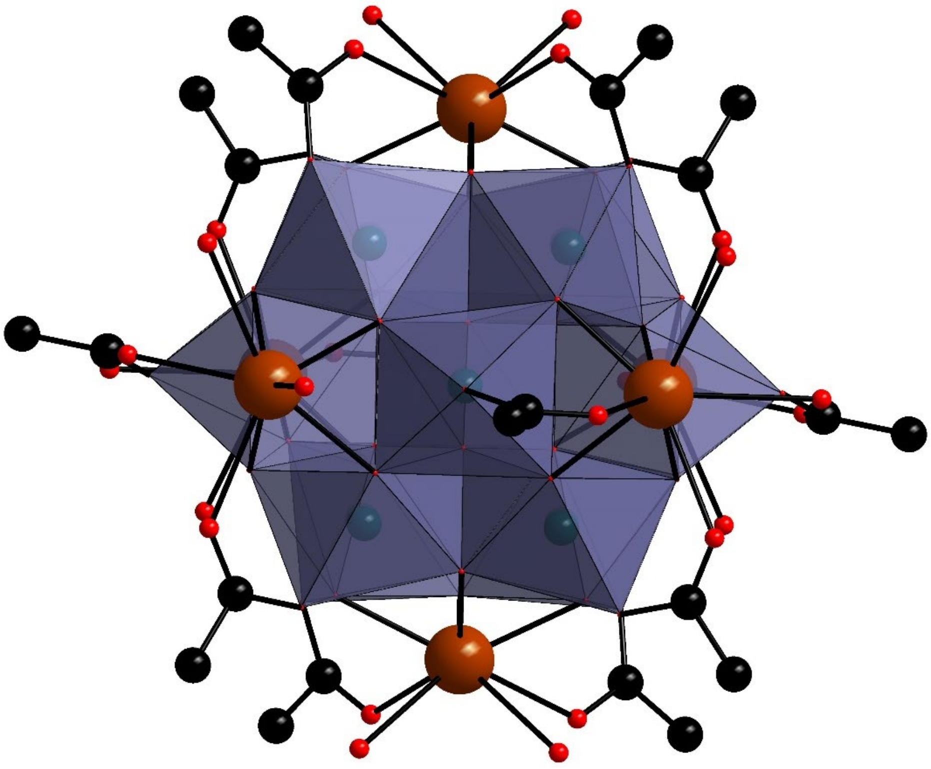

Figure S5d. Solid-state structure of compounds **2, 5** and **6** (222222 isomer)

5. Bond Valance Sum

BVS calculations were carried out for all compounds. Parameters were taken from Gagne & Hawthorne.[10] Mean bond valence sum values are listed for each compound in **Table S4**. Following the table are the detailed calculations.

Table S4. Mean bond valence sum (BVS) values for Bi^{3+} and Fe^{3+} in each compounds

Compound	**1**	**1’**	**2**	**3**	**4**	**5**	**6**	**6a**
Bi^{3+}	3.031	2.966	2.965	3.024	3.094	3.081	3.118	3.124
Fe^{3+}	2.984	2.926	3.085	3.002	3.059	3.036	2.997	2.922

Detailed bond valence sum calculations

**Parameter list**															
		R0 (A)	B (A)												
Fe(3+)-O		1.766	0.360					BVS=EXP((R0-R)/B)							
Bi(3+)-O		2.068	0.389												
**Compound 1**															
Bond		R	BVS	Bond		R	BVS	Bond		R	BVS	Bond		R	BVS
Bi1	O3	2.387	0.440	Bi2	O7	2.385	0.443	Bi3	O9	2.200	0.712	Bi4	O13	2.161	0.787
Bi1	O4	2.172	0.765	Bi2	O8	2.190	0.731	Bi3	O9	2.200	0.712	Bi4	O13	2.161	0.787
Bi1	O5	2.373	0.457	Bi2	O11	2.417	0.408	Bi3	O10	2.412	0.413	Bi4	O14	2.365	0.466
Bi1	O6	2.180	0.750	Bi2	O12	2.184	0.742	Bi3	O10	2.412	0.413	Bi4	O14	2.365	0.466
Bi1	O18	2.759	0.169	Bi2	O15	2.700	0.197	Bi3	O17	2.730	0.182	Bi4	O32	2.786	0.158
Bi1	O26	2.748	0.174	Bi2	O16	2.754	0.171	Bi3	O17	2.730	0.182	Bi4	O32	2.786	0.158
Bi1	O22	2.774	0.163	Bi2	O28	2.734	0.180	Bi3	O30	2.687	0.204	Bi4	O33	2.901	0.117
Bi1	O24	2.861	0.130	Bi2	O45	2.855	0.132	Bi3	O30	2.687	0.204	Bi4	O33	2.901	0.117
Sum			3.049				3.004				3.023				3.058
				Fe2	O1	2.072	0.427	Fe3	O2	2.083	0.415	Fe4	O2	2.096	0.400
				Fe2	O3	2.099	0.397	Fe3	O8	1.934	0.627	Fe4	O4	1.920	0.652
Fe1	O1	1.875	0.739	Fe2	O4	1.918	0.656	Fe3	O9	1.906	0.678	Fe4	O5	2.097	0.399
Fe1	O1	1.875	0.739	Fe2	O7	2.090	0.407	Fe3	O10	2.107	0.388	Fe4	O9	1.925	0.643
Fe1	O2	1.874	0.741	Fe2	O8	1.914	0.663	Fe3	O11	2.086	0.411	Fe4	O10	2.045	0.461
Fe1	O2	1.874	0.741	Fe2	O27	2.064	0.437	Fe3	O29	2.077	0.422	Fe4	O21	2.059	0.443
Sum			2.959				2.986				2.940				2.997
Fe5	O2	2.076	0.423	Fe6	O1	2.079	0.419	Fe7	O1	2.103	0.392				
Fe5	O5	2.092	0.404	Fe6	O7	2.080	0.418	Fe7	O3	2.089	0.408				
Fe5	O6	1.906	0.678	Fe6	O12	1.936	0.624	Fe7	O6	1.902	0.685				
Fe5	O11	2.082	0.416	Fe6	O13	1.914	0.663	Fe7	O13	1.927	0.639				
Fe5	O12	1.932	0.631	Fe6	O14	2.096	0.400	Fe7	O14	2.064	0.437				
Fe5	O23	2.061	0.441	Fe6	O31	2.065	0.436	Fe7	O25	2.031	0.479				
Sum			2.992				2.959				3.041				

**Compound 1'**															
Bi1	O1	2.381	0.447	Bi2	O5	2.424	0.400	Bi3	O9	2.429	0.395	BI4	O13	2.379	0.450
Bi1	O2	2.180	0.750	Bi2	O6	2.219	0.678	Bi3	O10	2.195	0.721	Bi4	O14	2.165	0.779
Bi1	O3	2.394	0.433	Bi2	O7	2.428	0.396	Bi3	O11	2.390	0.437	BI4	O15	2.378	0.451
Bi1	O4	2.178	0.754	Bi2	O8	2.221	0.675	Bi3	O12	2.202	0.709	Bi4	O16	2.169	0.771
Bi1	O32	2.872	0.127	Bi2	O38	2.689	0.203	Bi3	O42	2.710	0.192	BI4	O44	2.806	0.150
Bi1	O34	2.752	0.172	Bi2	O39	2.688	0.203	Bi3	O66	2.719	0.188	Bi4	O46	2.805	0.150
Bi1	O36	2.769	0.165	Bi2	O64	2.718	0.188	Bi3	O72	2.846	0.135	BI4	O56	2.932	0.108
Bi1	O63	2.778	0.161	Bi2	O65	2.718	0.188	Bi3	O74	2.771	0.164	Bi4	O59	2.930	0.109
Sum			3.008				2.932				2.941				2.969
Bi5	O17	2.196	0.720	Bi6	O21	2.175	0.760								
Bi5	O18	2.392	0.435	Bi6	O22	2.383	0.445								
Bi5	O19	2.197	0.718	Bi6	O23	2.184	0.742					Fe1	O1	2.096	0.400
Bi5	O20	2.428	0.396	Bi6	O24	2.392	0.435					Fe1	O2	1.916	0.659
Bi5	O48	2.717	0.189	Bi6	O50	2.868	0.128					Fe1	O9	2.095	0.401
Bi5	O67	2.723	0.186	Bi6	O52	2.774	0.163					Fe1	O12	1.937	0.622
Bi5	O79	2.852	0.133	Bi6	O54	2.754	0.171					Fe1	O27	2.084	0.413
Bi5	O80	2.769	0.165	Bi6	O68	2.774	0.163					Fe1	O31	2.075	0.424
Sum			2.941				3.006								2.919
Fe2	O2	1.908	0.674	Fe3	O3	2.108	0.387	Fe4	O1	2.116	0.378	Fe5	O6	1.907	0.676
Fe2	O3	2.104	0.391	Fe3	O4	1.915	0.661	Fe4	O4	1.932	0.631	Fe5	O7	2.124	0.370
Fe2	O15	2.073	0.426	Fe3	O17	1.928	0.638	Fe4	O5	2.043	0.463	Fe5	O17	1.936	0.624
Fe2	O16	1.931	0.632	Fe3	O18	2.112	0.382	Fe4	O6	1.925	0.643	Fe5	O20	2.099	0.397
Fe2	O25	2.102	0.393	Fe3	O25	2.089	0.408	Fe4	O27	2.096	0.400	Fe5	O26	2.097	0.399
Fe2	O33	2.033	0.476	Fe3	O47	2.064	0.437	Fe4	O35	2.069	0.431	Fe5	O40	2.078	0.420
Sum			2.993				2.913				2.946				2.885
Fe6	O5	2.125	0.369	Fe7	O11	2.094	0.402	Fe8	O14	1.930	0.634	Fe9	O19	1.935	0.625
Fe6	O8	1.905	0.680	Fe7	O12	1.933	0.629	Fe8	O15	2.104	0.391	Fe9	O20	2.094	0.402
Fe6	O9	2.098	0.398	Fe7	O13	2.107	0.388	Fe8	O18	2.093	0.403	Fe9	O22	2.095	0.401
Fe6	O10	1.938	0.620	Fe7	O16	1.928	0.638	Fe8	O19	1.934	0.627	Fe9	O23	1.913	0.665
Fe6	O27	2.099	0.397	Fe7	O28	2.097	0.399	Fe8	O25	2.094	0.402	Fe9	O26	2.086	0.411
Fe6	O37	2.077	0.422	Fe7	O43	2.053	0.451	Fe8	O45	2.057	0.446	Fe9	O49	2.067	0.433
Sum			2.884				2.906				2.903				2.938
Fe10	O7	2.045	0.461	Fe11	O10	1.926	0.641	Fe12	O13	2.069	0.431				
Fe10	O8	1.923	0.647	Fe11	O11	2.107	0.388	Fe12	O14	1.931	0.632				
Fe10	O21	1.931	0.632	Fe11	O21	1.918	0.656	Fe12	O23	1.907	0.676	Fe13	O25	1.896	0.697
Fe10	O22	2.116	0.378	Fe11	O24	2.110	0.385	Fe12	O24	2.101	0.394	Fe13	O26	1.872	0.745
Fe10	O26	2.098	0.398	Fe11	O28	2.087	0.410	Fe12	O28	2.108	0.387	Fe13	O27	1.873	0.743
Fe10	O51	2.065	0.436	Fe11	O41	2.065	0.436	Fe12	O53	2.035	0.474	Fe13	O28	1.892	0.705
			2.951				2.915				2.994				2.889

**Compound 2**															
Bi1	O2	2.685	0.205												
Bi1	O3	2.740	0.178												
Bi1	O7	2.457	0.368					Fe2	O1	2.040	0.467	Fe3	O4	2.027	0.484
Bi1	O8	2.185	0.740					Fe2	O6	2.133	0.361	Fe3	O6	2.161	0.334
Bi1	O9	2.375	0.454	Fe1	O5	1.890	0.709	Fe2	O7	2.156	0.338	Fe3	O7	2.150	0.344
Bi1	O10	2.179	0.752	Fe1	O6	1.874	0.741	Fe2	O8	1.873	0.743	Fe3	O8	1.817	0.868
Bi1	O13	2.842	0.137	Fe1	O6	1.874	0.741	Fe2	O9	2.147	0.347	Fe3	O10	1.855	0.781
Bi1	O14	2.857	0.132	Fe1	O6	1.874	0.741	Fe2	O9'	1.852	0.788	Fe3	O10'	2.153	0.341
Sum			2.965				2.931				3.044				3.152
**Compound 3**															
Bi1	O1	2.773	0.163	Bi2	O10	2.371	0.459								
Bi1	O3	2.181	0.748	Bi2	O10	2.371	0.459								
Bi1	O4	2.377	0.452	Bi2	O12	2.173	0.763								
Bi1	O8	2.190	0.731	Bi2	O12	2.173	0.763								
Bi1	O9	2.406	0.419	Bi2	O14	2.833	0.140								
Bi1	O6	2.777	0.162	Bi2	O14'	2.756	0.171								
Bi1	O13	2.779	0.161	Bi2	O15'	2.812	0.148								
Bi1	O16	2.644	0.227	Bi2	O17	3.286	0.044								
Sum			3.063				2.947								
				Fe2	O3	1.911	0.668	Fe3	O2	2.061	0.441	Fe4	O5	2.076	0.423
				Fe2	O4	2.088	0.409	Fe3	O3	1.916	0.659	Fe4	O8	1.911	0.668
Fe1	O5	1.891	0.707	Fe2	O5	2.104	0.391	Fe3	O4	2.071	0.429	Fe4	O9	2.107	0.388
Fe1	O5	1.891	0.707	Fe2	O7	2.039	0.468	Fe3	O5	2.085	0.412	Fe4	O10	2.094	0.402
Fe1	O5	1.891	0.707	Fe2	O10	2.099	0.397	Fe3	O8	1.912	0.667	Fe4	O11	2.022	0.491
Fe1	O5	1.891	0.707	Fe2	O12	1.918	0.656	Fe3	O9	2.056	0.447	Fe4	O12	1.929	0.636
Sum			2.827				2.989				3.054				3.008
**Compound 4**															
Bi1	O2	2.659	0.219	Bi2	O9	2.191	0.729								
Bi1	O4	2.793	0.155	Bi2	O9	2.191	0.729								
Bi1	O5	2.153	0.804	Bi2	O10	2.388	0.439								
Bi1	O6	2.412	0.413	Bi2	O10	2.388	0.439								
Bi1	O7	2.654	0.222	Bi2	O18	2.763	0.168								
Bi1	O12	2.198	0.716	Bi2	O18	2.763	0.168								
Bi1	O13	2.351	0.483	Bi2	O22	2.782	0.160								
Bi1	O15	2.850	0.134	Bi2	O22	2.782	0.160								
Sum			3.145				2.991								
				Fe2	O5	1.936	0.624	Fe3	O1	2.036	0.472	Fe4	O3	2.048	0.457
				Fe2	O6	2.092	0.404	Fe3	O5	1.896	0.697	Fe4	O9	1.924	0.645
Fe1	O11	1.844	0.805	Fe2	O8	2.025	0.487	Fe3	O6	2.058	0.444	Fe4	O10	2.084	0.413
Fe1	O11	1.844	0.805	Fe2	O9	1.894	0.701	Fe3	O11	2.115	0.379	Fe4	O11	2.122	0.372
Fe1	O11	1.844	0.805	Fe2	O10	2.060	0.442	Fe3	O12	1.903	0.683	Fe4	O12	1.910	0.670
Fe1	O11	1.844	0.805	Fe2	O11	2.089	0.408	Fe3	O13	2.074	0.425	Fe4	O13	2.083	0.415
Sum			3.221				3.065				3.101				2.972

**Compound 5**															
Bi1	O5	2.333	0.506	Bi2	O9	2.387	0.440	Bi3	O13	2.203	0.707	Bi4	O17	2.179	0.752
Bi1	O6	2.172	0.765	Bi2	O10	2.147	0.816	Bi3	O14	2.402	0.424	Bi4	O18	2.361	0.471
Bi1	O7	2.429	0.395	Bi2	O11	2.402	0.424	Bi3	O15	2.143	0.825	Bi4	O19	2.210	0.694
Bi1	O8	2.161	0.787	Bi2	O12	2.182	0.746	Bi3	O16	2.384	0.444	Bi4	O20	2.425	0.399
Bi1	O31	2.750	0.173	Bi2	O35	2.591	0.261	Bi3	O39	2.561	0.282	Bi4	O43	2.753	0.172
Bi1	O33	2.764	0.167	Bi2	O37	2.731	0.182	Bi3	O42	2.699	0.197	Bi4	O45	2.639	0.230
Bi1	O61	2.884	0.123	Bi2	O63	2.988	0.094	Bi3	O65	2.815	0.147	Bi4	O67	2.676	0.210
Bi1	O62	2.854	0.133	Bi2	O64	2.833	0.140	Bi3	O66	3.040	0.082	Bi4	O68	2.773	0.163
Sum			3.050				3.103				3.107				3.091
Bi5	O21	2.200	0.712	Bi6	O25	2.195	0.721								
Bi5	O22	2.453	0.372	Bi6	O26	2.426	0.398								
Bi5	O23	2.198	0.716	Bi6	O27	2.149	0.812								
Bi5	O24	2.343	0.493	Bi6	O28	2.336	0.502								
Bi5	O48	2.712	0.191	Bi6	O52	2.748	0.174					Fe1	O1	1.862	0.766
Bi5	O49	2.623	0.240	Bi6	O54	2.808	0.149					Fe1	O2	1.866	0.757
Bi5	O69	2.737	0.179	Bi6	O71	2.741	0.177					Fe1	O3	1.867	0.755
Bi5	O70	2.755	0.171	Bi6	O72	2.865	0.129					Fe1	O4	1.877	0.735
Sum			3.074				3.063								3.013
Fe2	O1	2.103	0.392	Fe3	O2	2.074	0.425	Fe4	O1	2.087	0.410	Fe5	O2	2.105	0.390
Fe2	O5	2.082	0.416	Fe3	O6	1.926	0.641	Fe4	O14	2.094	0.402	Fe5	O7	2.099	0.397
Fe2	O6	1.920	0.652	Fe3	O7	2.078	0.420	Fe4	O15	1.932	0.631	Fe5	O8	1.913	0.665
Fe2	O13	1.917	0.657	Fe3	O9	2.079	0.419	Fe4	O21	1.904	0.682	Fe5	O17	1.915	0.661
Fe2	O14	2.061	0.441	Fe3	O10	1.945	0.608	Fe4	O22	2.054	0.449	Fe5	O18	2.071	0.429
Fe2	O32	2.038	0.470	Fe3	O36	2.059	0.443	Fe4	O41	2.035	0.474	Fe5	O46	2.039	0.468
Sum			3.028				2.957				3.047				3.009
Fe6	O1	2.097	0.399	Fe7	O3	2.112	0.382	Fe8	O2	2.126	0.368	Fe9	O3	2.087	0.410
Fe6	O5	2.087	0.410	Fe7	O10	1.912	0.667	Fe8	O9	2.061	0.441	Fe9	O15	1.928	0.638
Fe6	O8	1.946	0.607	Fe7	O11	2.093	0.403	Fe8	O12	1.936	0.624	Fe9	O16	2.098	0.398
Fe6	O22	2.090	0.407	Fe7	O13	1.919	0.654	Fe8	O18	2.079	0.419	Fe9	O25	1.932	0.631
Fe6	O23	1.908	0.674	Fe7	O16	2.046	0.459	Fe8	O19	1.911	0.668	Fe9	O26	2.064	0.437
Fe6	O34	2.016	0.499	Fe7	O38	2.036	0.472	Fe8	O44	2.045	0.461	Fe9	O40	2.055	0.448
Sum			2.995				3.038				2.981				2.961
Fe10	O3	2.098	0.398	Fe11	O4	2.086	0.411	Fe12	O4	2.120	0.374	Fe13	O4	2.067	0.433
Fe10	O11	2.061	0.441	Fe11	O17	1.917	0.657	Fe12	O21	1.906	0.678	Fe13	O19	1.902	0.685
Fe10	O12	1.921	0.650	Fe11	O20	2.079	0.419	Fe12	O24	2.070	0.430	Fe13	O20	2.077	0.422
Fe10	O26	2.104	0.391	Fe11	O23	1.920	0.652	Fe12	O25	1.927	0.639	Fe13	O27	1.931	0.632
Fe10	O27	1.907	0.676	Fe11	O24	2.083	0.415	Fe12	O28	2.085	0.412	Fe13	O28	2.105	0.390
Fe10	O53	2.034	0.475	Fe11	O47	2.030	0.480	Fe12	O50	2.043	0.463	Fe13	O51	2.018	0.497
Sum			3.030				3.035				2.997				3.059

**Compound 6**															
Bi1	O2	2.697	0.198												
Bi1	O3	2.705	0.194												
Bi1	O5'	2.413	0.412	Fe1	O1	2.039	0.468	Fe2	O4	2.033	0.476				
Bi1	O6	2.267	0.600	Fe1	O5	1.852	0.788	Fe2	O6	1.974	0.561				
Bi1	O7	2.287	0.570	Fe1	O5'	2.129	0.365	Fe2	O7	1.976	0.558	Fe3	O8	1.873	0.743
Bi1	O10	2.167	0.775	Fe1	O7	1.96	0.583	Fe2	O8	2.129	0.365	Fe3	O8	1.873	0.743
Bi1	O12	2.725	0.185	Fe1	O8	2.122	0.372	Fe2	O9	2.134	0.360	Fe3	O8	1.873	0.743
Bi1	O11	2.726	0.184	Fe1	O10'	2.175	0.321	Fe2	O10	1.854	0.783	Fe3	O9	1.876	0.737
Sum			3.118				2.897				3.103				2.965
**Compound 6a**															
Bi1	O2	2.788	0.157												
Bi1	O4	2.763	0.168												
Bi1	O5	2.187	0.736	Fe1	O3	2.033	0.476					Fe3	O1	2.039	0.468
Bi1	O6	2.253	0.622	Fe1	O5	2.079	0.419					Fe3	O5	2.187	0.311
Bi1	O7	2.27	0.595	Fe1	O6	1.983	0.547	Fe2	O8	1.837	0.821	Fe3	O7	1.983	0.547
Bi1	O9'	2.426	0.398	Fe1	O6	1.948	0.603	Fe2	O8	1.837	0.821	Fe3	O8'	2.11	0.385
Bi1	O10	2.657	0.220	Fe1	O7	1.969	0.569	Fe2	O8	1.837	0.821	Fe3	O9	1.85	0.792
Bi1	O11	2.643	0.228	Fe1	O8	2.123	0.371	Fe2	O8'	1.923	0.647	Fe3	O9'	2.171	0.325
Sum			3.124				2.986				3.110				2.827

6. IM-MS Measurements

To evaluate isomer stability and find out connection between structure type and cluster aggregation behaviour, we performed ion-mobility mapping mass spectrometry. Compounds **1-3**, **5**, **6** and **6a** showed the series of individual peaks within the region between *m/z* 3000 to 5000 of the IM-MS spectrum, which corresponds to $\{Bi_6Fe_{13}\}$ clusters with the different number of charges, ligands, and coordinating solvents, implying molecular oligomerization. The IM-MS spectra and complete peak assignments of compound **1-6** and **6a** are shown in **Table S5a-S5g**.

Evidence of supramolecular aggregation of the metal-organic polyhedra to give dimeric and trimeric structures is clear however no trend in drift time (i.e., aggregate structure) vs isomer symmetry was observed. Despite this, the gas phase behaviour for this system could be related to the long range structure in the solid state. For all of these compounds it is possible to assign regions representing monomeric $\{Bi_6Fe_{13}\}$ as well as corresponding dimers and trimers. Comparisons of the population of these clusters showed that compound **2** (L = $CHCl_2CO_2^-$) exhibits a more intense set of peaks representing a trimeric cluster while **3** (L = $CHF_2CO_2^-$) shows a more intense monomeric peak with **1** and **6** (L = $CF_3CO_2^-$ & $CClF_2CO_2^-$) preferentially forming a dimer (Figure S6i). On the face of it this does not appear to relate to either symmetry considerations described above, pKa or sterics of the ligands however upon close examination of the solid state structures insights can be found. The solid state structure of **3** contains no intramolecular bringing Na cations while the structure of **2** displays 12 distinct Na binding sites which are disordered over 24 positions. Compounds **1** and **6** each show 6 distinct binding sites with no disorder in the latter. As such, the level of intramolecular connectivity in the solid state seems to influence the gas phase behaviour suggesting a significant degree of stability in these structures under different conditions.

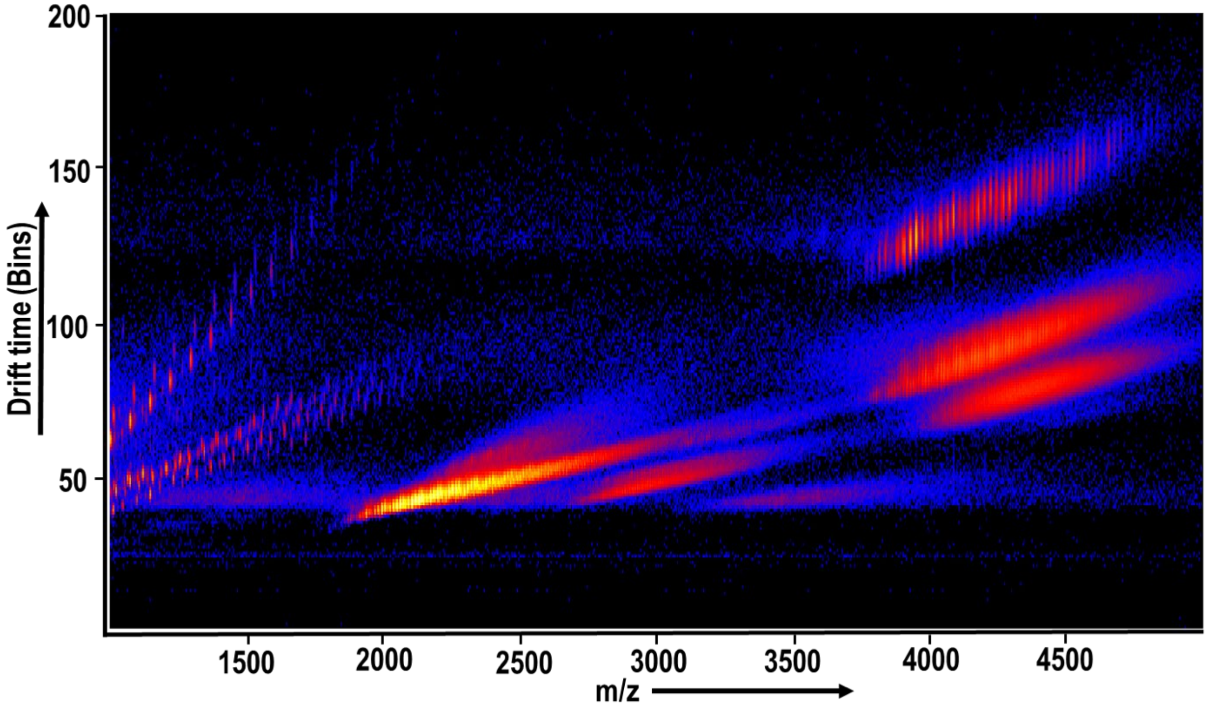

Figure S6a. IM-MS (+ve ion mode) mobilogram of compound **1** in CH_3CN

Table S5a. Peak assignments of compound **1** in CH_3CN

Species composition	Predicted *m/z*	Observed *m/z*
$\{Bi_6FeO_4Fe_{12}O_{12}(OH)_{13}(CF_3COO)_{11}(CF_3COOH)_1(CH_3CN)(H_2O)_4\}^{1+}$	3927.8	3928.8
$\{Bi_6FeO_4Fe_{12}O_{12}(OH)_{13}(CF_3COO)_{11}(CF_3COOH)_1(CH_3CN)_2(H_2O)_3\}^{1+}$	3950.9	3950.7
$\{Bi_6FeO_4Fe_{12}O_{12}(OH)_{14}(CF_3COO)_{10}(CF_3COOH)_3(CH_3CN)_1(H_2O)_3\}^{1+}$	4041.8	4042.8
$\{Bi_6FeO_4Fe_{12}O_{12}(OH)_{14}(CF_3COO)_{10}(CF_3COOH)_3(CH_3CN)_2(H_2O)_2\}^{1+}$	4064.8	4064.8
$\{Bi_6FeO_4Fe_{12}O_{12}(OH)_{14}(CF_3COO)_{10}(CF_3COOH)_4(CH_3CN)_2(H_2O)_2\}^{1+}$	4178.8	4178.8
$\{Bi_6FeO_4Fe_{12}O_{12}(OH)_{14}(CF_3COO)_{10}(CF_3COOH)_5(CH_3CN)_2(H_2O)_2\}^{1+}$	4292.8	4292.8
$\{Bi_6FeO_4Fe_{12}O_{12}(OH)_{15}(CF_3COO)_8(CF_3COOH)_5(CH_3CN)_3(H_2O)_1\}^{2+}$	2053.4	2054.8
$\{Bi_6FeO_4Fe_{12}O_{12}(OH)_{15}(CF_3COO)_8(CF_3COOH)_5(CH_3CN)_4\}^{2+}$	2064.9	2065.8
$\{Bi_6FeO_4Fe_{12}O_{12}(OH)_{15}(CF_3COO)_8(CF_3COOH)_6(CH_3CN)_4\}^{2+}$	2121.9	2122.8
$\{Bi_6FeO_4Fe_{12}O_{12}(OH)_{15}(CF_3COO)_8(CF_3COOH)_8(CH_3CN)_1(H_2O)_2\}^{2+}$	2192.4	2191.8
$\{Bi_6FeO_4Fe_{12}O_{12}(OH)_{15}(CF_3COO)_8(CF_3COOH)_9(CH_3CN)_1(H_2O)_3\}^{2+}$	2258.4	2258.8
$\{Bi_6FeO_4Fe_{12}O_{12}(OH)_{15}(CF_3COO)_8(CF_3COOH)_9(CH_3CN)_2(H_2O)_2\}^{2+}$	2269.9	2269.9
$\{Bi_6FeO_4Fe_{12}O_{12}(OH)_{15}(CF_3COO)_8(CF_3COOH)_{10}(CH_3CN)_2(H_2O)_2\}^{2+}$	2326.9	2326.8
$\{Bi_6FeO_4Fe_{12}O_{12}(OH)_{15}(CF_3COO)_8(CF_3COOH)_{11}(CH_3CN)_3(H_2O)_2\}^{2+}$	2404.4	2405.8

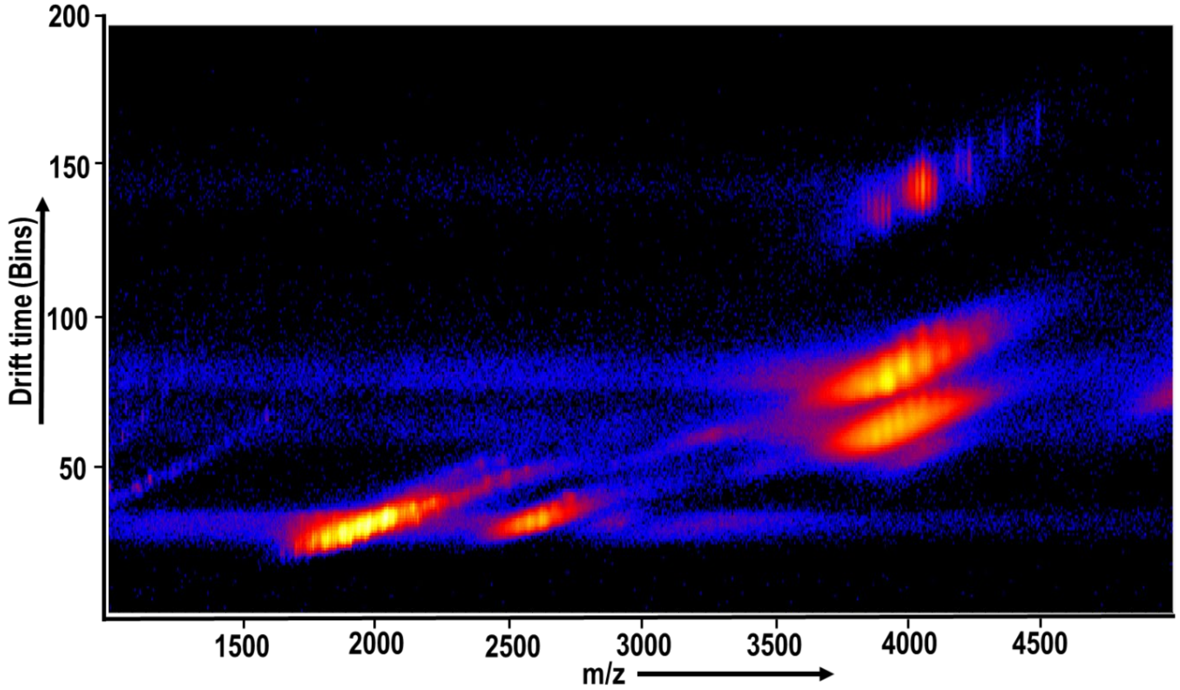

Figure S6b. IM-MS (+ve ion mode) mobilogram of compound **2** in CH_3CN

Table S5b. Peak assignments of compound **2** in CH_3CN

Species composition	Predicted *m/z*	Observed *m/z*
$\{Bi_6FeO_4Fe_{12}O_{12}(OH)_{13}(CCl_2HCOO)_{11}(H_2O)_4\}^{1+}$	3936.3	3936.1
$\{Bi_6FeO_4Fe_{12}O_{12}(OH)_{13}(CCl_2HCOO)_{11}(CH_3CN)_3(H_2O)_3\}^{1+}$	4041.3	4042.3
$\{Bi_6FeO_4Fe_{12}O_{11}(OH)_{15}(CCl_2HCOO)_{11}(HCCl_2COOH)(CH_3CN)_1(H_2O)_3\}^{1+}$	4105.2	4106.1
$\{Bi_6FeO_4Fe_{12}O_{10}(OH)_{17}(CCl_2HCOO)_{10}\}^{2+}$	1885.6	1884.6
$\{Bi_6FeO_4Fe_{12}O_{10}(OH)_{17}(CCl_2HCOO)_{10}(H_2O)\}^{2+}$	1894.6	1893.6
$\{Bi_6FeO_4Fe_{12}O_{10}(OH)_{17}(CCl_2HCOO)_{10}(CH_3CN)(H_2O)\}^{2+}$	1915.1	1915.6
$\{Bi_6FeO_4Fe_{12}O_{10}(OH)_{17}(CCl_2HCOO)_{10}(CH_3CN)(H_2O)_2\}^{2+}$	1924.2	1924.5
$\{Bi_6FeO_4Fe_{12}O_{10}(OH)_{17}(CCl_2HCOO)_{10}(HCCl_2COOH)\}^{2+}$	1950.6	1949.6
$\{Bi_6FeO_4Fe_{12}O_{10}(OH)_{17}(CCl_2HCOO)_{10}(HCCl_2COOH)(H_2O)_1\}^{2+}$	1959.6	1958.6
$\{Bi_6FeO_4Fe_{12}O_{10}(OH)_{17}(CCl_2HCOO)_{10}(HCCl_2COOH)(H_2O)_2\}^{2+}$	1968.6	1968.6
$\{Bi_6FeO_4Fe_{12}O_{10}(OH)_{17}(CCl_2HCOO)_{10}(HCCl_2COOH)(H_2O)(CH_3CN)\}^{2+}$	1980.1	1979.6
$\{Bi_6FeO_4Fe_{12}O_{10}(OH)_{17}(CCl_2HCOO)_{10}(HCCl_2COOH)(H_2O)_2(CH_3CN)\}^{2+}$	1989.1	1989.6
$\{Bi_6FeO_4Fe_{12}O_{10}(OH)_{17}(CCl_2HCOO)_{10}(HCCl_2COOH)_2(H_2O)(CH_3CN)\}^{2+}$	2044.1	2044.5
$\{Bi_6FeO_4Fe_{12}O_{10}(OH)_{17}(CCl_2HCOO)_{10}(HCCl_2COOH)_2(CH_3CN)(H_2O)_2\}^{2+}$	2053.1	2053.2
$\{Bi_6FeO_4Fe_{12}O_{10}(OH)_{17}(CCl_2HCOO)_{10}(HCCl_2COOH)_2(H_2O)(CH_3CN)_2\}^{2+}$	2064.6	2064.5

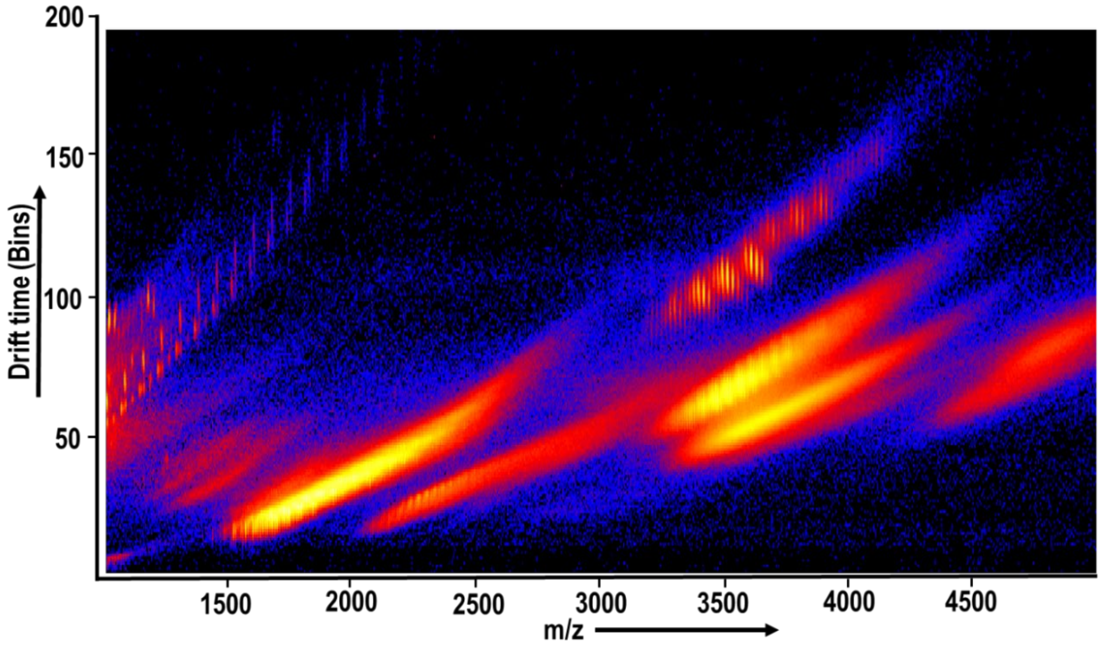

Figure S6c. IM-MS (+ve ion mode) mobilogram of compound **3** in CH_3CN

Table S5c. Peak assignments of compound **3** in CH_3CN.

Species composition	Predicted *m/z*	Observed *m/z*
$\{Bi_6FeO_4Fe_{12}O_{14}(OH)_{12}(CF_2HCOO)_8(CH_3CN)_2\}^{1+}$	3314.9	3315.0
$\{Bi_6FeO_4Fe_{12}O_{14}(OH)_{11}(CF_2HCOO)_9(CH_3CN)\}^{1+}$	3351.9	3353.0
$\{Bi_6FeO_4Fe_{12}O_{14}(OH)_{11}(CF_2HCOO)_9(CH_3CN)(H_2O)\}^{1+}$	3369.9	3371.0
$\{Bi_6FeO_4Fe_{12}O_{14}(OH)_{11}(CF_2HCOO)_9(CH_3CN)_2\}^{1+}$	3392.9	3393.0
$\{Bi_6FeO_4Fe_{12}O_{14}(OH)_{11}(CF_2HCOO)_9(CH_3CN)_2(H_2O)\}^{1+}$	3410.9	3411.0
$\{Bi_6FeO_4Fe_{12}O_{14}(OH)_{11}(CF_2HCOO)_9(CH_3CN)_3(H_2O)1\}^{1+}$	3452.0	3451.0
$\{Bi_6FeO_4Fe_{12}O_{14}(OH)_{10}(CF_2HCOO)_{10}(CH_3CN)_2(H_2O)\}^{1+}$	3488.9	3488.8
$\{Bi_6FeO_4Fe_{12}O_{14}(OH)_{10}(CF_2HCOO)_{10}(CH_3CN)_2(H_2O)_2\}^{1+}$	3506.9	3506.8
$\{Bi_6FeO_4Fe_{12}O_{12}(OH)_{14}(CF_2HCOO)_{10}(CH_3CN)_2\}^{1+}$	3506.9	3506.8
$\{Bi_6FeO_4Fe_{12}O_{12}(OH)_{14}(CF_2HCOO)_{10}(CF_2HCOOH)(CH_3CN)_2(H_2O)_1\}^{1+}$	3584.9	3584.9
$\{Bi_6FeO_4Fe_{12}O_{12}(OH)_{14}(CF_2HCOO)_{10}(CF_2HCOOH)(CH_3CN)_2(H_2O)_2\}^{1+}$	3602.9	3603.0
$\{Bi_6FeO_4Fe_{12}O_{15}(OH)_{11}(CF_2HCOO)_6(H_2O)_3\}^{2+}$	1547.9	1546.9
$\{Bi_6FeO_4Fe_{12}O_{15}(OH)_{11}(CF_2HCOO)_6(CH_3CN)_2\}^{2+}$	1561.9	1562.9
$\{Bi_6FeO_4Fe_{12}O_{15}(OH)_{11}(CF_2HCOO)_6(CH_3CN)_2(H_2O)\}^{2+}$	1571.0	1571.9
$\{Bi_6FeO_4Fe_{12}O_{14}(OH)_{12}(CF_2HCOO)_7(H_2O)_4\}^{2+}$	1604.9	1603.8
$\{Bi_6FeO_4Fe_{12}O_{14}(OH)_{12}(CF_2HCOO)_7(CH_3CN)_4\}^{2+}$	1651.0	1651.9
$\{Bi_6FeO_4Fe_{12}O_{14}(OH)_{11}(CF_2HCOO)_8(CH_3CN)_1(H_2O)_8\}^{2+}$	1700.0	1700.9
$\{Bi_6FeO_4Fe_{12}O_{14}(OH)_{10}(CF_2HCOO)_9(CH_3CN)_1(H_2O)_6\}^{2+}$	1721.5	1720.7

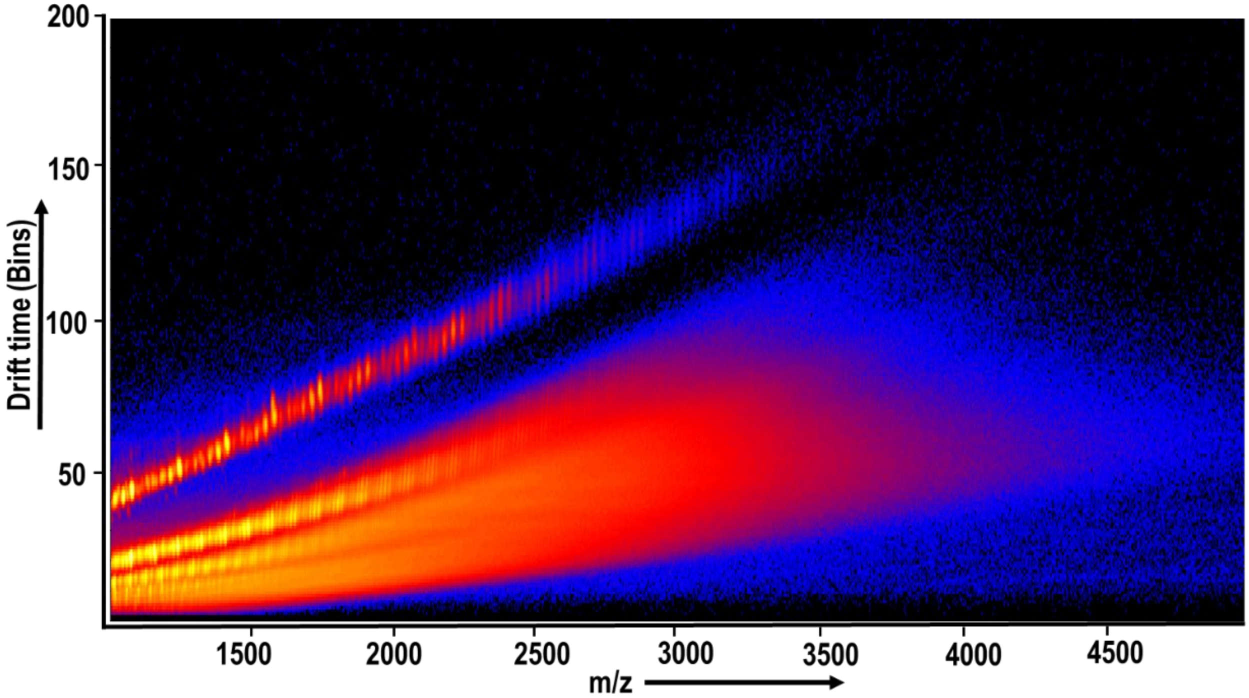

Figure S6d. IM-MS (+ve ion mode) mobilogram of compound **4** in MeOH

Table S5d. Peak assignments of compound **4** in MeOH

Species composition	Predicted *m/z*	Observed *m/z*
$\{Bi_3FeO_2Fe_6O_7(OH)_5(CClH_2COO)_6(H_2O)_2\}^{1+}$	1843.3	1843.1
$\{Bi_3FeO_2Fe_6O_7(OH)_5(CClH_2COO)_6(CH_3OH)(H_2O)_2\}^{1+}$	1875.3	1876.1
$\{Bi_3FeO_2Fe_6O_7(OH)_5(CClH_2COO)_6(CH_3OH)_2(H_2O)_2\}^{1+}$	1907.3	1907.1
$\{Bi_3FeO_2Fe_6O_6(OH)_5(CClH_2COO)_8(CH_3OH)_2\}^{1+}$	2043.3	2041.1
$\{Bi_3FeO_2Fe_6O_6(OH)_4(CClH_2COO)_9(CH_3OH)_2(H_2O)_3\}^{1+}$	2173.3	2173.0

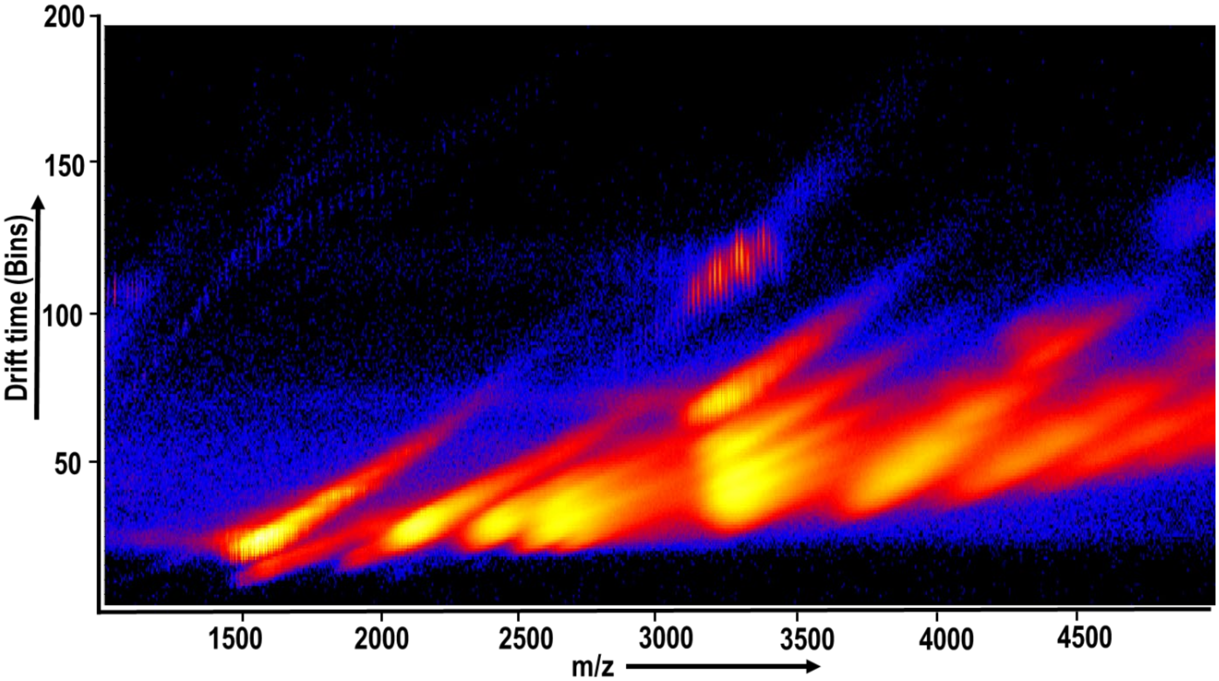

Figure S6e. IM-MS (+ve ion mode) mobilogram of compound (**5**) in MeOH

Table S5e Peak assignments of compound **5** in MeOH

Species composition	Predicted *m/z*	Observed *m/z*
$\{Bi_6FeO_4Fe_{12}O_{12}(OH)_{18}(CFH_2COO)_6(H_2O)_3\}^{1+}$	3059.0	3058.6
$\{Bi_6FeO_4Fe_{12}O_{12}(OH)_{17}(CFH_2COO)_7(H_2O)_5\}^{1+}$	3155.0	3154.7
$\{Bi_6FeO_4Fe_{12}O_{12}(OH)_{16}(CFH_2COO)_8(H_2O)_7\}^{1+}$	3251.1	3250.7
$\{Bi_6FeO_4Fe_{12}O_{12}(OH)_{14}(CFH_2COO)_{10}(CH_3OH)_2\}^{1+}$	3309.0	3308.8
$\{Bi_6FeO_4Fe_{12}O_{12}(OH)_{14}(CFH_2COO)_{10}(CH_3OH)_1(H_2O)_1\}^{1+}$	3327.0	3326.7
$\{Bi_6FeO_4Fe_{12}O_{12}(OH)_{12}(CFH_2COO)_{12}\}^{1+}$	3365.0	3364.7
$\{Bi_6FeO_4Fe_{12}O_{17}(OH)_8(CFH_2COO)_5(H_2O)_3\}^{2+}$	1446.0	1446.3
$\{Bi_6FeO_4Fe_{12}O_{17}(OH)_8(CFH_2COO)_5(CH_3OH)_2\}^{2+}$	1451.0	1451.8
$\{Bi_6FeO_4Fe_{12}O_{17}(OH)_8(CFH_2COO)_5(CH_3OH)_2(H_2O)\}^{2+}$	1460.0	1460.8
$\{Bi_6FeO_4Fe_{12}O_{17}(OH)_7(CFH_2COO)_6(CH_3OH)_2(H_2O)\}^{2+}$	1490.0	1490.8
$\{Bi_6FeO_4Fe_{12}O_{17}(OH)_7(CFH_2COO)_6(CH_3OH)_3(H_2O)_2\}^{2+}$	1515.0	1515.3
$\{Bi_6FeO_4Fe_{12}O_{16}(OH)_7(CFH_2COO)_8(CH_3OH)_2(H_2O)_1\}^{2+}$	1559.0	1558.8
$\{Bi_6FeO_4Fe_{12}O_{16}(OH)_7(CFH_2COO)_8(CH_3OH)_1(H_2O)_4\}^{2+}$	1570.0	1570.8
$\{Bi_6FeO_4Fe_{12}O_{16}(OH)_6(CFH_2COO)_9(CH_3OH)_2(H_2O)_2\}^{2+}$	1598.0	1597.8

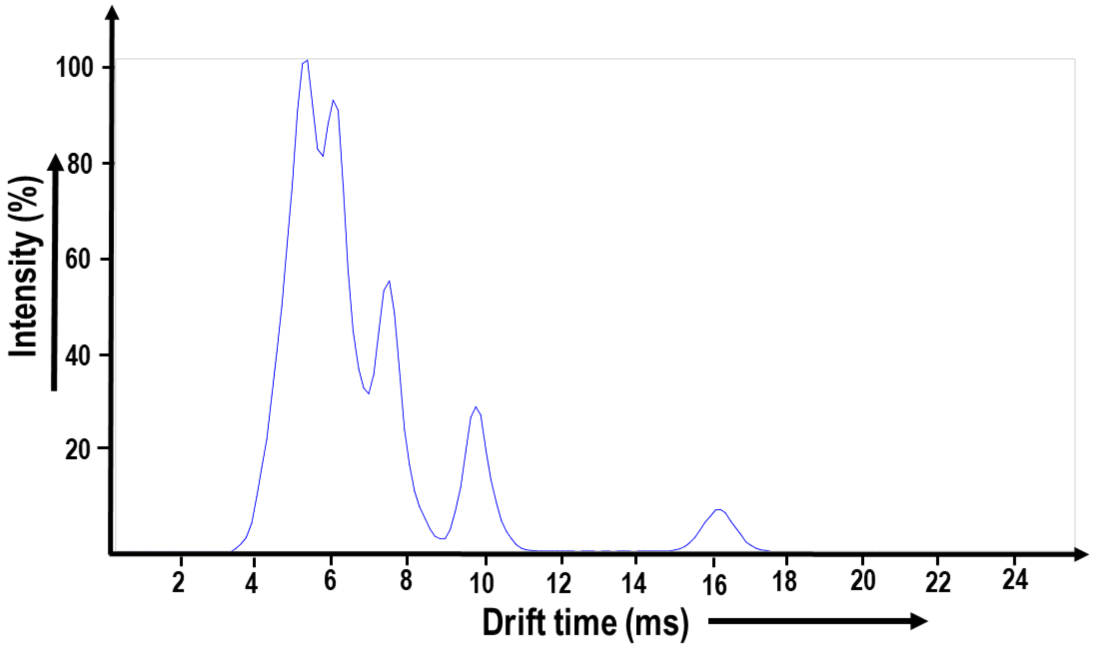

Figure S6f. Drift time vs intensity of compound **5** in MeOH (m/z ~3268) in IM-MS [Possible composition $\{Bi_6FeO_4Fe_{12}O_{12}(OH)_{16}(CFH_2COO)_8(H_2O)_8\}^{1+}$].

Compound **5** displays a remarkable difference regarding the aggregation in the gas phase, as compared to other derivatives, **1-3**, **6**, and **6a**, and the spectrum is depicted in **Figure S6e** and **S6f**. The former show gigantic supramolecular aggregation *i.e.,* $\{Bi_6Fe_{13}\}$ cluster-based pentamers whereas the latter clusters show mainly $\{Bi_6Fe_{13}\}$ cluster-based trimers. This is probably because the sterically smaller carboxylate ligand ($CFH_2CO_2^-$) associated with $\{Bi_6Fe_{13}\}$ clusters in compound **5** exhibits rich intermolecular interaction (H..F and F..F) between the individual clusters in the solid-state. As a result, compound **5** is densely packed in the solid-state, which gives low volume per cluster (**Table S2**) and consequently affects the solubility. Due to the overlapping nature of associated spots in real-time, especially $\{Bi_6Fe_{13}\}$ based tetramer and pentamer clusters, we were not able to assign the unique composition of the clusters but the aggregation was clearly confirmed by drift time.

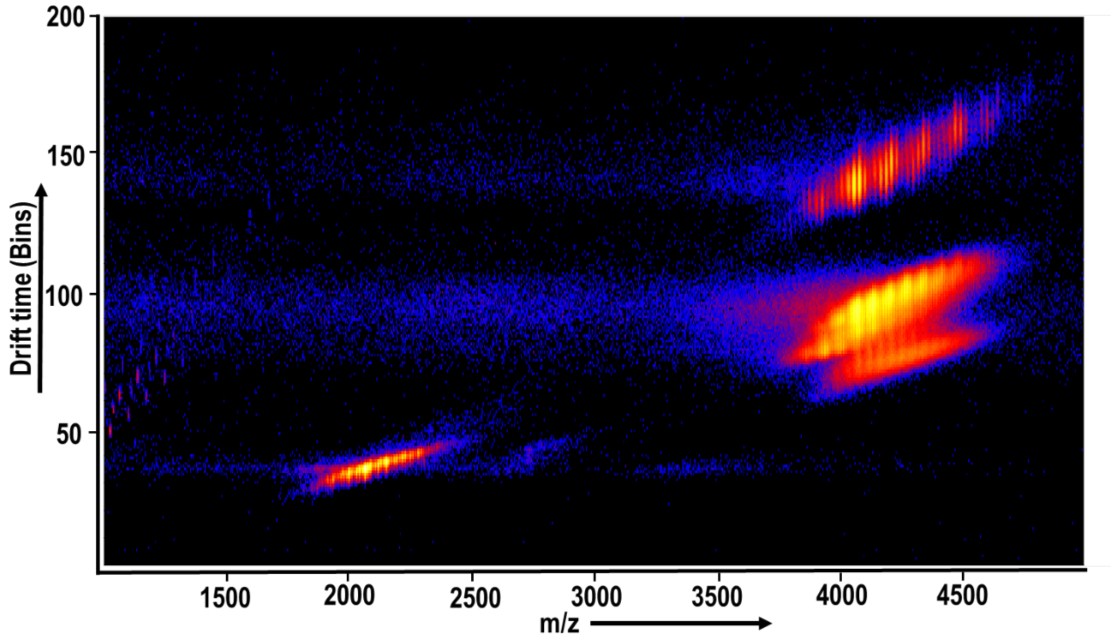

Figure S6g. IM-MS (+ve ion mode) mobilogram of compound **6** in CH_3CN

Table S5f. Peak assignments of compound **6** in CH_3CN

Species composition	Predicted *m/z*	Observed *m/z*
$\{Bi_6FeO_4Fe_{12}O_{15}(OH)_9(CClF_2COO)_9(CClF_2COONa)_1(CH_3CN)_3(H_2O)_4\}^{1+}$	3949.6	3950.9
$\{Bi_6FeO_4Fe_{12}O_{14}(OH)_{10}(CClF_2COO)_{10}(CClF_2COONa)_1(H_2O)_4(CH_3CN)_3\}^{1+}$	4079.5	4080.9
$\{Bi_6FeO_4Fe_{12}O_{14}(OH)_{10}(CClF_2COO)_{10}(CClF_2COONa)_1(H_2O)_3(CH_3CN)_4\}^{1+}$	4102.6	4102.9
$\{Bi_6FeO_4Fe_{12}O_{12}(OH)_{13}(CClF_2COO)_{11}(CClF_2COONa)_1(H_2O)_2(CH_3CN)_3\}^{1+}$	4191.5	4190.9
$\{Bi_6FeO_4Fe_{12}O_{12}(OH)_{13}(CClF_2COO)_{11}(CClF_2COONa)_1(H_2O)_3(CH_3CN)_3\}^{1+}$	4209.5	4210.9
$\{Bi_6FeO_4Fe_{12}O_{12}(OH)_{12}(CClF_2COO)_{12}(CClF_2COONa)_1(H_2O)_4(CH_3CN)_3\}^{1+}$	4341.5	4342.9
$\{Bi_6FeO_4Fe_{12}O_{12}(OH)_{12}(CClF_2COO)_{12}(CClF_2COONa)_1(H_2O)_3(CH_3CN)_4\}^{1+}$	4364.5	4362.9
$\{[(Bi_6FeO_4Fe_{12}O_{15}(OH)_{10}(CClF_2COO)_8)(Bi_6FeO_4Fe_{12}O_{12}(OH)_{13}(CClF_2COO)_{11})]$ $(CClF_2COONa)_4(CH_3CN)_3(H_2O)_2\}^{2+}$	4069.9	4070.9

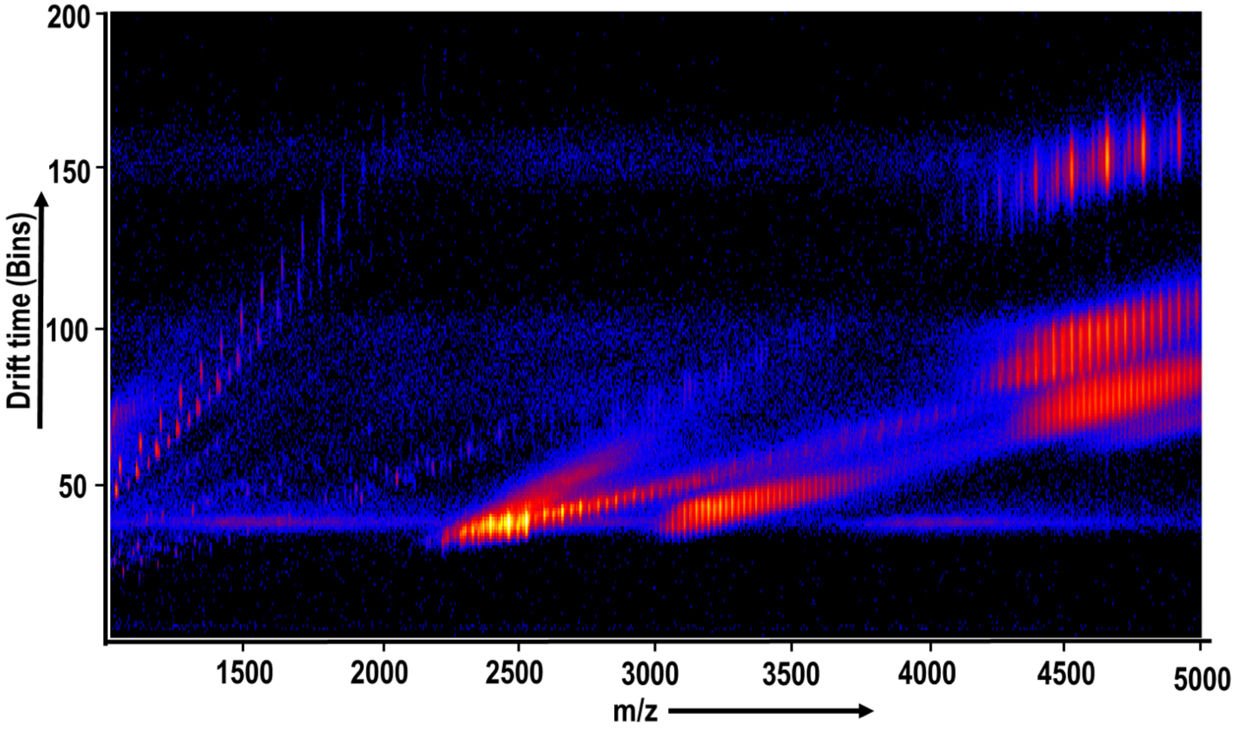

Figure S6h. IM-MS (+ve ion mode) mobilogram of compound **6a** in CH_3CN

Table S5g. Peak assignments of compound **6a** in CH_3CN

Species composition	Predicted *m/z*	Observed *m/z*
$\{Bi_6FeO_4Fe_{12}O_{12}(OH)_{12}(CClF_2COO)_{12}(CClF_2COOCs)_1\}^{1+}$	4256.3	4256.4
$\{Bi_6FeO_4Fe_{12}O_{12}(OH)_{12}(CClF_2COO)_{12}(CClF_2COOCs)_2(CClF_2COOH)\}^{1+}$	4648.1	4648.5
$\{(Bi_6FeO_4Fe_{12}O_{12}(OH)_{12}(CClF_2COO)_{12})_2(CClF_2COOCs)_4(CClF_2COOH)_2\}^{2+}$	4648.1	4649.9
$\{(Bi_6FeO_4Fe_{12}O_{12}(OH)_{12}(CClF_2COO)_{12})_3(CClF_2COOCs)_6(CClF_2COOH)_3\}^{3+}$	4648.7	4649.6

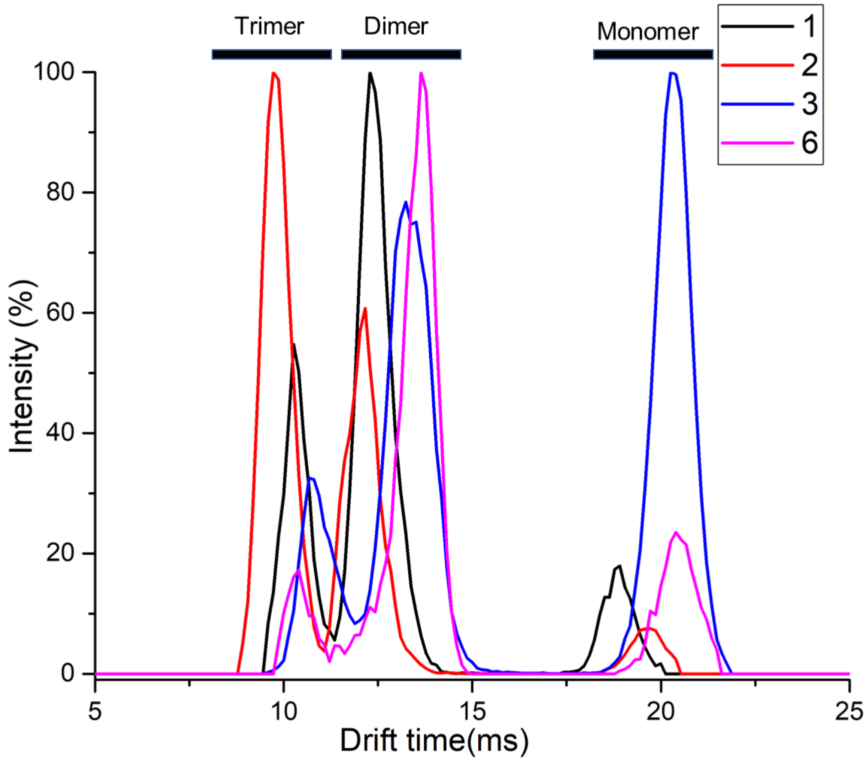

Figure S6i: Drift time vs intensity of compound **1** (*m/z* ~ 4294), **2** (*m/z* ~ 4042), **3** (*m/z* ~ 3506) and **6** (*m/z* ~ 4342) in CH_3CN in IM-MS (Possible composition is given in the **Table 5a-c** and **f**)

7. Collison Cross Section (CCS) Studies of 6a

Unlike high-resolution ESI-MS, the intrinsic nature of the IM-MS instrument, together with multiple instrument settings and tunable experimental parameters, enable us to investigate and provide solution for separation of different sizing of clusters. However, it is not practical to determine Collision Cross Section (CCS) values directly as such from IM-MS instruments, instead, it must be obtained through calibration from known species. By calculating the CCS values of isobaric ions, we may be able to confirm that whether each signal comes from oligomerization of single species or experimental artifacts aggregation of the charged species in spray, and thus, one may be able to scrutinize the formation of the target compounds. Also, the buffer gas (both N_2 and He) has a potential driving force on predicting CCS values, a careful precaution is required for calibration.

We herein employed Water Synpat instruments with drift tube cell has N_2 buffer gas, and $[Na][NaI]_n$ (where n = 1-17) calibrant was used. The CCS values of $[Na][NaI]_n$ clusters were calibrated according to Mclean's online CCS database.[11] Using NaI as CCS calibrant, we estimated the CCS of Bi-Fe oxo clusters. Note, we chose compound **6a**, compared to the rest of the compounds, because the ligand associated within this complex has very low pKa (**Table S1**), and has a well-defined crystal structure. Furthermore, **6a** has been recrystallized it from acetonitrile and has strong interaction with counter cations and solvent molecules in the solid-state, which allows it to be extremely solution stable. 40 mg of **6a** was dissolved in 1ml CH_3CN, gives a deep red colour solution. This was directly injected to Waters Synpat instrument and spectrum was collected in the resolution mode. The drift time of different $[\{Bi_6Fe_{13}O_{16}(OH)_{12}(L)_{12}\}_x(CsL)_y(HL)_z]^{n+}$, where L = $CClF_2CO_2^-$, n, x and z = 1-3, y = 2-6) based fragments with similar *m/z* (~ 4649.0) was observed to confirm that each of these three major spots are single species (Figure 7) and thus, the CCS of these aggregates was calculated (**Table 1**). As predicted by their assignment the CCS value for the dimer was double that of the monomer with the trimer CCS around three times that of the isolated cluster. We have carried out the theoretical calculations of CCS for the monomer species $\{Bi_6Fe_{13}O_{16}(OH)_{12}(L)_{12}(CsL)_2(HL)\}$ using a structure model built from crystal structures of **6a** assuming the attachments $(CsL)_2(HL)$ are closely located to the main cluster. The traditional Mobcal approach was adjusted to suit inorganic clusters[12] to include additional elements Fe and Bi and this produced CCS_{Calc} values (eg. ca. 388 $Å^2$ from the Projection Approximation method) close to the experimentally-determined CCS (**Table 1**). The CCS values were precisely predicted by comparing with NaI calibrants.

8. Infrared Spectroscopy (IR)

The Fourier Transform Infrared (FT-IR) spectroscopy of **1-6** and **6a** reveals that several distinct bands between 1500 - 500 cm^{-1} which can be considered as the fingerprint region of the metal-oxo clusters, showing the stretching modes of bismuth-oxo bonds (940-945cm^{-1}) the carbonyl bands (1620-30 cm^{-1}), the Fe-O stretching band (650-700 cm^{-1}). Since **6** and **6a** has difference only on cations, we presented only **6a** here. Also, **Keggin-2**, **1** and **1'** have same core structure, we presented **1'** FT-IR here. Elemental and ICP analysis results also supports the formulation of compound **1-6** and **6a.** Though, metal analysis, both Fe and Bi, percentage was very low, the ratio between Fe and Bi was confirmed 13:6.

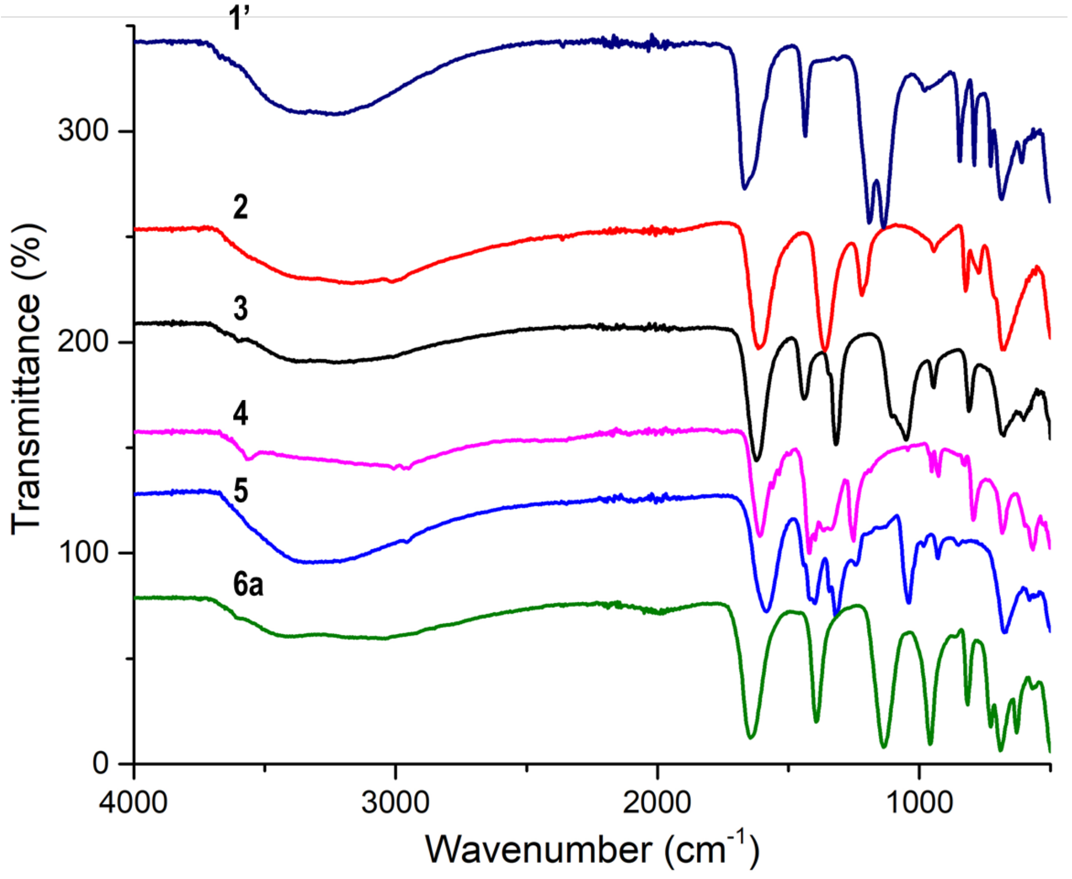

Figure S7. ATR spectra of Compounds **1-6**

9. Thermogravimetric Analysis (TGA)

The thermal stability of compounds **1-6** was investigated by thermogravimetric analysis, and the thermogram of **1-6** is given in the **Figures S8a-S8h**. For the **1-6**, the first weight loss step until ~180ºC is associated with surface dehydration, followed by second weight loss step till ~300ºC associated with decomposition of acetate molecules, and the final weight loss after ~300ºC corresponding to the decomposition of bismuth-iron oxo clusters.

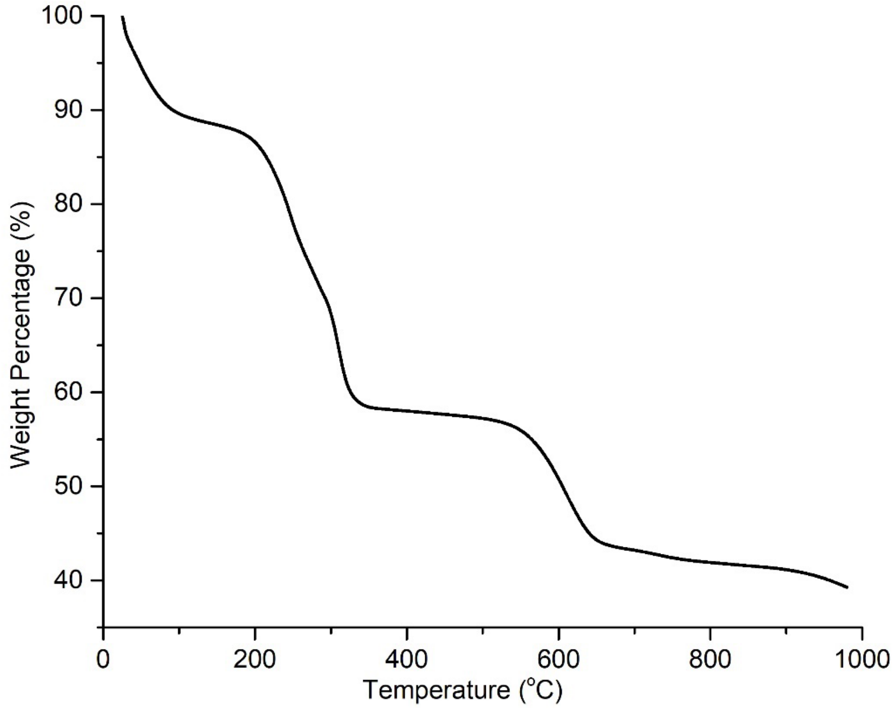

Figure S8a. TGA result of $Na_3[Bi_6Fe_{13}O_{16}(OH)_{12}(CF_3COO)_{12}](CF_3COO)_4{\cdot}36H_2O$ (**1**)

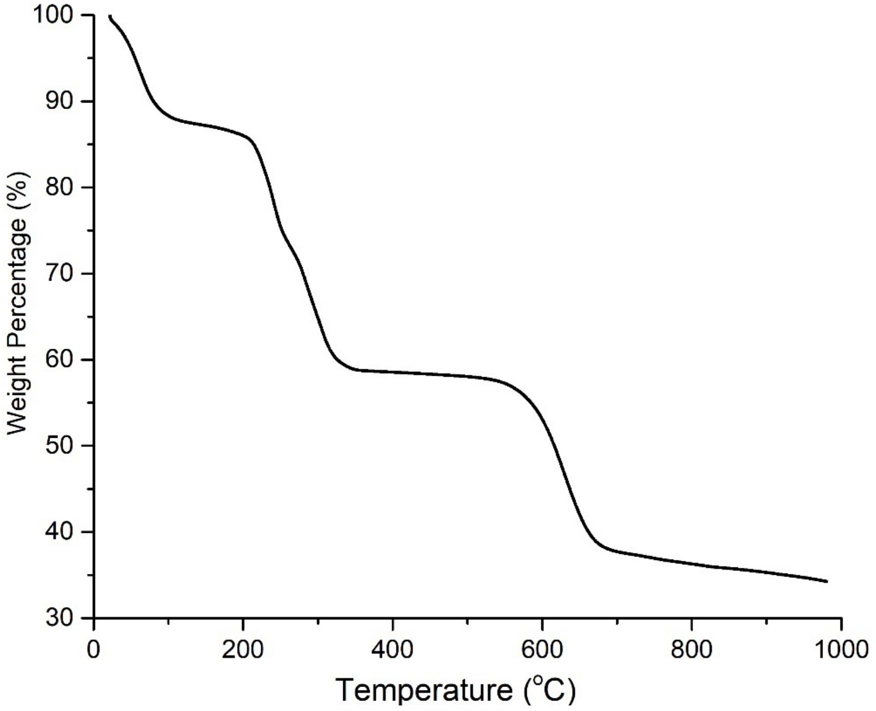

Figure S8b. TGA result of $Na_3[Bi_6Fe_{13}O_{16}(OH)_{12}(CF_3COO)_{12}](CF_3COO)_4 \cdot 40H_2O$ (**1'**)

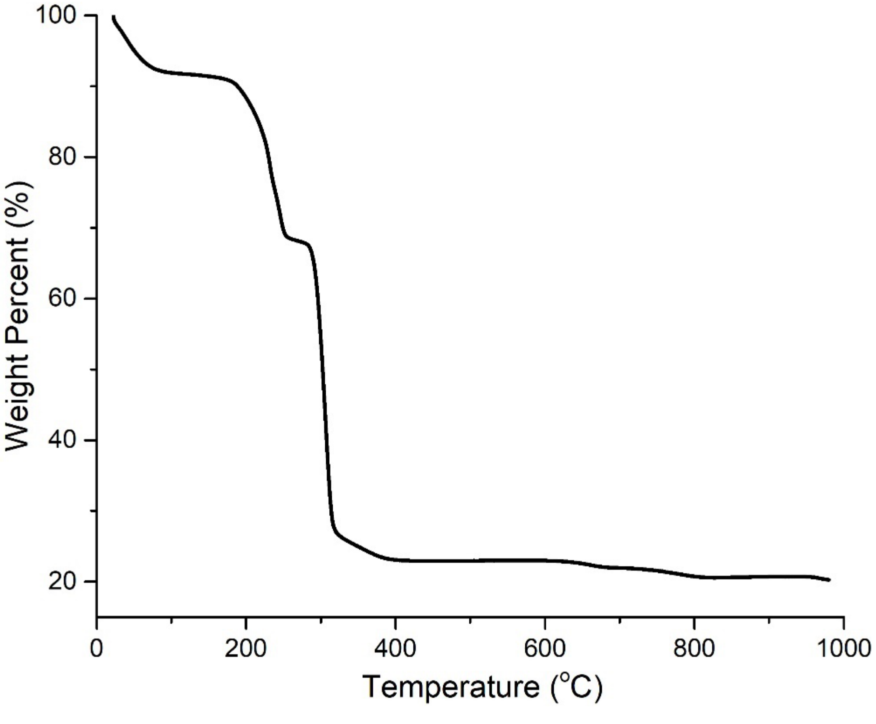

Figure S8c. TGA result of $Na_2[Bi_6Fe_{13}O_{16}(OH)_{12}(CCl_2HCOO)_{12}](CCl_2HCOO)_3 \cdot 31H_2O$ (**2**)

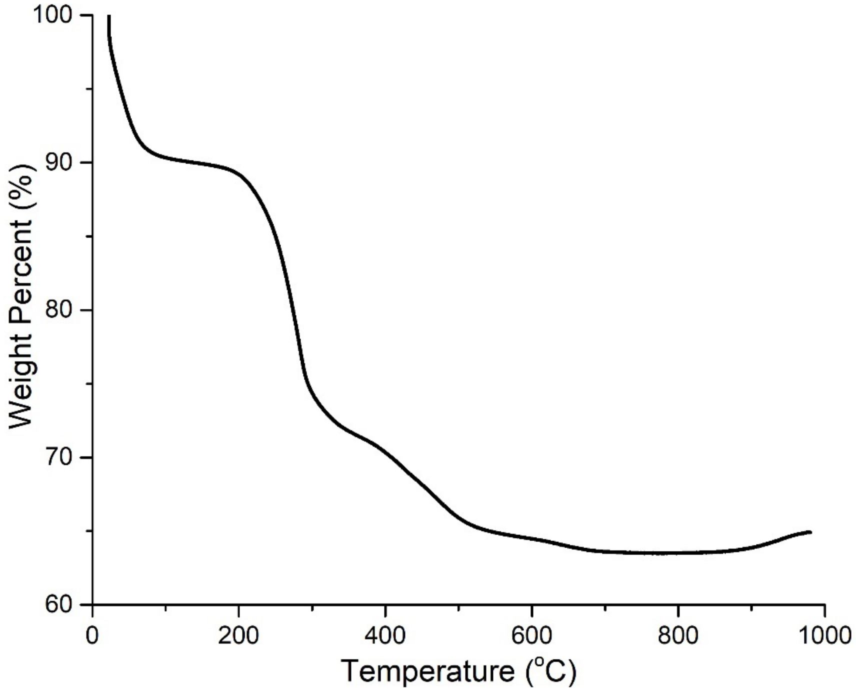

Figure S8d. TGA result of $Na[Bi_6Fe_{13}O_{16}(OH)_{12}(CF_2HCOO)_{12}](CF_2HCOO)_2 \cdot 12H_2O$ (**3**)

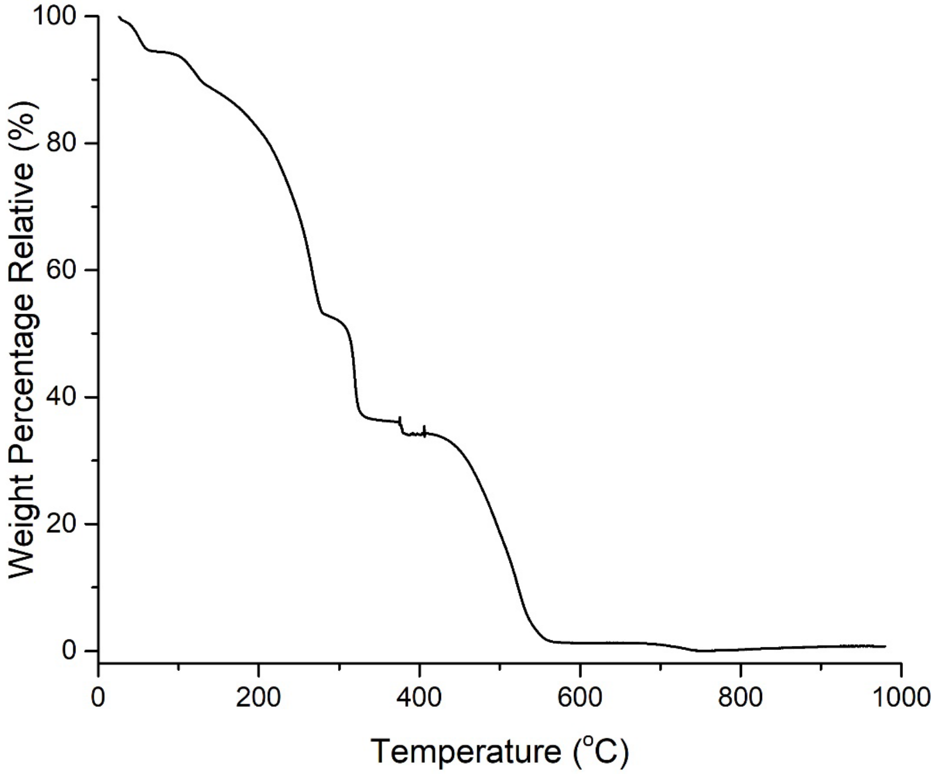

Figure S8e. TGA result of $Na_2[Bi_6Fe_{13}O_{16}(OH)_{12}(CClH_2COO)_{12}](CClH_2COO)_3 \cdot 15H_2O$ (**4**)

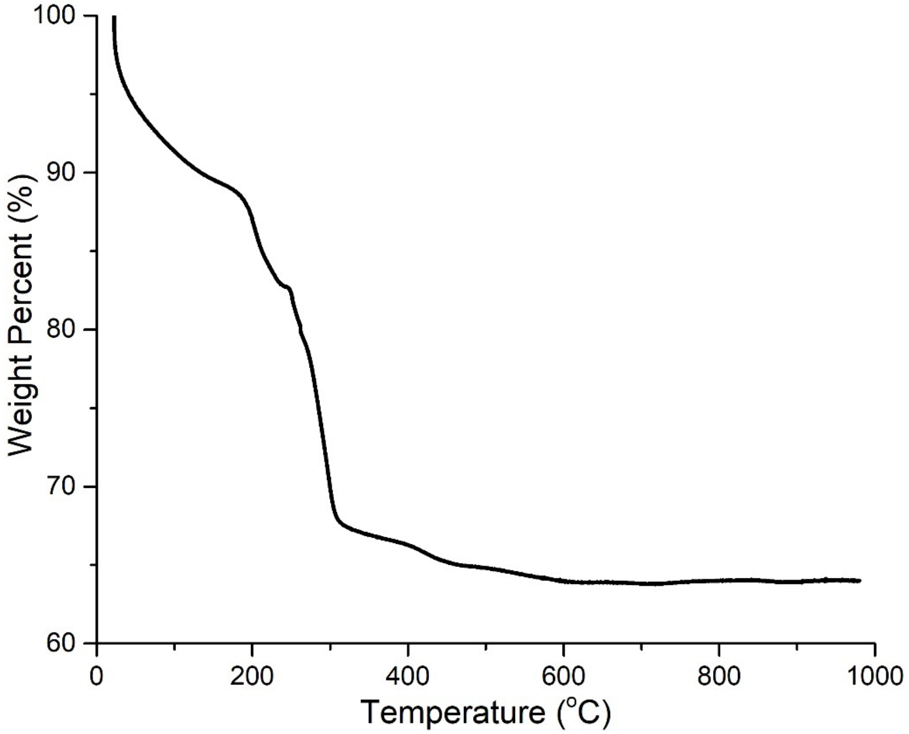

Figure S8f. TGA result of $Na[Bi_6Fe_{13}O_{16}(OH)_{12}(CFH_2COO)_{12}](NO_3)_2 \cdot 23H_2O$ (**5**)

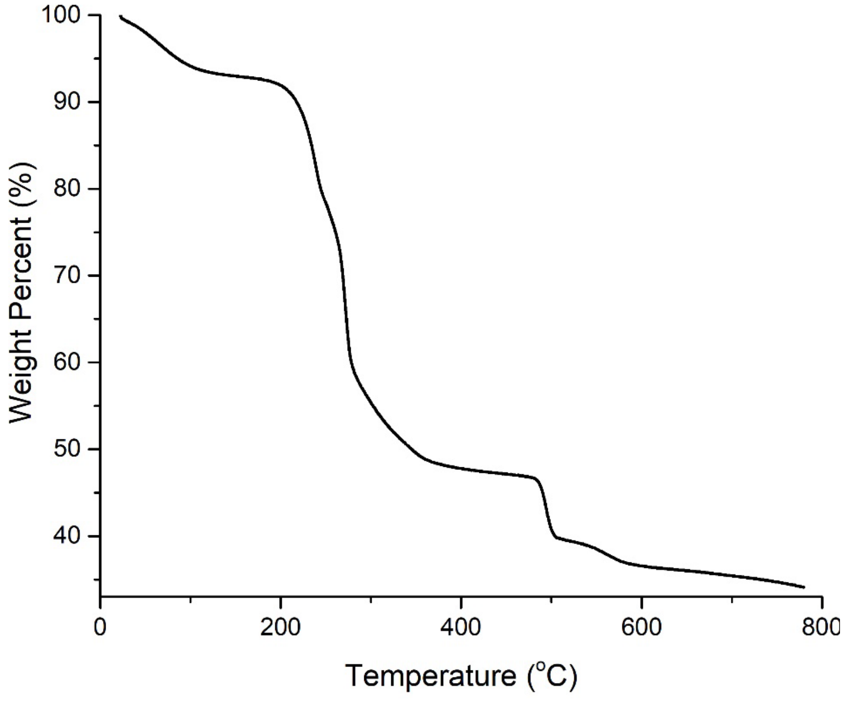

Figure S8g. TGA result of $Na_4[Bi_6Fe_{13}O_{16}(OH)_{12}(CClF_2COO)_{12}](CClF_2COO)_5 \cdot 3CH_3CN \cdot 15H_2O$ (**6**)

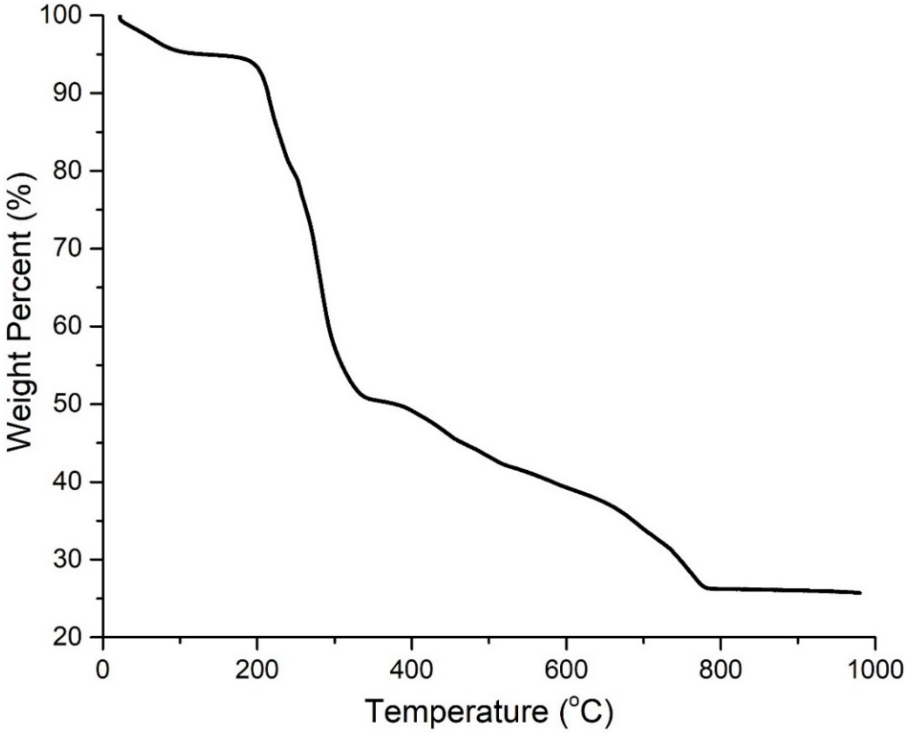

Figure S8h. TGA result of $Cs_4[Bi_6Fe_{13}O_{16}(OH)_{12}(CClF_2COO)_{12}]$ $(CClF_2COO)_5{\cdot}3CH_3CN{\cdot}10H_2O$ (**6a**)

10. References

1. Sheldrick, G. M.; SHELXTL Version 2018. http://shelx.uni-ac.gwdg.de/SHELX/index.php
2. Farrugia L.; WinGX suite for small-molecule single-crystal crystallography, *J. Appl. Cryst*., **1999**, *32*, 837-838.
3. Clark R. C.; Reid J. S.; The analytical calculation of absorption in multifaceted crystals, *Acta Cryst., Sect. A*,**1995**, *51*, 887-897.
4. Liu, B.; Zhou, W.-W.; Zhou, Z.-Q.; Zhang, X.-Y.; Hydrolysis to the first dumbbell-like high-nuclearity bismuth-oxo cluster $[Bi_{12}(\mu_3\text{-}OH)_4(\mu_2\text{-}OH)^2(\mu_3\text{-}O)_8(\mu_4\text{-}O)_2(NO_3)_6]^{4+}$: Synthesis, structure and spectroscopic characterizations, *Inorg. Chem. Comm,* **2007**, *10*, 1145-1148.
5. Sadeghi, O.; Amiri, A.; Reinheimer, E. W.; Nyman, M.; The Role of Bi^{3+} in Promoting and Stabilizing Iron Oxo Clusters in Strong Acid, *Angew. Chem. Int. Ed.* **2018**, *57*, 6247-6250.
6. Yangjeh, A. H.; Jenagharad, M. D.; Prediction of acidity constant for substituted acetic acids in water using artificial neural networks, *Indian J. Chem.,***2007**, *46B*, 478-487
7. Sadeghi, O.; Zakharov, L. N.; Nyman, M.; Aqueous formation and manipulation of the iron-oxo Keggin ion, *Science* **2015**, *347*, 1359-1362.
8. Sadeghi, O.; Falaise,C.; Molina, P. I.; Hufschmid, R.; Campana, C. F.; Noll, B. C.; Browning, N. D.; Nyman, M.; Chemical Stabilization and Electrochemical Destabilization of the Iron Keggin Ion in Water, *Inorg. Chem.* **2016**, *55*, 11078-11088.
9. Petrucci, Harwood, Herring, and Madura. General Chemistry: Principles and Modern Applications: 9th Edition. New Jersey: Pearson, **2007**.
10. Gagne, O. C.; Hawthorne, F. C.; Comprehensive derivation of bond-valence parameters for ion pairs involving oxygen, *Acta Cryst.* **2015**, *B71*, 561-578.
11. https://mcleanresearchgroup.shinyapps.io/CCS-Compendium/
12. Xuan, W.; Surman, A. J.; Miras, H. N.; Long, D.-L.; Cronin, L., Controlling the Ring Curvature, Solution Assembly, and Reactivity of Gigantic Molybdenum Blue Wheels, *J. Am. Chem. Soc.* **2014,** *136*, 14114-14120.
